# Greener and more sustainable access to 6,7-dimethoxy-2-aryl-4-aminoquinazolines *via* microwave-assisted chemo-selective synthesis in bio-renewable solvents

**DOI:** 10.1039/d6ra08343a

**Published:** 2026-11-03

**Authors:** Rhushikesh C. Deokar, Thrilokraj R, Nutan Sharma, Dhananjay Satav, Markus Obkircher, Sadaf Jamal Gilani, Saikat Sinha, Ramesh B. Dateer

**Affiliations:** a Sigma Aldrich Chemicals Pvt Ltd #12, Bommasandra-Jigani Link Road Bangalore-560100 India saikat.sinha@merckgroup.com; b Centre for Nano and Material Sciences, JAIN (deemed-to-be) University Jain Global Campus, Kanakapura Road Bangalore-562112 India d.ramesh@jainuniversity.ac.in; c Department of Chemistry, School of Basic and Applied Sciences, SGT University Gurugram-122505 Haryana India; d Department of Pharmaceutical Sciences, College of Pharmacy, Princess Nourah Bint Abdulrahman University P. O. Box 84428 Riyadh 11671 Saudi Arabia

## Abstract

Developing more sustainable, efficient and environmentally benign protocols towards the construction of promising heterocycles remains a cornerstone of green and sustainable medicinal chemistry. Herein, a two-stage, microwave-assisted, rapid and greener synthesis of 6,7-dimethoxy-2-aryl-4-aminosubstituted-quinazoline derivatives is demonstrated. Chemoselective SNAr at C-4 position of 2,4-dichloro-6,7-dimethoxyquinazoline with primary amines in bio-renewable γ-valerolactone (GVL) solvent delivered 4-amino-2-chloroquinazoline intermediates. Subsequently, a palladium-catalyzed Suzuki–Miyaura cross-coupling at C-2 using ethanol–water (9 : 1) as an eco-friendly medium under microwave irradiation at 100 °C for 120 min, furnished 2-aryl-4-aminosubstituted quinazoline derivatives. Relative to a conventional solvent benchmark for step 2, the optimized platform achieves a 4.8-fold reduction in reaction-step simple E-factor reduction from 18.4 to 3.8 kg kg^−1^, enhancement in reaction-step process mass intensity to 5.2-fold (8.2 g g^−1^) from 42.6 g g^−1^, 80% reduction in reaction time (120 from 480–840 minutes) and achieving an Intrinsic atom economy of 82.2%, addressing seven out of the twelve Anastas–Warner green chemistry principles. This workflow underscores a scalable, solvent-efficient, metal-catalyzed route with reduced environmental impact.

## Abstract 2

Overview of the MW-accelerated greener synthesis. Top: two-step sequential synthesis flow (step 1: SNAr at C-4 in GVL/DIPEA, MW, 80 °C; step 2: Suzuki–Miyaura at C-2 in EtOH–H_2_O/PdCl_2_(PPh_3_)_2_, MW, 100 °C); Bottom: key greener chemistry metrics.

## Graphical abstract

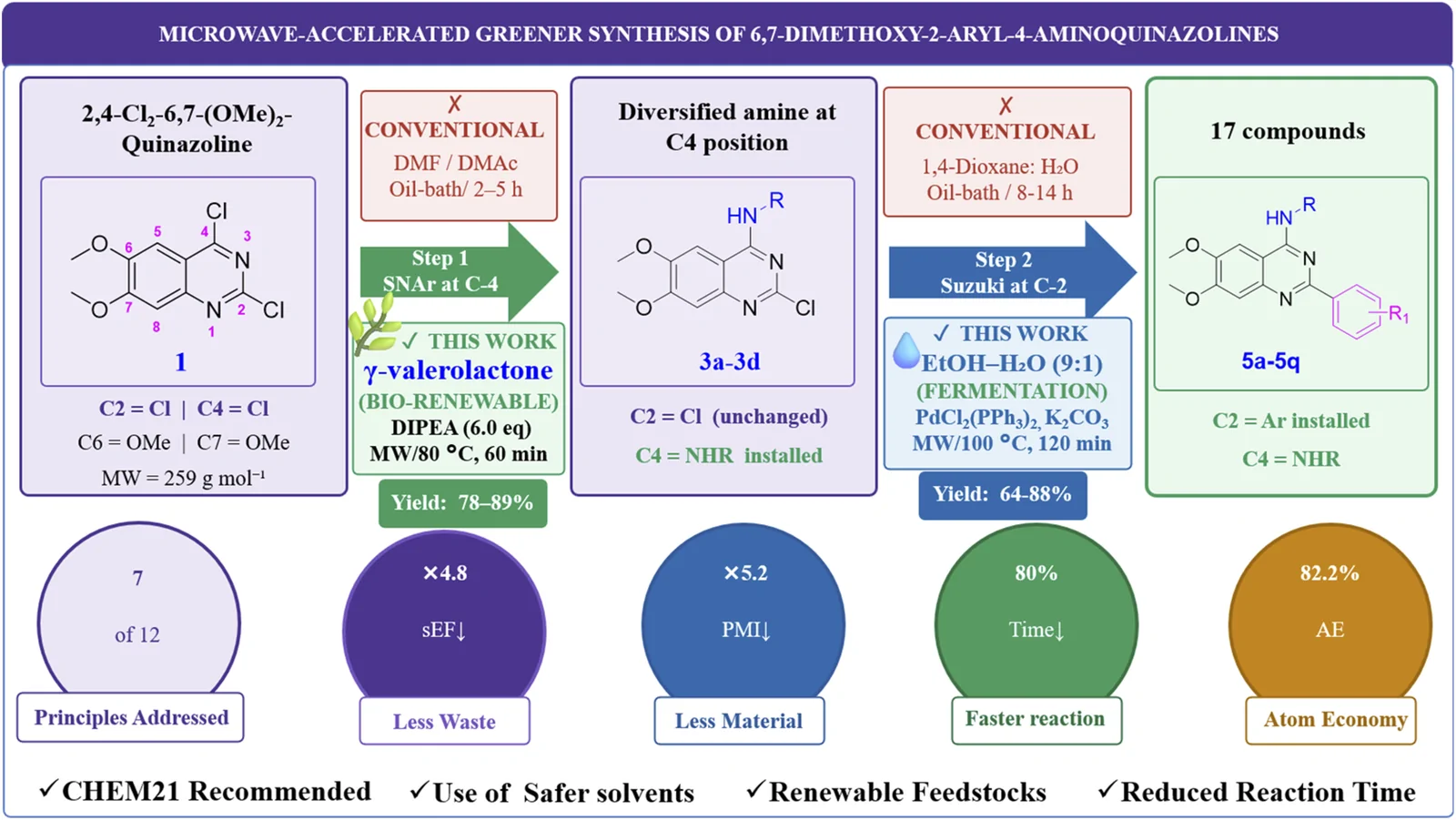

## 1. Introduction

In the pursuit of environmentally benign chemical manufacturing, the search for greener alternatives to hazardous organic solvents remains the cornerstone of modern synthetic chemistry.^1^ Crucial targets for eco-friendly synthetic protocols involve the use of alternative reaction media, catalyst recyclability and waste minimization, all driven by the need to improve process safety and reduce environmental impact.^2^ The search for non-hazardous reaction media has directed special attention towards the solvents derived from bio-renewable raw materials due to their negligible or no impact on health and the environment.^3^ Among the emerging class of renewable, bio-based solvents, γ-valerolactone (GVL) has emerged as a frontrunner alternative to conventional dipolar aprotic solvents such as *N*,*N*-dimethyl formamide (DMF), *N*,*N*-dimethyl acetamide (DMAc) and *N*-methyl-2-pyrrolidone (NMP), owing to its favorable physicochemical properties, including high boiling point, low toxicity, excellent chemical stability and complete biodegradability (OECD 301B). Its dielectric constant (*ε* ≈ 36) and dipole moment (*µ* ≈ 4.2 D) closely resemble DMF, confirming adequate solvation capacity for polar organic transformations.^4,5^ GVL has demonstrated broad utility as a reaction medium to access active pharmaceutical entities, highlighting its broad potential in heterocyclic synthesis.^6^

However, the quinazoline scaffold stands as one of the most therapeutically productive bicyclic nitrogen heterocycles in contemporary medicinal chemistry, distinguished by its remarkable capacity to engage structurally disparate biological targets across multiple disease areas.^7^ Embedded in more than twenty clinically approved small-molecule drugs spanning cardiovascular medicine (Prazosin, Doxazosin, Terazosin), oncology (Gefitinib, Erlotinib, Lapatinib, Afatinib, Osimertinib) and anti-infective pharmacotherapy this scaffold has long exemplified the concept of a privileged structure in drug discovery (Fig. 1a). Its exceptional pharmacological breadth arises from the intrinsic capacity of the N-1 and N-3 nitrogen atoms to engage in hydrogen-bond donation and acceptance with enzyme active-site residues, the planar aromatic framework to sustain π–π stacking within hydrophobic recognition pockets and the C-2 and C-4 substituents to project into structurally diverse lipophilic sub-pockets with substantial electronic and steric tunability.^7^

**Fig. 1:**
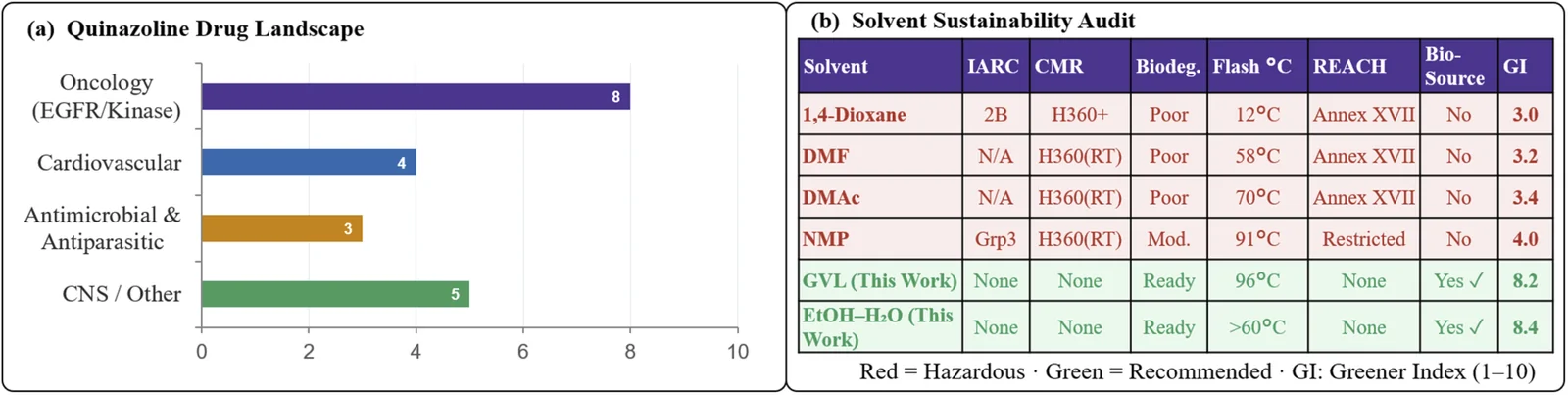
(a) Quinazoline-based approved drugs by therapeutic area (b) solvent sustainability audit.

Within the broader quinazoline chemical space, the 4-aminoquinazoline subclass has demonstrated broad pharmacological profile across mechanistically distinct therapeutic targets (Table 1). In epigenetic oncology, the 6,7-dimethoxy-4-aminoquinazoline scaffold forms the structural core of canonical G9a/GLP (EHMT1/2) histone methyltransferase inhibitors (BIX-01294, UNC0638 and their successors) and has been deployed in PROTAC-based G9a/GLP degraders active in breast cancer models.^8^ In targeted oncology, systematic optimization of the 2-aryl-4-aminoquinazoline motif has recently yielded fourth-generation non-covalent EGFR inhibitors that overcome acquired Osimertinib resistance, with enzymatic IC_50_ of 0.056 µM and tumor growth inhibition of 64.2% *in vivo*^9^ and tricyclic quinazoline-derived KRAS-SOS1 protein–protein interaction disruptors with IC_50_ of 5.1 nM and 70.5% xenograft tumor growth inhibition in pancreatic cancer models.^10^ The scaffold additionally underpins inhibitors of NSD2 and LSD1 histone-modifying enzymes^11^ and first-in-class DNMT1 molecular glue degraders in acute myeloid leukaemia.^12^

**Table 1:** Pharmacological target diversity of the quinazoline scaffold

Therapeutic area	Biological target	Representative scaffold	Key pharmacological result
Epigenetic oncology	G9a/GLP (EHMT1/2) histone methyltransferase	BIX-01294, UNC0638 family	Canonical 6,7-(OMe)_2_-4-amino quinazoline pharmacophore; PROTAC reduces G9a/GLP protein levels in MCF-7; selectivity >100-fold over other KMTs
	NSD2 (protein lysine methyltransferase)	6,7-(OMe)_2_-4-aminoquinazoline	Highly potent and selective NSD2 inhibition; potent cellular H3K36me2 reduction; anti-proliferative activity in NSD2-overexpressing cancer lines
	LSD1 (KDM1A) histone demethylase	2-Aryl-4-aminoquinazoline	LSD1 inhibition activates immune response; potent cellular activity; synergistic with PD-1/Pd-L1 inhibition
Kinase oncology (EGFR)	Del19/T790M/C797S	2-Aryl-4-amino quinazoline	Enzymatic IC_50_ = 0.056 µM *vs.* EGFR C797S; IC_50_ = 0.143 µM in resistant PC-9 cells; oral bioavailability 22.3%; TGI 64.2% *in vivo*
	KRAS-SOS1 protein–protein interaction	Tricyclic quinazoline	SOS1 IC_50_ = 5.1 nM; TGI 70.5% in pancreatic cancer xenograft; favorable oral PK
Haematological malignancy	Molecular glue degradation	First-in-class selective DNMT1 degrader	Potent and selective DNMT1 degradation; therapeutic potential in AML models; favorable selectivity over DNMT3A/3B
Antibacterial (AMR)	DHFR + Topo-IV (*S. aureus*/MRSA, *E. coli*)	2-Aryl-4-amino quinazolines	MIC = 2.2–4.4 µM *vs.* MRSA; dual DHFR + Topo-IV inhibition; low HepG2 cytotoxicity; favorable ADME + BBB permeability
Antimycobacterial (TB)	*Mycobacterium tuberculosis*	4-Amino quinazoline scaffold-hop series	MIC = 0.28 µM *vs.* multiple *M. tuberculosis* strains; confirmed intracellular macrophage model efficacy
Antiparasitic (HAT)	Human African trypanosomiasis	Lapatinib scaffold repurposing	Potency retained *vs. T. brucei*; improved aqueous solubility + metabolic stability; repurposed from EGFR inhibitor
Antiparasitic	*Trypanosoma cruzi*(dual antifolate + ROS/NO mechanism)	2-Arylquinazolin-4(3H)-one	Sub-µM activity *vs. L. infantum* promastigotes/amastigotes and *T. cruzi* epimastigotes; macrophages; non-mutagenic

Beyond oncology, this scaffold has demonstrated compelling activity against infectious disease targets of global significance. Recent 2-aryl-4-aminoquinazoline series have established dual DHFR/topoisomerase IV inhibition in methicillin-resistant *Staphylococcus aureus* with 2.2–4.4 µM MIC,^13^ while scaffold-hopping onto the 4-aminoquinazoline core has yielded compounds with 0.28 µM MIC against *Mycobacterium tuberculosis via* a novel mechanism distinct from established TB drug targets.^14^ Repurposing from EGFR kinase inhibitors has further delivered potent 4-aminoquinazoline antitrypanosomal agents against *Trypanosoma brucei*^15^ and *Leishmania infantum* with selectivity indices of 15–17.6 *versus* host macrophages.^16^ Additionally, the escalating global burden of antimicrobial resistance (AMR), responsible for an estimated 700 000 deaths annually and projected to reach ∼10 million by 2050,^17^ lends urgency to the development of pharmacologically validated scaffolds by efficient and more sustainable synthetic routes. Taking collectively, these studies establish the 6,7-dimethoxy-2-aryl-4-aminoquinazoline class as a structurally privileged, target-agnostic pharmacophoric framework of broad and growing relevance across medicinal chemistry and providing compelling justification for the development of efficient, more sustainable and scalable synthesis access to this compound class.

Conventional protocols for the two-step synthesis of this scaffold have historically depended on toxicologically problematic solvents such as 1,4-dioxane for cross-coupling and polar aprotic solvents DMF, DMAc and NMP for aromatic nucleophilic substitution (SNAr) reactions. The continued use of 1,4-dioxane is increasingly untenable in responsible pharmaceutical synthesis. Its IARC Group 2B human carcinogen classification, exceptional groundwater persistence due to resistance to aerobic biodegradation and propensity to generate explosive peroxide impurities collectively render it incompatible with modern pharmaceutical sustainability frameworks.^18^ DMF, DMAc and NMP each bear H360 reproductive toxicity designations and face progressive use restrictions under REACH Annex XVII,^18,19^ as depicted in Fig. 1b.

The global mandate to redesign pharmaceutical synthesis within the Anastas–Warner green chemistry framework^1,20^ has stimulated two strategically complementary responses. Firstly, systematic replacement of hazardous solvents with bio-derived alternatives ranked by structured solvent selection guides^21^ and secondly, adoption of microwave-assisted organic synthesis (MAOS) as an energy-efficient activation modality.^22^

Building upon a prior investigation of sequential SNAr and Suzuki construction of this quinazoline series under conventional hazardous solvents,^23^ the present work discloses a comprehensive greener redesign, as shown in Fig. 2a with (i) complete replacement of hazardous polar aprotic solvents with bio-renewable GVL in step 1 and CHEM21-compliant EtOH–H_2_O in step 2 and (ii) substitution of conductive heating with microwave irradiation throughout the reaction. Overall environmental performance is quantified at reaction-step in Fig. 2b by atom economy (AE),^24^ simple E-factor (sEF)^25^ and process mass intensity (PMI),^26^ contextualized against a comprehensive literature survey *via* a newly formulated Greener Index (GI) framework grounded in the CHEM21 and GSK^19,21^ solvent selection guides.

**Fig. 2:**
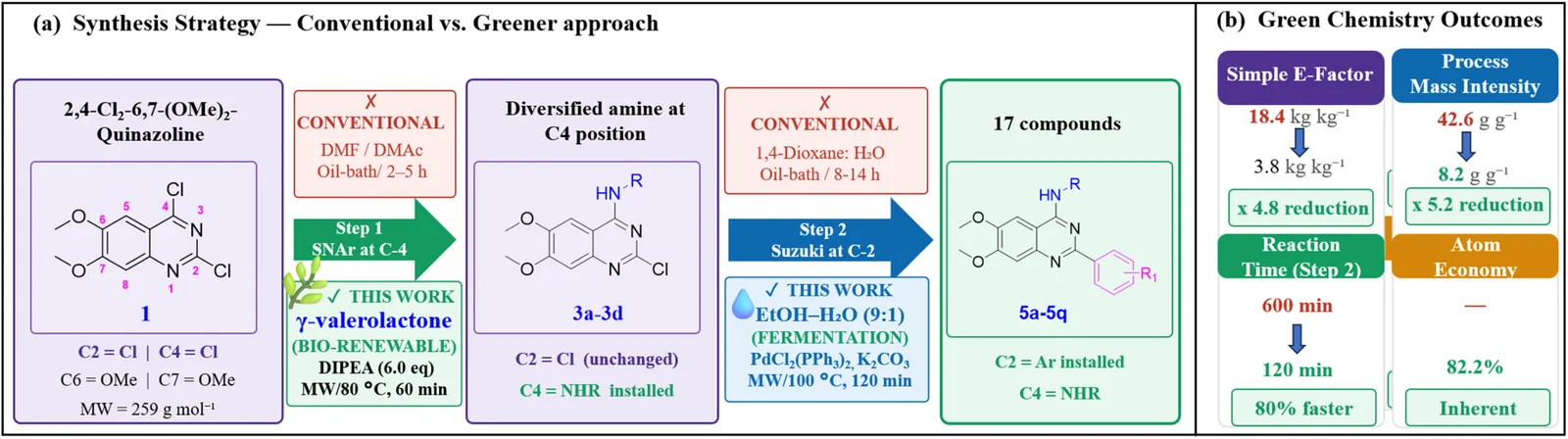
(a) Synthesis strategy; (b): greener chemistry outcomes.

## 2. Results and discussion

### 2.1 Toxicological audit of conventional solvents

A structured toxicological and environmental assessment of the conventional solvent-based protocol identified three sustainability liabilities of warranting elimination.^27^ First, 1,4-dioxane carries an IARC Group 2B carcinogen classification, exhibits exceptional groundwater persistence due to resistance to aerobic biodegradation, generates explosive peroxide impurities upon storage and has a flash point of only 12 °C. Second, DMF, DMAc and NMP each bear H360 reproductive toxicity designations under EU CLP and are subject to REACH Annex XVII restrictions. Third, these amide solvents exhibit poor biodegradability and persistent aquatic toxicity profiles, generating an occupational, regulatory and environmental burden incompatible with the sustainability targets codified by major pharmaceutical solvent frameworks.

### 2.2 Strategic design and regioselectivity rationale

The synthesis of 6,7-dimethoxy-2-aryl-4-(substituted amino) quinazolines derivatives 5a–5q require two chemically orthogonal bond-forming reactions from a common 2,4-dichloro-6,7-dimethoxyquinazoline 1 precursor as depicted in Scheme 1. The sequential strategy involves SNAr amination followed by palladium-catalyzed arylation reaction mandated by the pronounced differential electrophilicity at C-4 *versus* C-2. The regioselectivity of the first step is rationalized by frontier molecular orbital (FMO) theory and has been quantitatively confirmed by DFT calculations at the ωB97X-D/6-31G (d) level of theory with a C-PCM solvation model for polar solvents, using 6,7-dimethoxy-substituted precursor as models. Two key electronic descriptors governing the regioselectivity are LUMO coefficient distribution and the computed activation energy for the nucleophilic addition step Fig. 3.^23^

**Scheme 1:**
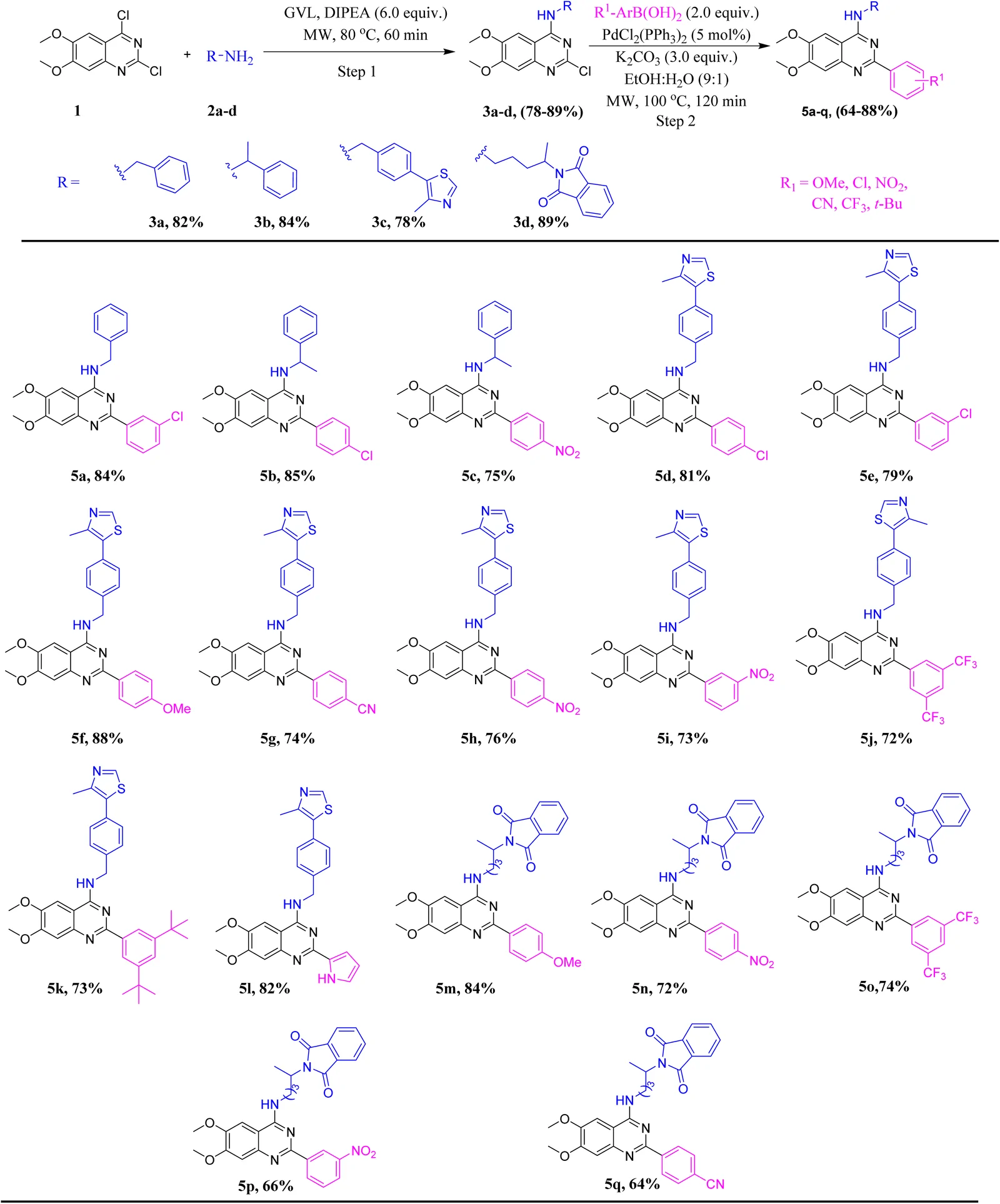
Two-step greener microwave synthesis. Step 1: 1 (1.0 equiv.), 1^o^ amine (1.2 equiv.), GVL (bio-renewable, 3.0 mL), DIPEA (6.0 equiv.), MW, 80 °C, 60 min. Step 2: 3a–3d, R_1_-ArB(OH)_2_ (2.0 equiv.), PdCl_2_(PPh_3_)_2_ (5 mol%), K_2_CO_3_ (3.0 equiv.), EtOH–H_2_O (9 : 1, 3.0 mL), MW, 100 °C, 120 min.

**Fig. 3:**
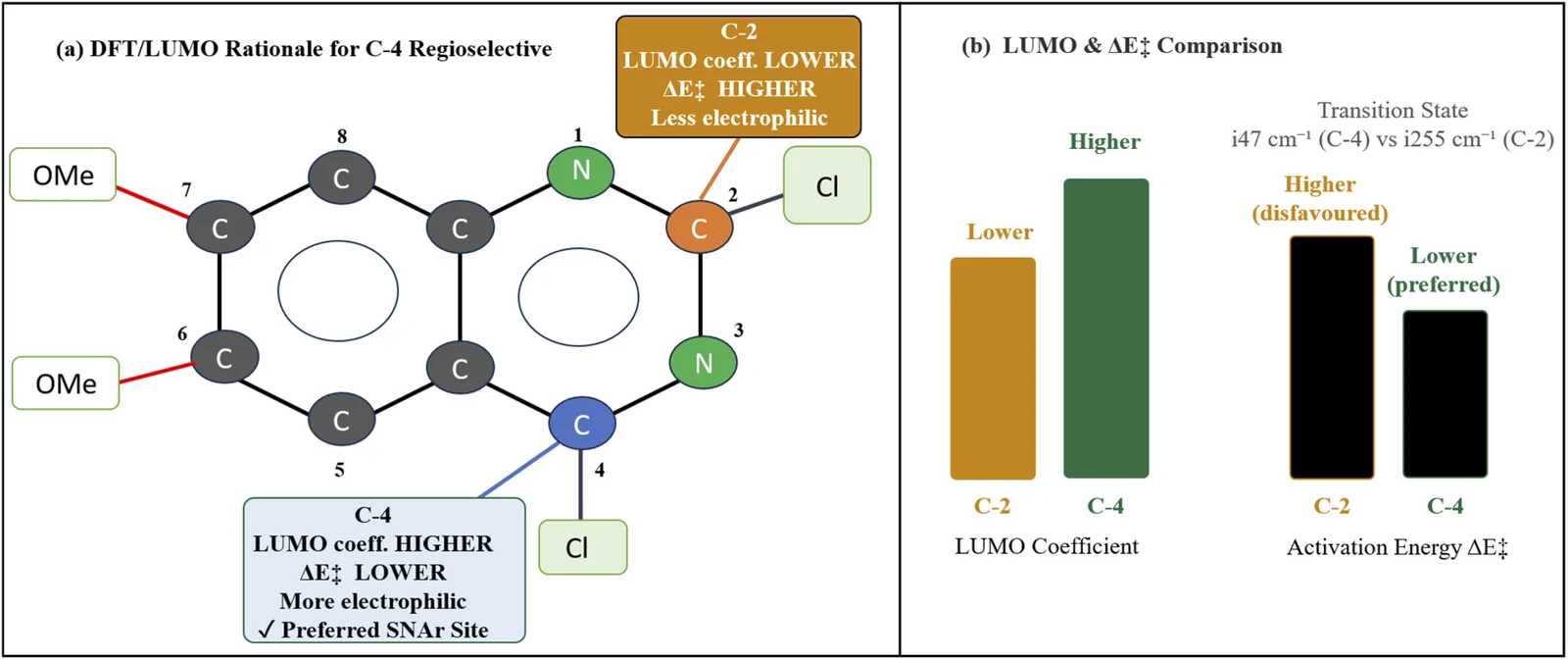
(a) DFT/LUMO Rationale for C-4 Regioselectivity; (b) LUMO & Δ*E*‡ comparison.

As shown in Fig. 3, DFT calculations reveal that C-4 bears a greater LUMO coefficient than C-2, making it inherently more susceptible to nucleophilic attack according to FMO theory. Consistent with this, the computed activation energy (Δ*E*‡) for the first step formation is lower at C-4 than at C-2. The corresponding transition states were confirmed by vibrational analysis, with imaginary frequencies of i47 cm^−1^ at C-4 and i255 cm^−1^ at C-2 respectively.

### 2.3 Chemistry

In two-step microwave-assisted conversion, the SNAr reaction of 1 with primary amines 2a–2d was carried out in bio-renewable GVL at 80 °C for 60 min using *N*,*N*-diisopropylethylamine (DIPEA, 6.0 equiv.) as a base, affording intermediates 3a–3d in 78–89% yield with complete C-4 regioselectivity. In the next step, a greener approach for the subsequent palladium-catalyzed arylation at C-2 was established, guided by a systematic literature survey.

### 2.4 Step 1 literature survey and SNAr amination at C-4 using bio renewable GVL as a solvent

A comprehensive literature survey of SNAr at C-4 of 2,4-dichloro-6,7-dimethoxy-quinazoline 1 reveals that this transformation is typically performed in polar aprotic solvents, as summarized in Table 2. Conventional polar aprotic solvents including DMF, THF, DMSO and 1,4-dioxane consistently deliver 60–96% yield but are classified as ‘Hazardous’ by CHEM21 guide with greener index (GI) of 3.0–6.0 owing to CMR toxicity designations (H360), REACH Annex XVII restrictions and poor biodegradability profiles. Among CHEM21-recommended alternatives, ethanol with GI of 7.6 has been reported as a viable medium for this transformation, affording 42–68% yield.^28^ However, the reduced yields in protic solvents are mechanistically consistent with partial hydrogen-bond quenching of amine nucleophilicity, which attenuates addition to the Meisenheimer complex.^23^

**Table 2:** Literature survey and comparison table for SNAr amination at C-4 of 2,4-dichloro-6,7-dimethoxyquinazoline 1 with primary amines^a^2a–2d

Entry	Solvent	Base (equiv.)	*T* (°C)/mode	Time	Yield (%)	CHEM21	GI
1	DMF	K_2_CO_3_ (3.0)	0 °C – rt/Conv.	2 h	90–96	Hazardous	3.2
2		DIPEA (5.0)	rt/Conv.	5 h	91–94	Hazardous	3.2
3		DIPEA (2.0)	rt-50 °C/Conv.	3 d	62–84	Hazardous	3.2
4	Tetrahydrofuran	Et_3_N (1.5)	rt/Conv.	4 h	75–88	Problematic	4.0
5		DIPEA (3.0)	rt/Conv.	12 h	75	Problematic	4.0
6	Acetonitrile	DIPEA (1.5)	80 °C/Conv.	o/n	75–85	Hazardous	4.4
7	1,4-Dioxane	DIPEA (2.2)	80 °C/Conv.	12 h	60–65	Hazardous	3.0
8	DMSO	DIPEA (1.5)	rt/Conv.	1 h	60–82	Problematic	6.0
9	EtOH	DIPEA (3.0)	rt/Conv.	12 h	42–68	Recommended	7.6
**10**	**GVL (this work)**	**DIPEA (6.0)**	**80 °C/MW**	**60 min**	**78–89**	**Recommended**	**8.2**

a GI – greener index (1–10); MW – microwave; Conv.– conventional; o/n – overnight; rt – room temperature.

Collectively, these observations highlight the need for greener process development, emphasizing solvent replacement, reduced reaction time and lower energy input while maintaining high regioselectivity at C-4. In the present work, according to GSK Solvent Sustainability Guide, bio-renewable GVL (entry 10, Table 2) with GI of 8.2 was identified as the optimal step 1 reaction medium, uniquely combining the polarity required for SNAr, as comparable to DMF with a non-CMR safety profile, ready biodegradability (OECD 301B) and absence of REACH restrictions. Under microwave irradiation at 80 °C using DIPEA (6.0 equiv.) for 60 min, intermediates 3a–3d were obtained in 78–89% yield representing a 2.6-fold improvement in composite GI over DMF. To provide a rigorous basis for solvent selection and to address the direct comparison of GVL with conventional solvents, a detailed physicochemical and sustainability comparison is provided in SI (see section of SI 2.1 for more details). The dielectric constant of GVL (*ε* = 36.5) closely mirrors that of DMF (*ε* = 36.7), providing comparable solvation capacity for the Meisenheimer complex intermediate in the SNAr mechanism. All key substrates (compound 1), amines 2a–2d dissolved completely in GVL at 80 °C within 2 min of microwave irradiation, as visually confirmed. Importantly, intermediates 3a–3d precipitated upon addition of ice-cold water during workup, allowing purification without organic extraction solvents providing an additional sustainability advantage over DMF-based protocols.

### 2.5 Step 2 literature survey and MW assisted Suzuki–Miyaura coupling at C-2

A systematic survey of published literature on palladium-catalyzed Suzuki–Miyaura cross-coupling at C-2 of 4-amino-2-chloro-6,7-dimethoxyquinazoline intermediates 3a–3d as depicted in Table 3. This data reveals that 1,4-dioxane-H_2_O is the overwhelmingly dominant reaction solvent,^29^ under both conventional thermal heating and microwave activation (entries 1–4, Table 3) affording 46–94% yield. Despite its widespread adoption, 1,4-dioxane poses significant sustainability liabilities, being classified ‘Hazardous’ with 3.0 GI owing to its IARC Group 2B carcinogen designation, exceptional groundwater persistence and explosive peroxide-forming tendency.^18^ The DME/EtOH/H_2_O ternary system (entry 5, Table 3) is similarly disqualified by CMR toxicity and REACH restrictions associated with the Dimethoxy ethane (DME) component.^18,19^ Toluene-H_2_O (entry 6, Table 3), requires prolonged conventional heating of 100 °C for 24 h and carries a hazardous CHEM21 classification with 3.8 GI due to toluene's reproductive toxicity (H361), high vapour pressure and progressive regulatory restriction. Greener ethereal alternative 2-methyl tetrahydrofuran (2-MeTHF)/H_2_O with 5.2–5.4 GI (entry 7, Table 3) offer partial improvement but remain problematic and are constrained by limited water miscibility, which restricts the extent to which aqueous boronate activation can be leveraged.^18^

**Table 3:** Literature survey and comparison table for Suzuki–Miyaura coupling at C-2 position

SN	Solvent system	Pd catalyst	*T* (°C)/mode	Time	Yield (%)	CHEM21	GI^a^
1	1,4-Dioxane/H_2_O (4 : 1)	PdCl_2_(dppf)	80 °C/Conv.	4 h	55	Hazardous	3
2	1,4-Dioxane/H_2_O (4 : 1)	PdCl_2_(PPh_3_)_2_	100 °C/Conv.	O/N	46	Hazardous	3
3	1,4-Dioxane/H_2_O (4 : 1)	PdCl_2_(dppf).DCM	100 °C/Conv.	4 h	94	Hazardous	3
4	1,4-Dioxane/H_2_O (4 : 1)	Pd(PPh_3_)_4_	125 °C/MW	30 min	46	Hazardous	3
5	DME/EtOH/H_2_O	PdCl_2_(PPh_3_)_2_	120 °C/MW	20 min	96	Hazardous	3.5
6	Toluene/H_2_O	Pd(PPh_3_)_4_	100 °C/Conv.	24 h	N/R^a^	Hazardous	3.8
7	2-MeTHF/H_2_O	Pd(PPh_3_)_4_	80 °C/MW	16 h	80–86	Problematic	5.2
**8**	**EtOH–H**_**2**_ **O (9 : 1)**	**PdCl**_**2**_ **(PPh**_**3**_ **)**_**2**_ **/K**_**2**_ **CO**_**3**_ **(3.0)**	**100 °C/MW**	**120 min**	**64–88**	**Recommended**	**8.4**

a GI – greener index; 2-MeTHF – 2-methyltetrahydrofuran; MW – microwave; O/N – overnight, Conv. – conventional, yield not reported (N/R).

This survey reveals a complete absence of any CHEM2 recommended solvent system in the literature for this transformation, a conspicuous sustainability gap that the present work directly addresses. The binary EtOH–H_2_O (9 : 1 v/v, entry 8, Table 3) system achieves CHEM21 recommended classification with 8.4 GI on sustainability, mechanistic efficacy and practical operability.^18^ Mechanistically, it is proposed that in the aqueous-protic EtOH–H_2_O environment, the base (K_2_CO_3_) generates hydroxide anions that activate the arylboronic acid to form the reactive [ArB(OH)_3_]^−^ boronate anion (‘ate complex’), facilitating the rate-determining transmetalation step for aryl chloride electrophiles. This mechanistic rationale is consistent with the established hydroxide-mediated pathway of the Suzuki–Miyaura catalytic cycle and is offered as a plausible mechanistic proposal (see SI, section 3.3, Fig. S1) to rationalize the observed efficacy of EtOH–H_2_O system; it is not directly demonstrated by the experimental data reported here.^30^ Under optimized conditions with PdCl_2_(PPh_3_)_2_ (5 mol%), K_2_CO_3_ (3.0 equiv.), 100 °C, MW for 120 min, products 5a–5q were obtained in 64–88% yield, representing a 2.8-fold GI improvement over the conventional 1,4-dioxane/H_2_O benchmark, confirming this work constitutes the first CHEM21-Recommended Suzuki protocol for this quinazoline scaffold.

### 2.6 Substrate scope and electronic effects

The optimized two-step greener microwave protocol was applied to a panel of four primary amines 2a–2d and six aryl boronic acids, delivering 6,7-dimethoxy-2-aryl-4-aminoquinazoline library 5a–5p in 64–88% overall isolated yield as depicted in Scheme 1. In step 1, the SNAr reaction proceeded at C-4 position of compound 1 with complete regioselectivity with all four amines 2a–2d and afforded compounds 3a–d in 78–89% yield. The steric accessibility of the attacking amine nitrogen governed efficiency with linear 3-(phthalimidopropyl) amine 2d gave the highest yield producing 3d in 89% owing to its unhindered primary nitrogen. Whereas the combined steric and inductive deactivating effect of the quaternary-carbon tether in 4-methylthiazolyl amine 2c gave the lowest yield of 78% for the compound 3c. However, benzylamine 2a and *α*-methylbenzylamine 2b delivered intermediate yields with 84% and 82%, consistent with their moderate benzylic nucleophilicity forming 3a and 3b respectively. The overall trend across the amine series in SNAr reaction (step 1) demonstrates that combined steric and inductive deactivation, rather than steric bulk alone, governs SNAr efficiency. The microwave activation at 80 °C employed in this work is mechanistically tailored to the comparatively attenuated nucleophilicity of primary amines as the energy input is necessary to overcome the higher activation barrier of the rate-determining nucleophilic addition step at C-4, as quantitatively supported by DFT calculations discussed in section 2.2.

In step 2, the electronic character of the aryl boronic acid exerted a pronounced and systematic influence on Suzuki coupling yield, following the trend 4-OMe > 4-Cl > 4-NO_2_ ≈ 4-CN ≈ 3,4-(CF_3_)_2_ > 4-^*t*^Bu, in close agreement with the Hammett *σ*_p_ scale of −0.27 to +0.78. The electron-donating *para*-methoxy group (4-OMe) consistently afforded 5f and 5m with highest yield of 88% and 84% by enhancing electron density at the boron center. Additionally, π-rich nature of 2- pyrrole ring in 5l resulted in 82% yield. This phenomenon is consistent with the proposed boronate activation pathway. However, the weakly electron-withdrawing *p*-chloro group did not show significant influence on Suzuki coupling efficiency as its modest inductive withdrawal was partially offset by π-donation from the halogen lone pairs in (5a, 84%; 5b, 85%; 5d, 81%; 5e, 79%). Further, strong withdrawing groups like 4-nitro (5c, 75%; 5h, 76%; 5i, 73%; 5n, 72%), 4-cyano (5g, 74%; 5q, 64%), 3-nitro (5p, 66%) and 3,4-bis(trifluoromethyl) (5j, 72%; 5o, 74%) have progressively showed decreased in the yield by reducing boronic acid nucleophilicity towards palladium. The steric bulk of 4-*tert*-butyl (5k, 73%) overrode its modest electron-donating character, impeding transmetalation approach and congesting the reductive elimination transition state. Overall, the electronic nature of the substrates significantly influences reactivity; aryl halides bearing electron-withdrawing substituents undergo more facile oxidative addition to the Pd(0) catalyst due to increased electrophilicity of the carbon–halogen bond, resulting in higher reaction rates and yields. Conversely, electron-donating substituents increase electron density on the aromatic ring and generally reduce the rate of oxidative addition resulting into reduced yield. (See SI sections 3.0 for more details on optimization of reaction parameters, mechanistic interpretation). Alkylboronic acid substrates were deliberately excluded, as alkyl-Pd(ii) intermediates formed after transmetalation are susceptible to competitive β-hydride elimination at the elevated temperature (100 °C, 120 min) employed in this work, which produces alkene by-products at the expense of the desired biaryl product. Boronic acids/esters bearing free hydroxyl, aldehyde, carboxylic acids or strongly coordinating *N*-heterocycles were also not assessed as they coordinate to the Pd(0) centre in competition with the PPh_3_ ligands, inhibiting catalyst activity. These limitations are explicitly acknowledged and identified as priorities for future extension of this methodology.

## 3. Greener index (GI) methodology and scoring matrix

To provide a reproducible and consistent numerical metric for comparing solvent sustainability across both reaction steps, we introduce the Greener Index (GI), a composite scoring framework grounded in the CHEM21 Solvent Selection Guide and the GSK Solvent Sustainability Guide. Each solvent is evaluated across five equally weighted criteria on a 1–10 scale: (*S*) Safety-fire and explosion risk assessed by flash point and autoignition temperature; (*H*) Health-acute and chronic toxicity including CMR status; (*E*) Environment-biodegradability (OECD 301B), aquatic toxicity and atmospheric persistence; (*P*) Process-reaction compatibility and catalyst stability; (*W*) Waste-recovery feasibility and contribution to sEF. The composite score is GI = (*S* + *H* + *E* + *P* + *W*)/5. A GI ≥ 7.0 is designated the ‘greener target zone’, consistent with solvents rated ‘Recommended’ by at least one major pharmaceutical solvent guide.^18^

### 3.1 Equal weighting

Equal weighting (*w* = 0.20 per criterion) was adopted on the basis of two principles: (1) methodological conservatism-in the absence of pharmacopoeially mandated priority hierarchies, equal weighting avoids arbitrary elevation of any single dimension and is consistent with the approach used by the CHEM21 Solvent Selection Guide itself, which classifies solvents holistically across safety, health and environmental axes without explicit numerical weights.^21^ (2) multi-criteria decision analysis (MCDA) convention equal weighting represents the neutral prior in analytic hierarchy process (AHP) and is standard practice when expert consensus on differential weighting is unavailable. We acknowledge that GI is designed as a comparative screening tool, not a regulatory compliance metric (Table 4).

**Table 4:** Explicit scoring rubric for each GI sub-criterion

Criterion	Score 1–3 (poor)	Score 4–6 (moderate)	Score 7–10 (good)	Primary data source
Safety (*S*)	Fp < 23 °C or explosive peroxide former	Fp 23–60 °C or moderate fire risk	Fp > 60 °C; no peroxide risk; no flammability	Flash pt (ECHA REACH); GHS/CLP
Health (*H*)	CMR, 360/H340/H350 or IARC 1/2A	Non-CMR but H361 or IARC 2B	Non-CMR; no reproductive tox.; LD_50_ > 2000 mg kg^−1^ (rat oral)	GHS SDS; ECHA C&L database
Environment (*E*)	Not readily biodeg. (OECD 301B) + high aquatic tox.	Moderately biodegradability	Readily biodeg.(OECD 301B); low aquatic tox.; low atm. Persistence	OECD 301B; ECHA REACH
Process (*P*)	Incompatible catalyst/base; low bp	Acceptable with minor caveats	High bp; no inhibitory effects	In-house exp. data; literature
Waste (*W*)	Difficult to recover; high sEF contribution	Moderate recovery; average sEF	Easy to recover; low sEF contribution; compatible waste stream	Distillation feasibility; OECD 301B

### 3.2 Individual score assignment criteria

#### 3.2.1 Sensitivity analysis

To test GI robustness to weighting assumptions, three scenarios were evaluated: Scenario 1 (equal, baseline: all *w* = 0.20); Scenario 2 (health & safety priority: *w*_*S* = 0.30, *w*_*H* = 0.25, *w*_*E* = 0.25, *w*_*P* = 0.10, *w*_*W* = 0.10); Scenario 3 (environment priority: *w*_*E* = 0.30, *w*_*H* = 0.25, *w*_*S* = 0.25, *w*_*P* = 0.10, *w*_*W* = 0.10). Results in Table 5 confirm that in all three scenarios, GVL and EtOH/H_2_O remain in the recommended zone (GI ≥ 7.0) and DMF/1,4-dioxane remain Hazardous, demonstrating that the GI conclusions are robust to weighting assumptions.

**Table 5:** Greener Index (GI) scoring matrix for key solvents in step 1 and step 2

Solvent	*S*	*H*	*E*	*P*	*W*	GI	CHEM21
DMF	3	1	3	6	3	3.2	Hazardous
1,4-Dioxane	3	2	2	5	3	3.0	Hazardous
DMSO	7	6	4	8	5	6.0	Problematic
2-MeTHF	4	6	5	6	5	5.2	Problematic
EtOH	5	8	8	9	8	7.6	Recommended
1,4-Dioxane/H_2_O	3	2	2	5	3	3.0	Hazardous
THF/H_2_O	3	5	3	5	4	4.0	Problematic
**GVL (this work-step 1)**	**8**	**8**	**8**	**7**	**7**	**8.2**	**Recommended** a
**EtOH/H**_**2**_ **O (this work-step 2)**	**6**	**9**	**9**	**9**	**9**	**8.4**	**Recommended**

a Scoring: *S* – safety; *H* – health; *E* – environment; *P* – process; *W* – waste (each 1–10). GI – (*S* + *H* + *E* + *P* + *W*)/5. Green target zone: GI ≥ 7.0. GVL classified ‘Recommended’ by GSK solvent sustainability guide (2016).

## 4. Quantitative sustainability assessment

### 4.1 Green metrics calculation and benchmarking

Three complementary and externally benchmarkable green chemistry descriptors were computed for the step 2 Suzuki–Miyaura transformation, using the model reaction of 3a (MW = 329.8 g mol^−1^) with phenylboronic acid (MW = 121.9 g mol^−1^) to afford 5a (MW = 371.4 g mol^−1^) at 0.5 mmol scale as the reference calculation. Atom economy (AE), introduced by Trost^24^ to quantify the fraction of starting material mass incorporated into the desired product was calculated as.

AE = 371.4/(329.8 + 121.9) × 100 = 82.2%.

The by-products boric acid [B(OH)_3_] and potassium chloride [KCl] are non-toxic, non-persistent, and environmentally benign. This 82.2% AE is inherent to the Suzuki mechanism and is maintained regardless of solvent system.

The performance metrics are reported here as reaction-step values, following the simple E-factor (sEF), defined by Sheldon^25^ as total waste mass divided by isolated product mass was determined experimentally as 3.8 kg kg^−1^ for microwave reaction *versus* 18.4 kg kg^−1^ for conventional, a 4.8-fold waste reduction as depicted in Fig. 4a. The dominant driver is the 62% reduction in solvent volume per reaction from 8 mL to 3 mL. The reaction-step process mass intensity (PMI) showed a 5.2-fold improvement as shown in Fig. 4b which accounts for all material inputs per gram of product^31^ and calculated as PMI of 8.2 g g^−1^ for MW *versus* reaction-step PMI of 42.6 g g^−1^ for conventional method. Purification materials (silica gel, CH_2_Cl_2_/MeOH eluent) are excluded from these reaction-step values.

**Fig. 4:**
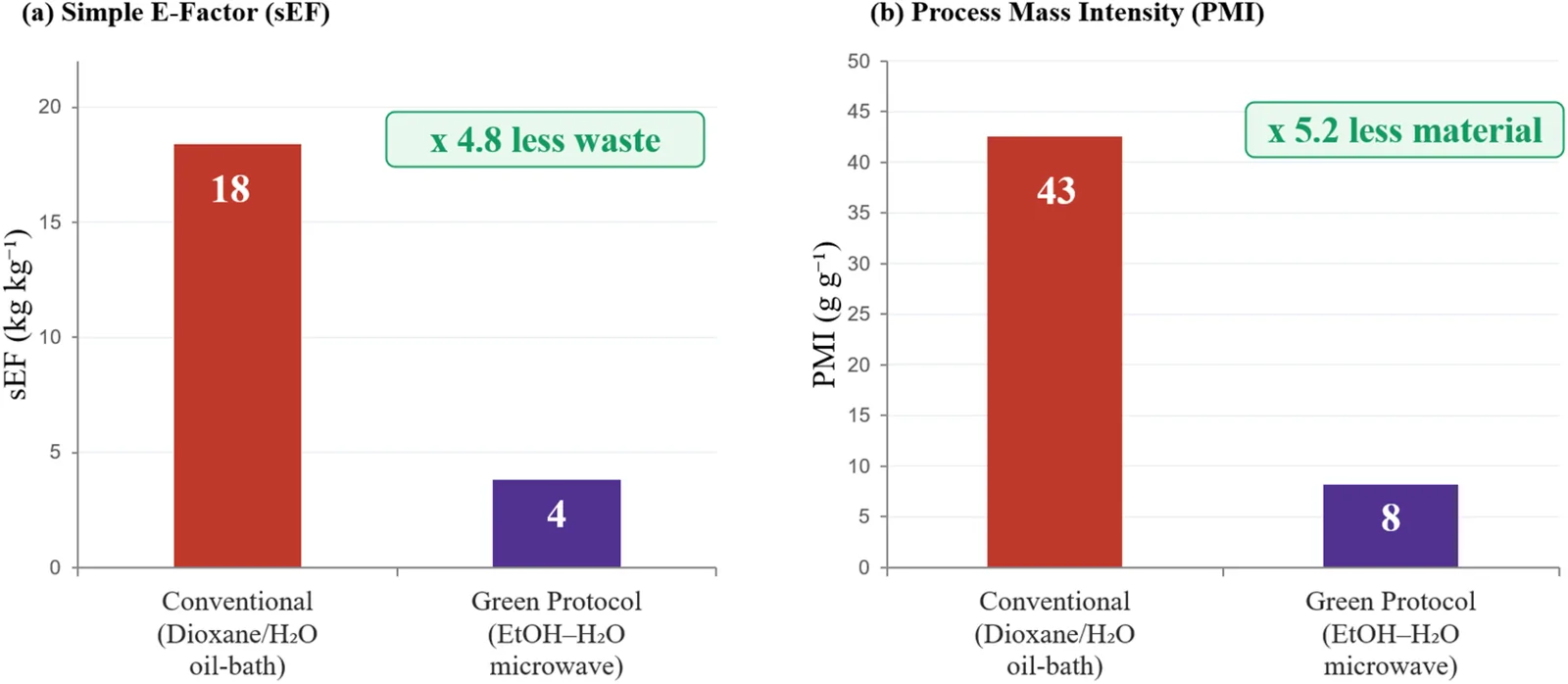
Sustainability matrix for model reaction with substrate 5a at reaction-step: (a) simple E-factor (sEF) comparison; (b): process mass intensity (PMI) comparison.

### 4.2 Alignment of results with the twelve Anastas–Warner green chemistry principles

The present two-step protocol directly addresses seven of the twelve Anastas–Warner principles as depicted in Fig. 5. Waste prevention (P1) is demonstrated by a 4.8-fold reduction in reaction-step sEF (18.4 to 3.8 kg kg^−1^) and 5.2-fold reduction in reaction-step PMI (42.6 to 8.2 g g^−1^). To our desire, Atom economy (P2) for the Suzuki stage is 82.2%, with only benign B(OH)_3_ and KCl as by-products.

**Fig. 5:**
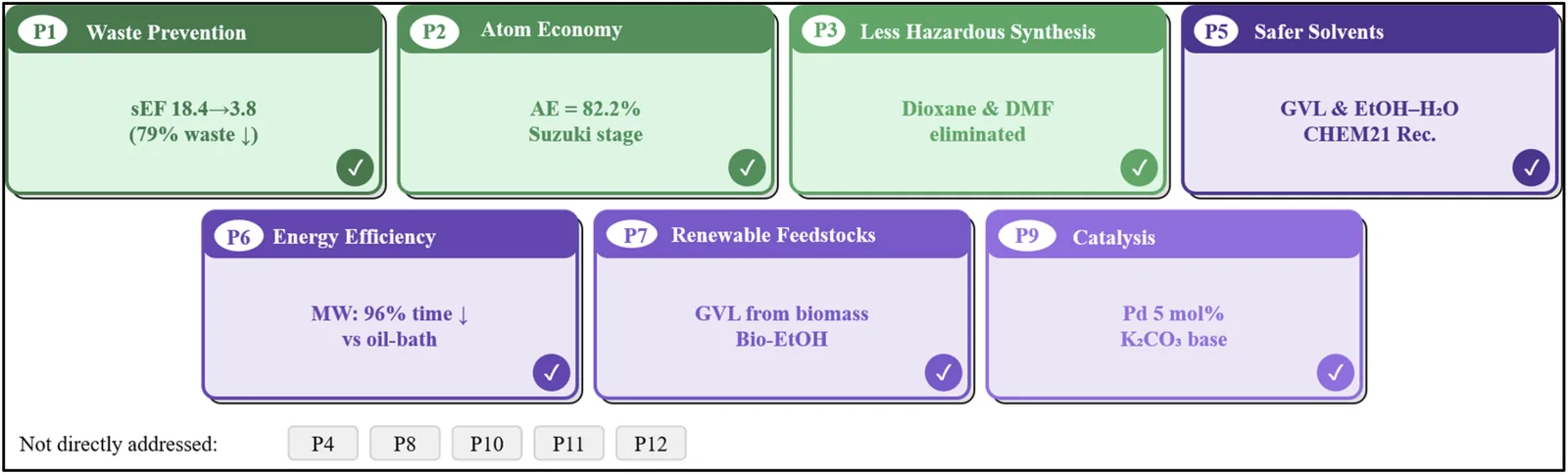
Alignment of the present work with the twelve Anastas–Warner green chemistry principles. Colored box segments (green: principles 1–3; purple: principles 5–7, 9).

Hazard elimination (P3) is realized through complete removal of 1,4-dioxane (IARC 2B) and replacement of CMR-designated DMF (H360) with non-CMR GVL. Safer solvents (P5) GVL and EtOH–H_2_O; both achieve CHEM21 recommendation with 8.2 and 8.4 GI respectively and derived from renewable, biodegradable sources (P7). Energy efficiency (P6) is demonstrated by an 80% step 2 reaction time reduction under microwave activation. Catalysis (P9) is exemplified by PdCl_2_(PPh_3_)_2_ at 5 mol% with K_2_CO_3_.

## 5. Conclusions

A sustainability derived, time-compressed two-step synthetic platform for constructing 6,7-dimethoxy-2-aryl-4-substituted aminoquinazoline libraries have been developed, quantitatively benchmarked and validated across a panel of substrate scope. The platform provides scalable, more sustainable access to a compound class with demonstrated pharmacological relevance across distinct therapeutic targeted application that justifies the urgent need for efficient and more sustainable synthetic access described herein. Two defining modifications synergistically deliver improvements across every major environmental performance indicator: (i) replacement of 1,4-dioxane and CMR-designated polar aprotic solvents with bio-renewable GVL solvent in step 1 with 8.2 GI and CHEM21-compliant EtOH–H_2_O solvent in step 2 with 8.4 GI; (ii) substitution of prolonged conductive heating with microwave irradiation throughout. For the SNAr amination step, GVL enables microwave activation to reduce reaction time by >70% across amine substrates with 78–89% yield relative to the conventional thermal reference. Furthermore, for the Suzuki–Miyaura step, a 4.8-fold reduction in reaction-step sEF with 3.8 from 18.4 kg kg^−1^, a 5.2-fold decrease in reaction-step PMI of 8.2 from 42.6 g g^−1^ and 80% reduction reaction time to 120 minute for step 2 are achieved while maintaining isolated yields of 64–88% across the panel of aryl boronic acid substrate scope. An intrinsic atom economy of 82.2% for the Suzuki stage, combined with verified alignment with seven of the twelve Anastas–Warner sustainability principles, establishes a rigorous and transferable greener chemistry framework. This work constitutes the first CHEM21-Recommended protocol for this strategically important quinazoline scaffold and provides a replicable blueprint for the sustainability-guided redesign of analogous.

## 6. Experimental section

### 6.1 General procedure A: SNAr amination at C-4

In a 30 mL glass microwave vial were added 2,4-dichloro-6,7-dimethoxyquinazoline 1 (1.0 equiv., 1.0 mmol), the appropriate primary amine 2a–d (1.2 equiv.) and *N*,*N*-diisopropylethylamine (6.0 equiv.) in 3 mL GVL. The reaction mixture was then microwaved at 80 °C for 60 minutes. Upon completion (monitored by TLC), the mixture was cooled to room temperature. The reaction mixture was then added dropwise to ice-cold water. The precipitated solid was collected by vacuum filtration and then washed with 1N HCl. The resulting solid precipitate was dried under reduced pressure to afford the desired 2-chloro-4-aminosubstituted quinazoline derivatives 3a–3d.

### 6.2 General procedure B: Suzuki–Miyaura coupling at C-2

In a 10 mL glass microwave vial were added 2-chloro-4-aminosubstituted quinazoline derivatives 3a–3d (1.0 equiv.), the appropriate arylboronic acid (2.0 equiv.), PdCl_2_(PPh_3_)_2_ (5 mol%) and K_2_CO_3_ (3.0 equiv.) in EtOH–H_2_O (3 ml, 9 : 1) under inert atmosphere. The reaction vial was heated under microwave irradiation at 100 °C for 120 minutes. After completion of the reaction, the mixture was diluted with water and extracted with ethyl acetate (2 times). The combined organic layer was washed with sat. Brine solution (100 mL), dried over sodium sulfate, filtered and concentrated under reduced pressure to afford the crude product. Crude material was purified by automated column chromatography over silica prepacked columns, eluting with 2–5% methanol in dichloromethane. Pure fractions were evaporated to yield the desired product 2-aryl-4-aminosubstituted quinazoline products 5a–5q in 64–88% yield. (For detailed spectroscopic analysis and data, see SI section 5.4).

## Author contributions

Rhushikesh C. Deokar: writing – original draft, writing-review & editing, validation, software, methodology, data curation, conceptualization. Thrilokraj: writing – original draft, validation, formal analysis, data curation. Nutan Sharma: writing – original draft, validation, formal analysis, data curation. Dhananjay Satav: validation, formal analysis, data curation. Markus Obkircher: supervision, validation. Sadaf Jamal Gilani: editing and validation. Saikat Sinha: supervision, formal analysis, conceptualization. Ramesh B. Dateer: writing-review & editing, validation, supervision, formal analysis, data curation, conceptualization.

## Conflicts of interest

The authors declare that they have no known competing financial interests or personal relationships that could have appeared to influence the work reported in this paper.

## Supplementary Material

RA-OLF-D6RA08343A-s001

## Data Availability

Additional supporting information can be found online in the supporting information (SI) section at the end of this article. Supplementary information: all synthetic procedures, spectroscopic characterization data (^1^H NMR, ^13^C NMR, mass spectroscopy), green metrics calculation worksheets and greener index scoring matrices. See DOI: https://doi.org/10.1039/d6ra08343a.
