# Supplementary material for: Greener and more sustainable access to 6,7-dimethoxy-2-aryl-4-aminoquinazolines via microwave-assisted chemo-selective synthesis in bio-renewable solvents
Source: RSC Adv. 2026 Nov 3. Online ahead of print. doi: 10.1039/d6ra08343a (PMC13729383; doi:10.1039/d6ra08343a)

Supplementary Information (SI) for RSC Advances.
This journal is © The Royal Society of Chemistry

Supporting Information

Greener and more Sustainable Access to 6,7-Dimethoxy-2-Aryl-4-Aminoquinazolines *via* Microwave-Assisted Chemo-Selective Synthesis in Bio-Renewable Solvents

Rhushikesh C. Deokar[a, b], Thrilokraj R[a], Nutan Sharma[c], Dhananjay Satav[b], Markus Obkircher[a], Sadaf Jamal Gilani[d], Saikat Sinha[a*] and Ramesh B. Dateer[b*]

[a]*Sigma Aldrich Chemicals Pvt Ltd, #12, Bommasandra-Jigani Link Road, Bangalore-560100, India* (an affiliate of *Merck KGaA, Darmstadt, Germany*).

[b]*Centre for Nano and Material Sciences, JAIN (Deemed-to-be) University, Jain Global Campus, Kanakapura road, Bangalore-562112, India.*

[c]*Department of Chemistry, School of Basic and Applied Sciences, SGT University, Gurugram-122505, Haryana, India*

[d] *Department of Pharmaceutical Sciences, College of Pharmacy, Princess Nourah bint Abdulrahman University, P.O Box 84428, Riyadh 11671, Saudi Arabia*

* *Corresponding authors: d.ramesh@jainuniversity.ac.in; saikat.sinha@merckgroup.com*

Table of Contents

1.0 Mechanistic Basis for Microwave Activation S2

2.0 Sustainability Framework and Solvent Evaluation

2.1 γ-Valerolactone (GVL): Bio-renewable medium for step 1 S2

2.2 EtOH-H₂O: Step 2 Suzuki cross-coupling S3

3.0 Optimization of Reaction Condition

3.1 Step 1 using GVL as a solvent (Table S2) S4

3.2 Step 2 Suzuki-Miyaura Coupling in EtOH-H₂O (Table S3) S5

3.3 Mechanistic Interpretation and Plausible Hypotheses S7

4.0 Scale-up Considerations and Practical Applicability

4.1 Direct Experimental Scale-up S8

4.2 Approaches for microwave Scale-up constraints S9

4.3 Reaction of green metric advantages at scale S9

5.0 Experimental Section

5.1 General considerations S9

5.2 General Procedure A: SNAr Amination at C-4 S10

5.3 General Procedure B: Suzuki-Miyaura Coupling at C-2 S10

5.4 Spectroscopic analysis S11

5.5 Spectral data for the synthesized products S21

1.0 Mechanistic Basis for Microwave Activation

Coupling of 2.45 GHz electromagnetic radiation with permanent dipole moments of polar solvent molecules generates volumetric heat deposition uniformly throughout the bulk liquid phase, bypassing the steep thermal gradients of conventional conductive heating. Microwave heating enables superheating and eliminates temperature gradients inherent to oil-bath systems. For Step 1, microwave activation at 80 °C in γ-Valerolactone (GVL) reduces reaction time 4-fold relative to conventional oil-bath conditions while maintaining complete C-4 regioselectivity. While in Step 2 Suzuki coupling translates to acceleration of reaction across all three elementary catalytic steps, with kinetic benefit for the rate-limiting trans metalation step, delivering a 30-fold reaction time compression at equivalent conversion.

2.0 Sustainability Framework and Solvent Evaluation

2.1 γ-Valerolactone (GVL): Bio-renewable medium for step 1

The bio-renewable solvent GVL selected for Step 1 is produced by catalytic hydrogenation of levulinic acid, obtained from acid-catalyzed dehydration and hydrolysis of hexoses from lignocellulosic biomass, placing it within the highest tier of renewable feedstock solvents consistent with Anastas-Warner Principle 7. Its dielectric constant (∼36) and dipole moment (∼4.2 D) closely approximate DMF, ensuring adequate solvation of the 2,4-dichloro-6,7-dimethoxy quinazoline **1** substrate during SNAr reaction. Its boiling point (207 °C) and flash point (96 °C) substantially exceed those of DMF (bp 153 °C, fp 58 °C) enabling microwave operation at elevated temperature with markedly improved thermal safety. GVL is water-miscible and is classified as 'Recommended' in the GSK Solvent Sustainability Guide. These properties collectively yield a Greener Index (GI) of 8.2, a 2.6-fold improvement over DMF (GI = 3.2). The comparative physicochemical properties of GVL verses other conventional polar solvents (DMF, DMAc and NMP) discussed in the current study are depicted in **Table S1.**

Table S1. Physicochemical, safety and sustainability comparison of GVL (this work) versus conventional polar solvents

Property	DMF	DMAc	NMP	1,4-Dioxane	GVL (This Work)
CAS No.	68-12-2	127-19-5	872-50-4	123-91-1	108-29-2
MW (g mol⁻^{1})	73.09	87.12	99.13	88.11	100.12
Boiling Point (°C)	153	165	202	101	207
Flash Point (°C)	58	70	91	12	96
Dielectric Constant	37.6	37.8	32.0	2.2	~36
Water Miscibility	Yes	Yes	Yes	Yes	Yes
CMR Status	H360 (RT)	H360 (RT)	H360 (RT)	IARC 2B	None
Bio-Renewable Source	No	No	No	No	Levulinic acid (biomass)
CHEM21/GSK Ranking	Hazardous	Hazardous	Hazardous	Hazardous	Recommendedᵃ
Greener Index (GI/10)	3.2	3.4	4.0	3.0	8.2

ᵃGVL is not in the original 51-solvent CHEM21 panel but is classified 'Recommended' in the GSK Solvent Sustainability Guide (2016); RT = reprotoxic.

2.2 EtOH-H₂O reaction medium for Step 2 Suzuki cross-coupling

The binary EtOH-H₂O system with 9:1 v/v was efficiently utilized for Step 2 on three independently compelling grounds. First, both components independently achieve the highest 'Recommended' tier in both the CHEM21 and GSK pharmaceutical solvent selection guides. Second, the ratio optimizes microwave energy absorption while maintaining adequate organic co-solvent content to dissolve the lipophilic intermediate and aryl boronic acid substrates; this specific ratio has direct literature precedent for quinazoline Suzuki coupling under microwave activation.

Third, bio-fermentative ethanol carries a substantially lower life-cycle carbon footprint than petrochemical solvents, aligning with Anastas-Warner Principle.

3.0 Optimization of Reaction Condition

3.1 Step 1 Using GVL as a Solvent

Bio-renewable GVL reaction medium was selected based on GI analysis and its demonstrated solvation capacity. The systematic condition optimization was conducted varying microwave temperature, irradiation time and varying bases as depicted in **Table S2**. The raw materials **2a-2d** were procured from the Sigma-Aldrich catalogues. The condition optimization for the chemoselective SNAr amination at C-4 of 2,4-dichloro-6,7-dimethoxyquinazoline **1** was conducted using benzylamine **2a** as the model primary amine nucleophile in γ-valerolactone (GVL) as the bio-renewable reaction medium under sealed-vessel microwave irradiation. In the initial experiment (**Opt.1**), the reaction was carried out in neat GVL at 60 °C for 30 min in the absence of any external base, affording intermediate **3a** in only 15% yield. This modest conversion reflects the need for an HCl scavenger to drive the equilibrium toward product formation; without base, the liberated HCl protonates the product amine, inhibiting further reaction. Introduction of triethylamine (TEA, 2.0 equiv.) improved the yield to 46% under identical conditions (**Opt.2**), confirming that proton scavenging is essential. Substitution of TEA with the bulkier, more hindered *N, N*-diisopropylethylamine (DIPEA, 2.0 equiv.) at a slightly elevated temperature (70 °C, 45 min) delivered **3a** in 65% yield (**Opt.3**), attributable to the superior non-nucleophilic character of DIPEA, which eliminates competitive *N*-alkylation of the base. A direct comparison with inorganic K_2CO_3 (3.0 equiv., **Opt.4**) afforded a comparable yield of 68%, but the heterogeneous nature of the inorganic base in GVL made DIPEA the preferred choice for homogeneous reaction control. Stepwise increase in DIPEA loading from 2.0 to 4.0 equiv. (**Opt.5**, 76%) and subsequently to 6.0 equiv. with extended irradiation time (**Opt.6**, 70 °C, 60 min, 82%) demonstrated a clear positive correlation between base stoichiometry and yield.

Table S2. Step 1 condition optimization and substrate scope using MW (SNAr amination) in GVL

Entry	R-NH₂ (1.2 equiv.)	Conditions	T (°C)	t (min)	Product	Yieldᵇ (%)
Opt-1	Benzylamine	GVL	60	30	**3a**	15
Opt-2	Benzylamine	GVL, TEA (2.0 eq)	60	30	**3a**	46
Opt-3	Benzylamine	GVL, DIPEA (2.0 eq)	70	45	**3a**	65
Opt-4	Benzylamine	GVL, K₂CO₃ (3.0 eq)	70	45	**3a**	68
Opt-5	Benzylamine	GVL, DIPEA (4.0 eq)	70	45	**3a**	76
Opt-6	Benzylamine	GVL, DIPEA (6.0 eq)	70	60	**3a**	82
**Opt-7***	**Benzylamine**	**GVL, DIPEA (6.0 eq)**	**80**	**60**	**3a**	**82**
8	**2b**	GVL, DIPEA (6.0 eq)	80	60	**3b**	84
9	**2c**	GVL, DIPEA (6.0 eq)	80	60	**3c**	78
10	**2d**	GVL, DIPEA (6.0 eq)	80	60	**3d**	89

[a]Compound **1** (1.0 mmol), amine (1.2 equiv.), DIPEA (6.0 eq), GVL (3.0 mL), MW, ᵇIsolated yield after filtration or silica gel chromatography. *= Optimized reaction condition.

The optimized conditions were identified using DIPEA (6.0 equiv., **Opt. 7***), 80 °C, 60 min, which delivered **3a** in 82% isolated yield with complete C-4 regioselectivity confirmed by ^{1}H NMR. The elevated temperature 80 °C vs. 70 °C accelerates the rate of SNAr addition to the Meisenheimer intermediate without inducing bis-amination or other side reactions, as monitored by TLC. The 6.0 equiv. DIPEA loading ensures quantitative in situ scavenging of the hydrogen chloride generated during the elimination step, maintaining the reaction driving force throughout the 60 min irradiation window.

3.2 Step 2 Suzuki-Miyaura Coupling in EtOH-H₂O (Table S3)

In step 2, reaction condition optimization for the palladium-catalyzed Suzuki–Miyaura cross-coupling at C-2 of intermediate **3a** with phenylboronic acid was performed under microwave activation in EtOH-H₂O binary medium, systematically varying the palladium catalyst, base, solvent ratio, temperature and irradiation time as depicted in **Table S3**. Initial screening with 10 mol% of Pd(PPh₃)₄ and K₂CO₃ (2.0 equiv.) in EtOH/H₂O (8:2) at 80 °C for 60 min (**Opt-1**) delivered the target product **5a** in a modest 52% yield, establishing a workable baseline but indicating significant scope for optimization. Substitution of Pd(PPh₃)₄ with the less electron-rich Pd(dba)₂ (**Opt-2**) under identical conditions gave a lower yield of 43%, reflecting the poorer performance of this catalyst in protic aqueous-ethanolic media without the stabilizing triphenylphosphine ligand. Switching to the in situ-generated Pd(OAc)₂/PPh₃ system with the 9:1 solvent ratio (**Opt-3**, 80 °C, 60 min) gave only 38% yield, the lowest in the series, consistent with the slower pre-catalyst activation kinetics of this mixture under the employed conditions.

Table S3. Step 1 Condition optimization for Step 2 microwave Suzuki-Miyaura coupling

Entry	Pd Catalyst	Base	Solvent (v/v)	T (°C)	t (min)	Yield[a] (%)
Opt-1	Pd(PPh₃)₄; 10 mol%	K₂CO₃; 2.0 equiv.	EtOH/H₂O (8:2)	80	60	52
Opt-2	Pd(dba)$_2$; 10 mol%	K₂CO₃; 2.0 equiv.	EtOH/H₂O (8:2)	80	60	43
Opt-3	Pd(OAc)₂/PPh₃; 10 mol%	K₂CO₃; 2.0 equiv.	EtOH/H₂O (9:1)	80	60	38
Opt-4	Pd(PPh₃)₄; 10 mol%	Cs₂CO₃; 3.0 equiv.	EtOH/H₂O (9:1)	100	90	63
Opt-5	PdCl₂(PPh₃)₂; 10 mol%	Cs₂CO₃; 3.0 equiv.	EtOH/H₂O (9:1)	100	100	73
Opt-6	PdCl₂(PPh₃)₂; 5 mol%	K₂CO₃; 3.0 equiv.	EtOH/H₂O	100	100	70

			(9:1)			
**Opt-7***	**$PdCl_2(PPh_3)_2$; 5 mol%**	**K_2CO_3; 3.0 equiv.**	**EtOH/H_2O (9:1)**	**100**	**120**	**84**

*Other parameters: Substrate **3a** (1.0 equiv.), 3-Cl-PhB$(OH)_2$ (2.0 equiv.), base (3.0 equiv.), solvent (3.0 mL), sealed MW vial, N_2, [a]Isolated yield.

Elevation of temperature to 100 °C and replacement of K_2CO_3 with the stronger, more soluble Cs_2CO_3 (3.0 equiv.) with $Pd(PPh_3)_4$ in EtOH/H_2O (9:1, **Opt-4**, 90 min) produced a substantially improved yield of 63%, demonstrating that both a more basic inorganic base and the higher temperature are beneficial for this aryl substrate. Replacing $Pd(PPh_3)_4$ with $PdCl_2(PPh_3)_2$ (10 mol%) under the same conditions (**Opt-5**, Cs_2CO_3, 100 °C, 100 min) further increased the yield to 73%, confirming that the pre-formed palladium(II) dichloride complex generates the active Pd(0) species more efficiently under these microwave-superheated conditions than the phosphine-free Pd(0) precursor. Reduction of the $PdCl_2(PPh_3)_2$ catalyst loading from 10 to 5 mol% while reverting to the operationally simpler and more sustainable K_2CO_3 base (3.0 equiv., **Opt-6**, 100 °C, 100 min) delivered 70% yield, demonstrating that catalyst economy is achievable without significant yield penalty when using K_2CO_3 in place of Cs_2CO_3.

The optimal conditions (**Opt.7***) were identified with 5 mol% of $PdCl_2(PPh_3)_2$, K_2CO_3 (3.0 equiv.) in EtOH/H_2O (9:1 v/v) at 100 °C for 120 min under microwave irradiation, affording product **5a** in 84% isolated yield. The extended irradiation time of 120 min relative to **Opt-6** (100 min) provided the final increment in conversion, confirming that complete transmetalation of the aryl boronate electrophile requires an adequate irradiation window under the superheating conditions of sealed-vessel microwave activation. The combination of the CHEM21-Recommended EtOH-H_2O medium (GI = 8.4) with the low Pd loading (5 mol%) and benign inorganic base K_2CO_3 represents the optimal balance between yield, catalyst economy and sustainability.

3.3 Mechanistic Interpretation and Plausible hypotheses

The proposed mechanism is based on the well-established Suzuki-Miyaura catalytic cycle reported in the literature and is intended to provide a plausible explanation for the observed experimental. Microwave irradiation is not considered to alter the fundamental catalytic mechanism; instead, it enhances reaction efficiency through rapid dielectric heating, improved energy transfer, accelerated catalyst activation, and enhanced mass transport, thereby facilitating oxidative addition, transmetalation, and reductive elimination steps. Furthermore, the superior performance observed in polar solvent systems is attributed to improved solubilization of the inorganic base and boronic acid, stabilization of catalytic intermediates, and promotion of reactive boronate species that facilitate the transmetalation process. The plausible mechanism is depicted in Fig S1.

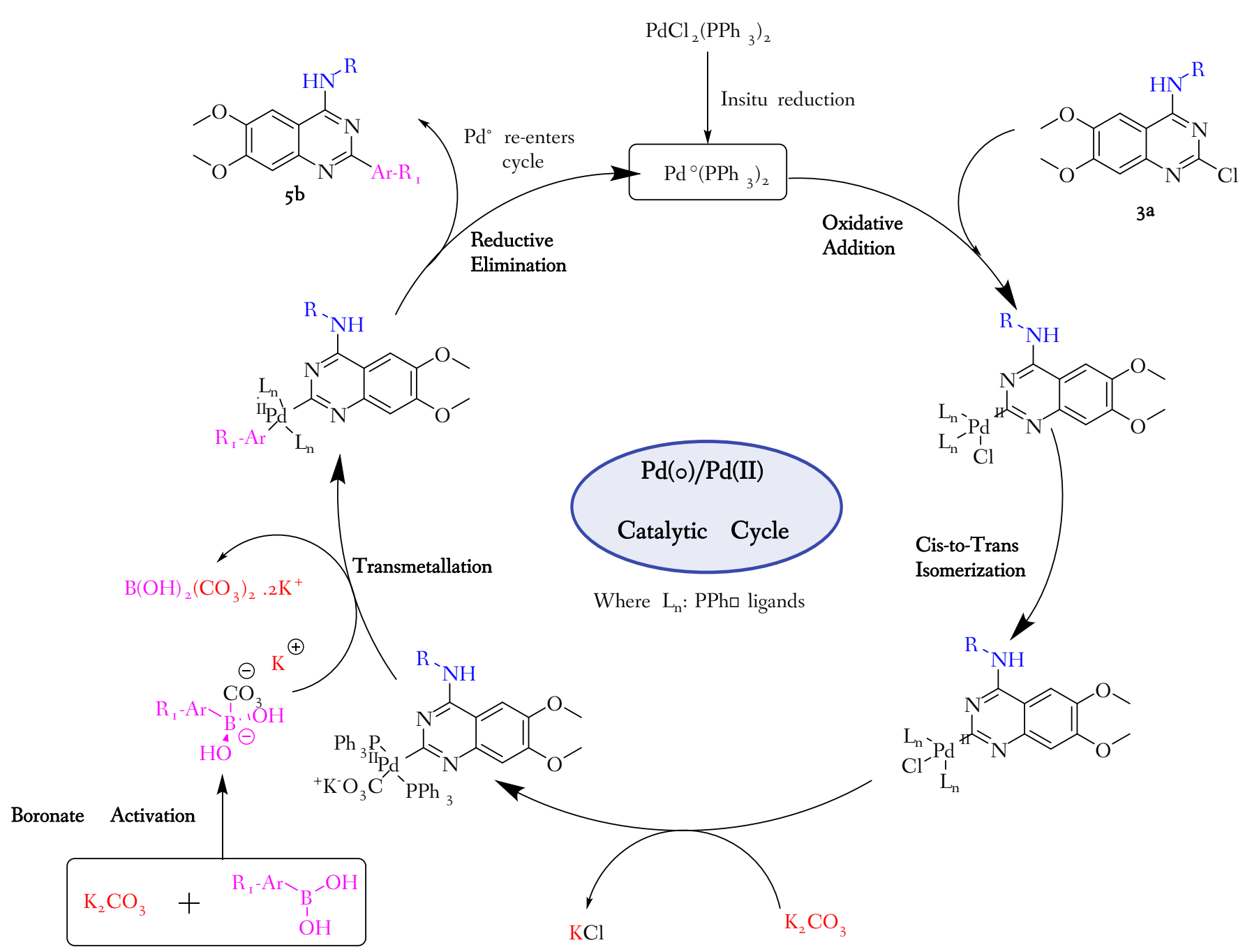

Fig S1: Plausible mechanism for Suzuki cross coupling using Pd Catalyst

The electronic nature of the substrates also significantly influences reactivity; aryl halides bearing electron-withdrawing substituents undergo more facile oxidative addition to the Pd(0) catalyst due to increased electrophilicity of the carbon-halogen bond, resulting in higher reaction rates and yields. Conversely, electron-donating substituents increase electron density on the aromatic ring and generally reduce the rate of oxidative addition.

4.0 Reaction Scalability and Practical Applicability

4.1 Direct Experimental Scale-up

The reactions described in this work were performed at 0.5–1.0 mmol laboratory scale using single-mode (monomode) microwave reactors (10 mL glass vials). This scale is appropriate for proof-of-concept sustainability benchmarking and compound library synthesis. To substantiate the scalability claim directly, we have conducted Step 2 reaction at 5 mmol scale in a 30 mL glass microwave vial with model substrate **3a** and phenylboronic acid to afford **5a** under identical conditions (PdCl₂(PPh₃)₂, 5 mol%; K₂CO₃, 3.0 equiv.; EtOH-H₂O, 9:1; 100 °C, 120 min). Product **5a** was isolated in 82.7% yield, comparable to 84% at 0.5 mmol scale, confirming that the protocol transfers directly to a 10-fold scale increase within the monomode microwave format without loss of yield. Additionally, the terms 'scalable' in this manuscript should be understood with the green solvent advantages (GI = 8.2/8.4 for GVL/EtOH–H₂O) and are thermodynamic and regulatory properties of the solvent systems themselves, fully retained at any scale.

4.2 Approaches for Microwave Scale-up Constraints

The principal heat-transfer constraint of single-mode microwave reactors is the electromagnetic penetration depth, which limits uniform volumetric heating to vessel diameters and precludes straightforward batch scale-up beyond ~30 mL. Following three technically validated routes address this constraint as-

Strategy A with numbered-up batch: Autosampler-equipped monomode reactors allow quasi-continuous scale-up at 3 mmol per vial with identical conditions. **Strategy B with Continuous-flow microwave (CF-MW):** CF-MW reactors circumvent the penetration-depth limit by exposing a continuously replenished thin liquid film or plug-flow stream to microwave irradiation, enabling gram-to-multigram throughput. EtOH-H₂O is particularly well-suited due to its aqueous miscibility and low viscosity. Suzuki-Miyaura couplings have been successfully translated from millimolar batch to CF-MW without loss of yield or selectivity. **Strategy C with Conventional thermal heating**: For Pd-catalyzed cross-couplings in polar solvents (EtOH-H₂O), where reaction outcomes are governed by bulk thermal effects, microwave temperature and time profiles translate directly to conventional sealed-vessel heating without loss of yield or

selectivity. This route sacrifices 80% reaction time reduction (Principle 6) but fully retains the solvent-system green advantages (GI = 8.4; elimination of hazardous 1,4-dioxane).

4.3 Retention of Green Metric Advantages at Scale

The two principal sEF/PMI drivers, the 62% reaction solvent volume reduction (8 mL to 3 mL) and elimination of IARC 2B-classified 1,4-dioxane with intrinsic properties of the EtOH-H₂O system and are fully maintained across all three pathways at any scale. The 80% reaction time reduction (Principle 6) is preserved in Pathways 1 and 2; Pathway 3 restores longer reaction times but retains all solvent-derived green advantages

5.0 Experimental Section

5.1 General Information

The starting materials, solvents, γ-Valerolactone (GVL, ≥99%, CAS 108-29-2) and reagents have been purchased from Sigma-Aldrich and Merck. The experimental techniques employed in this study included thin layer chromatography using Merck silica F254 0.255 mm plates, with visualization of spots by UV at 254 nm. Flash column chromatography was performed using Merck 60 (70-230 mesh) silica gel. A Buchi rotary evaporator was used to extract the solvents at low pressure. All reactions were carried out using Microwave reactor from Anton Paar (Monowave-300, 2.45 GHz, 300 W maximum power) in sealed borosilicate glass vials (10 mL, and 30 mL vials). The automated flash chromatographic instrument was used for purification. All 1H-NMR spectra were recorded on a Bruker-400 ultra-shield NMR spectrometer, using CD_3OD, $CDCl_3$ or DMSO-d6 as solvent and are reported in ppm referenced as an internal standard. [δ 7.26 ($CDCl_3$); 2.50 (DMSO-d6); 3.31 (CD_3OD); TMS: 0.00 ppm], multiplicity (s = singlet, d = doublet, t = triplet, q = quartet, m = multiplet, dd = doublet of doublets, brs = broad singlet), integration, and coupling constant (*J*) in hertz (Hz). A Shimadzu LCMS 2010 spectrometer (Shimadzu, Tokyo, Japan) was used to scan the mass spectra.

5.2 General Procedure A: SNAr Amination at C-4

In a 30 mL glass microwave vial were added 2,4-dichloro-6,7-dimethoxyquinazoline **1** (1.0 equiv., 1.0 mmol), the appropriate primary amine **2a-d** (1.2 equiv.) and *N, N*-diisopropylethylamine (6.0 equiv.) in 3mL GVL. The reaction mixture was then microwaved at 80 °C for 60 minutes. Upon completion (monitored by TLC), the mixture was cooled to room temperature. The reaction mixture was then added dropwise to ice-cold water. The precipitated solid was collected by vacuum filtration and then washed with 1N HCl. The resulting solid precipitate was dried under reduced pressure to afford the desired 2-chloro-4-aminosubstituted quinazoline derivatives **3a-3d**.

5.3 General Procedure B: Suzuki-Miyaura Coupling at C-2

In a 10 mL glass microwave vial were added 2-chloro-4-aminosubstituted quinazoline derivatives **3a-3d** (1.0 equiv.), the appropriate arylboronic acid (2.0 equiv.), $PdCl_2(PPh_3)_2$ (5 mol%) and K_2CO_3 (3.0 equiv.) in EtOH-H_2O (3ml, 9:1) under inert atmosphere. The reaction vial was heated under microwave irradiation at 100 °C for 120 minutes. After completion of the reaction, the mixture was diluted with water and extracted with ethyl acetate (2 times). The combined organic layer was washed with sat. brine solution (100 mL), dried over sodium sulfate, filtered and concentrated under reduced pressure to afford the crude product. Crude material was purified by automated column chromatography over silica prepacked columns, eluting with 2-5% methanol in dichloromethane. Pure fractions were evaporated to yield the desired product 2-aryl-4-aminosubstituted quinazoline products **5a–5q** in 64-88% yield.

5.4 Spectroscopic analysis for the synthesized derivatives

_N_-benzyl-2-chloro-6,7-dimethoxyquinazolin-4-amine (3a)

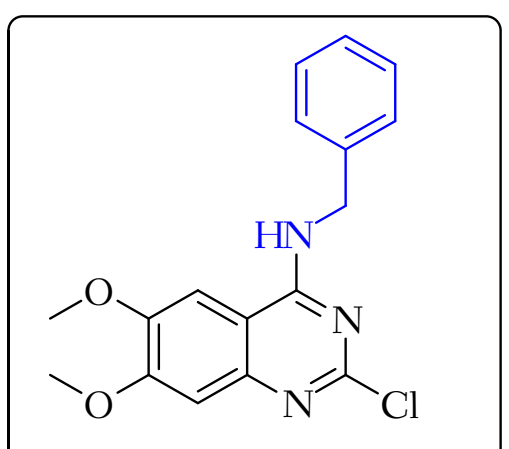

3a

Compound **3a** was prepared according to general procedure A using compound **1** and benzyl amine **2a** as a white solid (1.04 g, 82%); Rf = 0.63 (5% methanol in dichloromethane); ^{1}H NMR (400 MHz, DMSO-d$_6$): δ 9.19 (t, *J* = 6.0 Hz, 1H), 7.76 (s, 1H), 7.40 - 7.33 (m, 4H), 7.27 (t, *J* = 8.0 Hz, 1H), 7.10 (s, 1H), 4.75 (d, *J*

= 4.0 Hz, 2H), 3.90 (s, 3H), 3.89 (s, 3H). (ESI-MS) m/z: calcd. for $C_{17}H_{16}ClN_3O_2$: 329.78; found: 330.35 $[M^{+1}]$

2-chloro-6,7-dimethoxy-*N*-(1-phenylethyl)quinazolin-4-amine (3b)

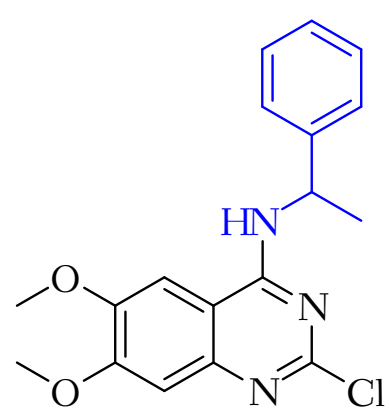

3b

Compound **3b** was prepared according to general procedure A using compound **1** and 1-phenylethan-1-amine **2b** as a white solid (1.11 g, 84%); Rf = 0.62 (5% methanol in dichloromethane); ^{1}H NMR (400 MHz, DMSO-d_6): δ 9.26 (d, *J* = 8.0 Hz, 1H), 8.02 (s, 1H), 7.48 (d, *J* = 8.0 Hz, 2H), 7.35 (t, *J* = 8.0 Hz, 2H), 7.26 (d, *J* = 8.0 Hz, 1H), 7.13 (s, 1H), 5.61 - 5.54 (m, 1H), 3.95 (s, 3H), 3.90 (s, 3H), 1.65 (d, *J* = 8.0 Hz, 3H). ^{13}C NMR (100 MHz, DMSO-d_6) δ 159.4, 155.4, 153.6, 149.3, 144.9, 128.8, 127.4, 126.8, 106.9, 105.0, 103.6, 57.1, 56.5, 50.44, 22.2. (ESI-MS) m/z: calcd. for $C_{18}H_{18}ClN_3O_2$: 343.81; found: 345.15 $[M^{+1}]$

2-chloro-6,7-dimethoxy-*N*-(4-(4-methylthiazol-5-yl)benzyl)quinazolin-4-amine (3c)

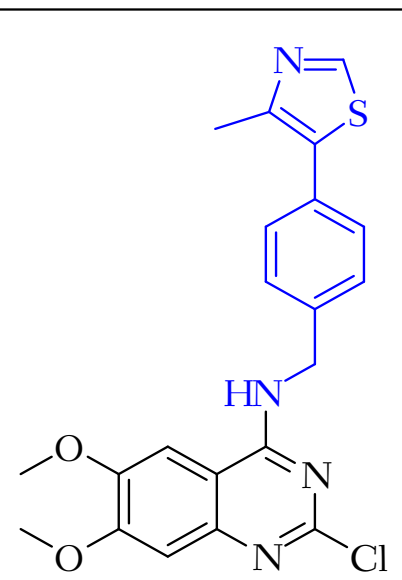

3c

Compound **3c** was prepared according to general procedure A using compound **1** and (4-(4-methylthiazol-5-yl)phenyl)methanamine **2c** as a white solid (1.29 g, 78%); Rf = 0.37 (5% methanol in dichloromethane); ^{1}H NMR (400 MHz, DMSO-d_6): 8.99 (s, 1H), 8.92 (t, *J* = 4.0 Hz, 1H), 7.70 (s, 1H), 7.49 (s, 4H), 7.11 (s, 1H), 4.79 (d, *J* = 4.0 Hz, 2H), 3.90 (s, 3H), 3.89 (s, 3H), 2.45 (s, 3H). ^{13}C NMR (100 MHz, DMSO-d_6) δ 160.5, 155.5, 155.0, 152.0, 149.1, 148.35, 147.8, 139.3, 131.5, 130.7, 129.5, 128.6, 107.3, 107.0, 102.7, 56.5, 56.3, 44.2, 16.4. (ESI-MS) m/z: calcd. for $C_{21}H_{19}ClN_4O_2S$: 426.92; found: 428.20$[M^{+1}]$

2-(5-((2-chloro-6,7-dimethoxyquinazolin-4-yl)amino)pentan-2-yl)isoindoline-1,3-dione (3d)

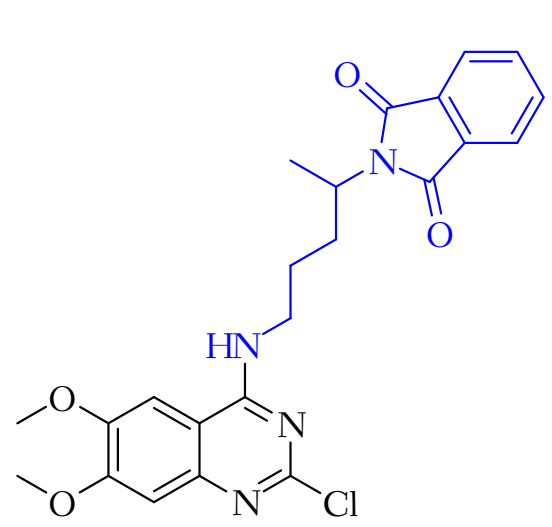

3d

Compound **3d** was prepared according to general procedure A using compound **1** and (4-(4-methylthiazol-5-yl)phenyl)methanamine **2d** as a white solid (1.56 g, 89%); Rf = 0.39 (5% methanol in dichloromethane); ^{1}H NMR (400 MHz, DMSO-d_6): δ 8.99 (brs, 1H), 7.82 (s, 4H), 7.73 (s, 2H), 7.09 (s, 1H), 4.34 - 4.30 (m, 4H), 3.89 (s, 3H), 3.87 (s, 3H), 3.51 - 3.48 (m, 2H), 2.13 - 2.03 (m, 1H), 1.82 - 1.75 (m, 1H), 1.63 - 1.54 (m, 2H), 1.41 (d, *J* = 8.0 Hz, 3H). ^{13}C NMR (100 MHz, DMSO-d_6) δ 168.5, 160.1, 155.2, 149.2, 134.9, 131.8, 123.4, 106.9, 104.8, 103.4, 56.8, 56.4, 46.9, 30.9, 26.0, 19.0. (ESI-MS) m/z: calcd. for $C_{23}H_{23}ClN_4O_4$: 454.91; found: 456.10 [M^{+1}]

_N_-benzyl-2-(3-chlorophenyl)-6,7-dimethoxyquinazolin-4-amine (5a)

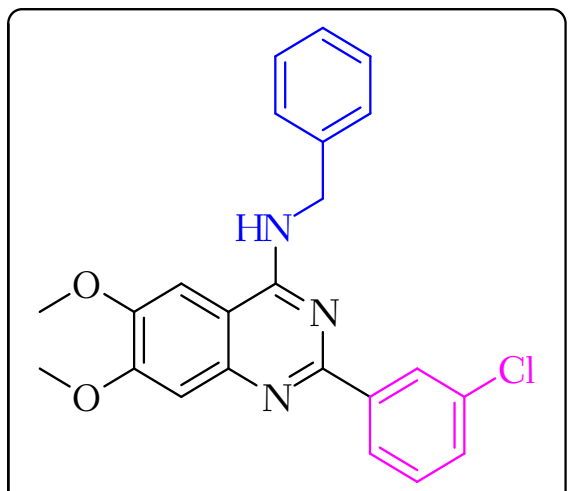

5a

Compound **5a** was prepared according to general procedure B using compound **3a** (0.2 g, 1.0 equiv.) and 3-chloro-phenylboronic acid as an off white solid (0.206 g, 84%); Rf = 0.43 (5% methanol in dichloromethane); ^{1}H NMR (400 MHz, DMSO-d_6): δ 8.67 (t, *J* = 6.0 Hz, 1H), 8.38 - 8.34 (m, 2H), 7.72 (s, 1H), 7.50 - 7.46 (m, 4H), 7.35 (t, *J* = 6.0 Hz, 1H), 7.26 - 7.23 (m, 2H), 4.89 (d, *J* = 4.0 Hz, 2H), 3.94 (s, 3H), 3.92 (s, 3H). ^{13}C NMR (100 MHz, DMSO-d_6): δ 158.9, 156.8, 154.5, 149.1, 147.0, 141.6, 140.6, 133.6, 130.6, 129.7, 128.8, 127.9, 127.6, 127.3, 126.4, 107.9, 102.5, 56.4, 56.2, 44.4. (ESI-MS) m/z: calcd. for $C_{24}H_{22}ClN_3O_2$: 405.88; found: 406.80 [M^{+1}]

2-(4-chlorophenyl)-6,7-dimethoxy-_N_-(1-phenylethyl)quinazolin-4-amine (5b)

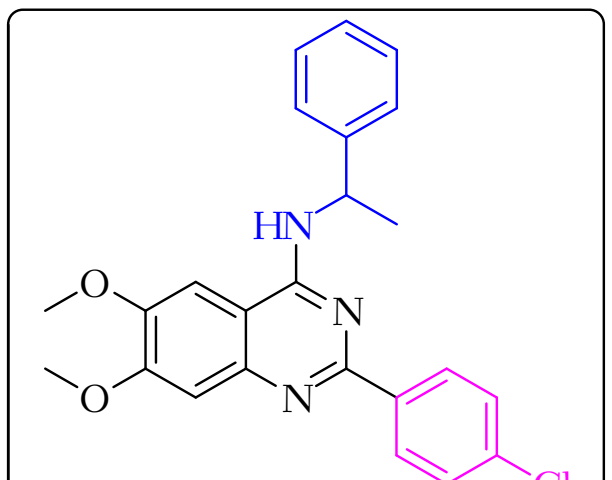

5b

Compound **5b** was prepared according to general procedure B using compound **3b** (0.2 g, 1.0 equiv.) and 4-chloro-phenylboronic acid as an off white solid (0.208 g, 85%); Rf = 0.43 (5% methanol in dichloromethane); ^{1}H NMR (400 MHz, DMSO-d_6): δ 8.32 - 8.29 (m, 3H), 7.83 (s, 1H), 7.50 (dd, *J* = 18.0, 6.0 Hz, 4H), 7.33 (t, *J* = 8.0 Hz, 2H), 5.65 (t, *J* = 8.0 Hz, 1H), 3.97 (s, 3H), 3.93 (s, 3H), 1.66 (d, *J* = 8.0 Hz, 3H). ^{13}C NMR (100 MHz, DMSO-d_6): δ 158.2, 156.6, 154.5, 149.1, 147.2, 141.6, 133.6, 130.5, 129.7, 128.8, 127.6, 126.9, 126.5, 126.4, 107.9, 102.7, 56.7, 56.2, 50.5, 23.1. (ESI-MS) m/z: calcd. for $C_{23}H_{20}ClN_3O_2$: 419.91; found: 421.75[M^{+1}]

2-(4-nitrophenyl)-6,7-dimethoxy-*N*-(1-phenylethyl)quinazolin-4-amine (5c)

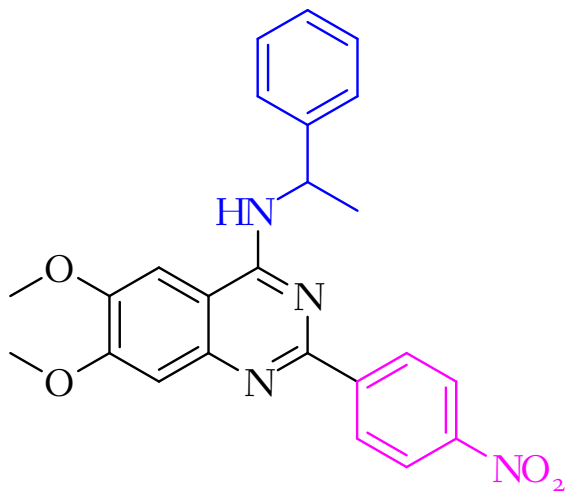

5c

Compound **5c** was prepared according to general procedure B using compound **3b** (0.2 g, 1.0 equiv.) and 4-nitro-phenylboronic acid as an yellow solid (0.188 g, 75%); Rf =0.39 (5% methanol in dichloromethane) ^{1}H NMR (400 MHz, $CDCl_3$): δ 8.50 (d, *J* = 8.0 Hz, 2H), 8.21 (d, *J* = 12.0 Hz, 2H), 7.44 (d, *J* = 4.0 Hz, 2H), 7.33 (t, *J* = 6.0 Hz, 2H), 7.24 - 7.19 (m, 2H), 5.67 - 5.61 (m, 1H), 5.54 (d, *J* = 8.0 Hz, 1H), 3.98 (s, 3H), 3.97 (s, 3H), 1.70 (d, *J* = 4.0 Hz, 3H). ^{13}C NMR (100 MHz, $CDCl_3$): δ 156.6, 156.1, 153.6, 148.5, 147.6, 146.4, 144.2, 142.9 127.8, 127.7, 126.4, 125.3, 122.4, 107.3, 106.5, 98.3, 55.4, 55.3, 49.7, 21.2. (ESI-MS) m/z: calcd. for $C_{24}H_{22}N_4O_4$: 430.46; found: 429.63 [M^{-1}]

2-(4-chlorophenyl)-6,7-dimethoxy-*N*-(4-(4-methylthiazol-5-yl)benzyl)quinazolin-4-amine(5d)

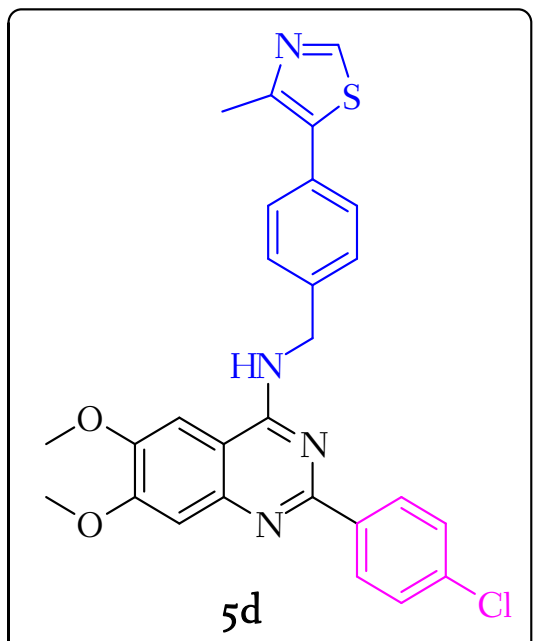

5d

Compound **5d** was prepared according to general procedure B using compound **3c** (0.2 g, 1.0 equiv.) and 4-chloro-phenylboronic acid as a light brown solid (0.191 g, 81%); Rf =0.42 (5% methanol in dichloromethane); ^{1}H NMR (400 MHz, DMSO-d_6): δ 8.97 (s, 1H), 8.70 (t, *J* = 6.0 Hz, 1H), 8.37 - 8.35 (m, 2H), 7.73 (s, 1H), 7.57 (d, *J* = 8.0 Hz, 2H), 7.47 (d, *J* = 8.0 Hz, 4H), 7.24 (s, 1H), 4.93 (d, *J* = 4.0 Hz, 2H), 3.94 (s, 3H), 3.93 (s, 3H), 2.43 (s, 3H). ^{13}C NMR (100 MHz, DMSO-d_6): δ 159.0, 156.8, 154.6, 151.93, 149.1, 148.3, 147.1, 140.5, 133.6, 131.6, 130.6, 130.4, 129.7, 129.4, 128.4, 127.6, 126.4, 108.0, 108.0, 102.5, 56.5, 56.2, 44.1, 21.5, 16.4. (ESI-MS) m/z: calcd. for $C_{27}H_{23}ClN_4O_2S$: 503.02; found: 504.23 [M^{+1}]

2-(3-chlorophenyl)-6,7-dimethoxy-*N*-(4-(4-methylthiazol-5-yl)benzyl)quinazolin-4-amine (5e)

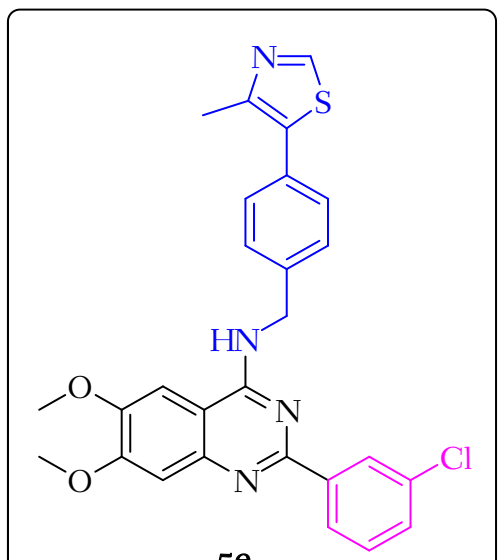

5e

Compound **5e** was prepared according to general procedure B using compound **3c** (0.2 g, 1.0 equiv.) and 3-chloro-phenylboronic acid as an off white solid (0.186 g, 79%); Rf = 0.41 (5% methanol in dichloromethane); ^{1}H NMR (400 MHz, DMSO-d_6): δ 9.10 (t, *J* = 6.0 Hz, 1H), 8.97 (s, 1H), 8.35 - 8.33 (m, 2H), 7.93 (s, 1H), 7.59 (d, *J* = 8.0 Hz, 2H), 7.49 - 7.45 (m, 4H), 7.21 (s, 1H), 4.89 (d, *J* = 4.0 Hz, 2H), 3.94 (s, 3H), 3.93 (s, 3H), 2.49 (s, 3H). ^{13}C NMR (100 MHz, DMSO-d_6): δ 159.0, 156.8, 154.6, 151.9, 149.1, 148.3, 147.1, 141.5, 140.5, 133.6, 130.6, 130.4, 129.8, 129.4, 128.4, 127.6, 127.4, 108.0, 108.0, 102.5, 56.5, 56.2, 16.4. (ESI-MS) m/z: calcd. for $C_{27}H_{23}ClN_4O_2S$: 503.02; found: 504.70 [M^{+1}]

6,7-dimethoxy-2-(4-methoxyphenyl)-*N*-(4-(4-methylthiazol-5-yl)benzyl)quinazolin-4-amine (5f)

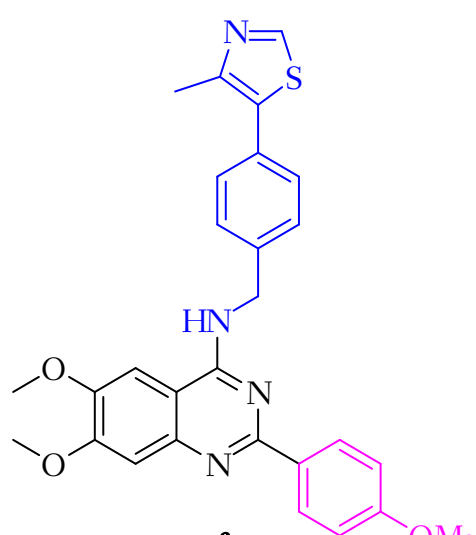

5f

Compound **5f** was prepared according to general procedure B using compound **3c** (0.2 g, 1.0 equiv.) and 4-methoxy-phenylboronic acid as an white solid (0.205 g, 88%); Rf =0.40 (5% methanol in dichloromethane); ^{1}H NMR (400 MHz, DMSO-d_6): δ 9.76 (brs, 1H), 9.04 (s, 1H), 8.36 (d, *J* = 8.0 Hz, 2H), 7.88 (s, 1H), 7.63 (d, *J* = 8.0 Hz, 2H), 7.55 (d, *J* = 8.0 Hz, 2H), 7.42 (s, 1H), 7.19 (d, *J* = 8.0 Hz, 2H), 5.08 (d, *J* = 4.0 Hz, 2H), 4.02 (s, 3H), 3.99 (s, 3H), 3.92 (s, 3H), 2.49 (s, 3H). ^{13}C NMR (100 MHz, DMSO-d_6): δ 163.0, 159.0, 155.7, 152.1, 149.5, 148.4, 139.0, 131.4, 130.9, 130.6, 129.6, 128.7, 114.7, 106.2, 103.3, 56.8, 56.7, 56.0, 44.6, 16.4. (ESI-MS) m/z: calcd. for $C_{28}H_{26}N_4O_3S$: 498.60; found: 499.25 $[M^{+1}]$

4-(6,7-dimethoxy-4-((4-(4-methylthiazol-5-yl)benzyl)amino)quinazolin-2-yl)benzonitrile (5g)

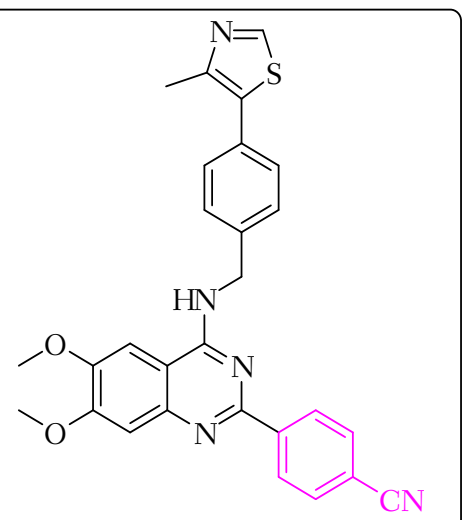

5g

Compound **5g** was prepared according to general procedure B using compound **3c** (0.2 g, 1.0 equiv.) and 4-cyano-phenylboronic acid as a off white solid (0.171 g, 74%); Rf = 0.44 (5% methanol in dichloromethane). ^{1}H NMR (400 MHz, DMSO-d_6): δ 8.98 (s, 1H), 8.74 (t, *J* = 4.0 Hz, 1H), 8.58 (d, *J* = 8.0 Hz, 2H), 7.93 (d, *J* = 8.0 Hz, 2H), 7.74 (s, 1H), 7.57 (d, *J* = 8.0 Hz, 2H), 7.48 (d, *J* = 8.0 Hz, 2H), 7.24 (s, 1H), 4.96 (d, *J* = 8.0 Hz, 2H), 3.95 (s, 3H), 3.93 (s, 3H), 2.43 (s, 3H). ^{13}C NMR (100 MHz, DMSO-d_6): δ 159.0, 156.5, 154.6, 152.0, 149.0, 148.3, 147.0, 143.6, 140.4, 132.8, 131.6, 130.5, 129.4, 128.6, 128.5, 119.4, 112.2, 108.1, 108.0, 102.5, 56.5, 56.3, 44.0, 16.4. (ESI-MS) m/z: calcd. for $C_{28}H_{23}N_5O_2S$: 493.59; found: 492.50 $[M^{-1}]$

6,7-dimethoxy-*N*-(4-(4-methylthiazol-5-yl)benzyl)-2-(4-nitrophenyl)quinazolin-4-amine (5h)

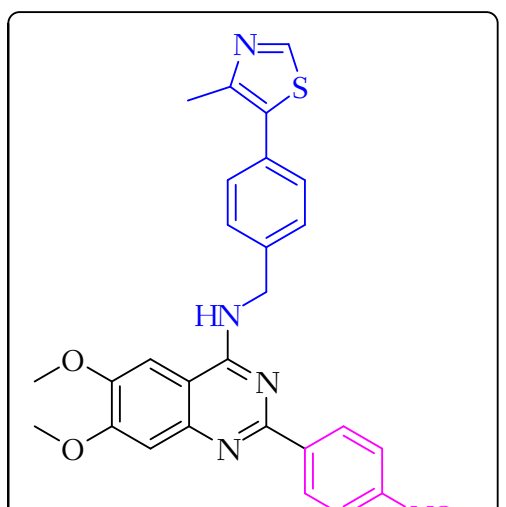

5h

Compound **5h** was prepared according to general procedure B using compound **3c** (0.2 g, 1.0 equiv.) and 4-nitro- phenylboronic acid as a yellow solid (0.183 g, 76%); Rf =0.42 (5% methanol in dichloromethane). ^{1}H NMR (400 MHz, DMSO-d_6): δ 9.02 (s, 1H), 8.80 (s, 1H), 8.69 (d, *J* = 8.0 Hz, 2H), 8.36 (d, *J* = 8.0 Hz, 2H), 7.79 (s, 1H), 7.63 (d, *J* = 4.0 Hz, 2H), 7.53 (d, *J* = 8.0 Hz, 2H), 7.31 (s, 1H), 5.01 (d, *J* = 4.0 Hz, 2H), 4.00 (s, 3H), 3.98 (s, 3H), 2.48 (s, 3H). ^{13}C NMR (100 MHz, DMSO-d_6): δ 172.5, 159.0, 156.2, 154.6, 152.0, 149.5, 148.5, 148.3, 147.0, 145.5, 131.6, 130.5, 129.9, 128.6, 124.0, 108.1, 108.1, 102.5, 56.5, 56.3, 44.1, 16.4. (ESI-MS) m/z: calcd. for $C_{27}H_{23}N_5O_4S$: 513.57; found: 512.90 $[M^{-1}]$

6,7-dimethoxy-*N*-(4-(4-methylthiazol-5-yl)benzyl)-2-(3-nitrophenyl)quinazolin-4-amine (5i)

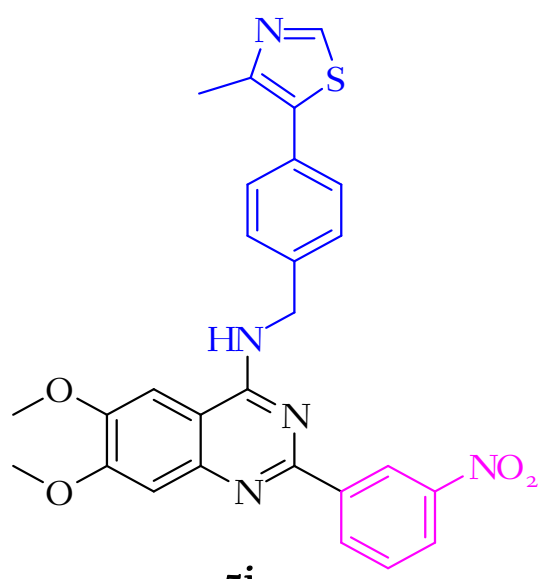

5i

Compound **5i** was prepared according to general procedure B using compound **3c** (0.2 g, 1.0 equiv.) and 3-nitro-phenylboronic acid as a yellow solid (0.176 g, 73%); Rf =0.38 (5% methanol in dichloromethane). ^{1}H NMR (400 MHz, DMSO-d$_6$): δ 9.16 (s, 1H), 8.96 (s, 1H), 8.80 - 8.75 (m, 2H), 8.27 (d, *J* = 8.0 Hz, 1H), 7.76-7.73 (m, 2H), 7.60 (d, *J* = 8.0 Hz, 2H), 7.48 (d, *J* = 8.0 Hz, 2H), 7.26 (s, 1H), 4.92 (d, *J* = 8.0 Hz, 2H), 3.95 (s, 3H), 3.93 (s, 3H), 2.42 (s, 3H). ^{13}C NMR (100 MHz, DMSO-d$_6$): δ 159.0, 156.0, 154.6, 151.9, 149.3, 148.5, 148.3, 147.0, 141.1, 140.4, 134.0, 131.6, 130.5, 130.3, 129.4, 128.6, 124.5, 122.3, 108.1, 108.0, 102.5, 56.5, 56.3, 44.2, 16.4. (ESI-MS) m/z: calcd. for $C_{27}H_{23}N_5O_4S$: 513.57; found: 512.90[M^{-1}]

2-(3,5-bis(trifluoromethyl)phenyl)-6,7-dimethoxy-*N*-(4-(4-methylthiazol-5-yl)benzyl)quinazolin-4-amine (5j)

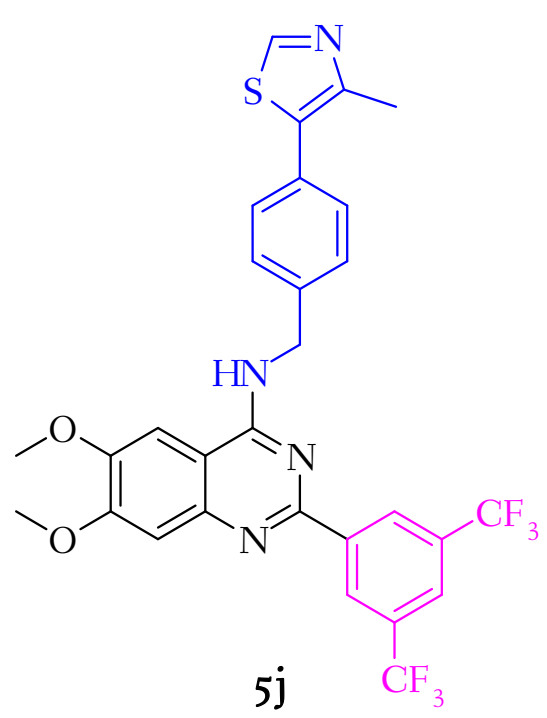

5j

Compound **5j** was prepared according to general procedure B using compound **3c** (0.2 g, 1.0 equiv.) and 3,5-di(trifluoromethyl-phenylboronic acid as a white solid (0.204 g, 72%); Rf = 0.40 (5% methanol in dichloromethane). ^{1}H NMR (400 MHz, DMSO-d$_6$): δ 8.96 (s, 1H), 8.80 - 8.75 (m, 2H), 8.91 - 8.88 (m, 3H), 8.16 (s, 1H), 7.57 (d, *J* = 8.0 Hz, 2H), 7.46 (d, *J* = 8.0 Hz, 2H), 7.30 (s, 1H), 4.88 (d, *J* = 4.0 Hz, 2H), 3.95 (s, 3H), 3.95 (s, 3H), 2.41 (s, 3H). ^{13}C NMR (100 MHz, DMSO-d$_6$): δ 159.0, 155.0, 154.7, 152.0, 149.5, 148.2, 146.8, 141.8, 140.4, 131.5, 131.0, 130.7, 130.4, 129.4, 128.2, 127.8, 125.5, 123.3, 108.3, 108.2, 102.5, 56.5, 56.3, 44.6, 16.3. (ESI-MS) m/z: calcd. for $C_{29}H_{22}F_6N_4O_2S$: 604.57; found: 605.42 [M^{+1}]

2-(3,5-di-*tert*-butylphenyl)-6,7-dimethoxy-*N*-(4-(4-methylthiazol-5-yl)benzyl)quinazolin-4-amine (5k)

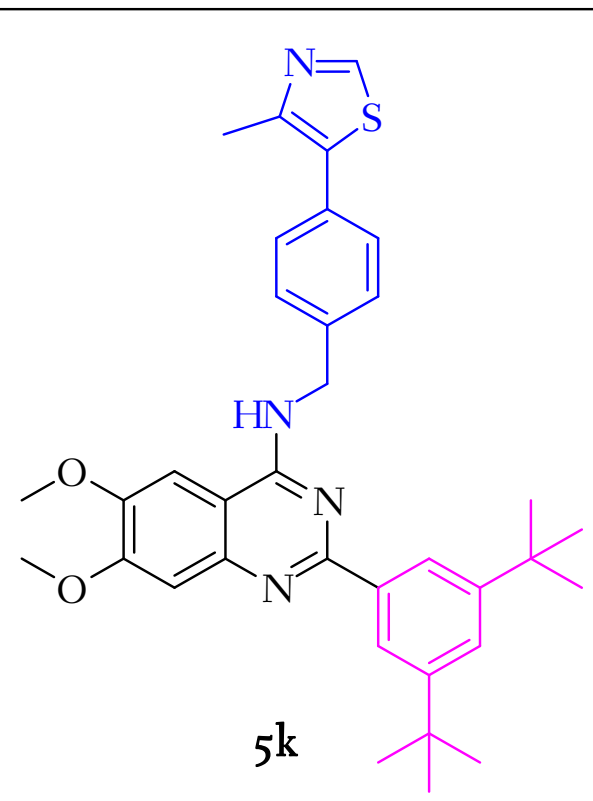

5k

Compound **5k** was prepared according to general procedure B using compound **3c** (0.2 g, 1.0 equiv.) and 3,5-di(*tert*-butyl)-phenylboronic acid as a white solid (0.199 g, 73%); Rf = 0.32 (5% methanol in dichloromethane). ^{1}H NMR (400 MHz, DMSO-d$_6$): δ 8.97 (s, 1H), 8.67 (t, *J* = 4.0 Hz, 2H), 8.26 (d, *J* = 4.0 Hz, 2H), 7.72 (s, 1H), 7.58 (d, *J* = 8.0 Hz, 2H), 7.46 - 7.44 (m, 3H), 7.22 (s, 1H), 4.89 (d, *J* = 4.0 Hz, 2H), 3.96 (s, 3H), 3.94 (s, 3H), 2.43 (s, 3H), 1.33 (s,18H). ^{13}C NMR (100 MHz, DMSO-d$_6$): δ 158.8, 158.8, 154.4, 151.9, 150.3, 149.3,

148.7, 148.2, 147.3, 140.8, 138.6, 131.6, 130.3, 129.4, 128.6, 128.2, 123.7, 122.2, 108.0, 107.7, 102.5, 56.4, 56.2, 44.2, 35.0, 31.8, 16.4. (ESI-MS) m/z: calcd. for $C_{35}H_{40}N_4O_2S$: 580.79; found: 582.10 $[M^{+1}]$

6,7-dimethoxy-*N*-(4-(4-methylthiazol-5-yl)benzyl)-2-(1*H*-pyrrol-2-yl)quinazolin-4-amine (5l)

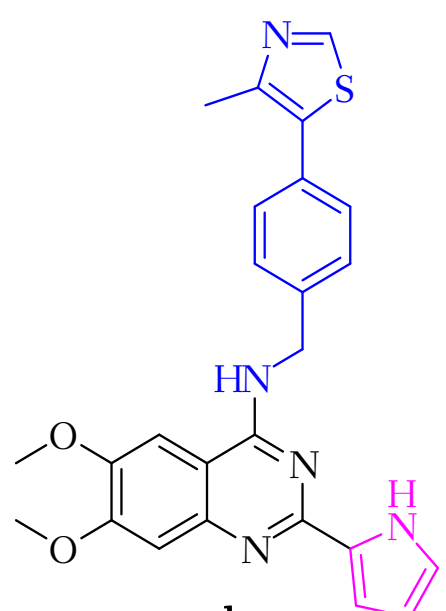

5l

Compound **5l** was prepared according to general procedure B using compound **3c** (0.2 g, 1.0 equiv.) and pyrrole-2-boronic acid as an off white solid (0.176 g, 82%); Rf = 0.23 (5% methanol in dichloromethane). ^{1}H NMR (400 MHz, DMSO-d_6): δ 11.27 (brs, 1H), 8.97 (s, 1H), 8.44 (t, *J* = 6.0 Hz, 1H), 7.64 (s, 1H), 7.58 (d, *J* = 8.0 Hz, 2H), 7.47 (d, *J* = 8.0 Hz, 2H), 7.07 (s, 1H), 6.85 (dd, *J* = 12.0, 4.0 Hz, 2H), 6.14 (d, *J* = 4.0 Hz, 1H), 7.24 (s, 1H), 4.98 (d, *J* = 4.0 Hz, 2H), 3.91 (s, 3H), 3.89 (s, 3H), 2.44 (s, 3H). ^{13}C NMR (100 MHz, DMSO-d_6): δ 158.6, 154.3, 151.9, 148.2, 147.9, 132.3, 131.6, 130.4, 129.3, 128.8, 120.9, 110.2, 109.5, 107.1, 102.8, 56.4, 56.0, 44.0, 16.4. (ESI-MS) m/z: calcd. for $C_{25}H_{23}N_5O_2S$: 457.55; found: 458.1$[M^{+1}]$

2-(5-((6,7-dimethoxy-2-(4-methoxyphenyl)quinazolin-4-yl)amino)pentan-2-yl)isoindoline-1,3-dione (5m)

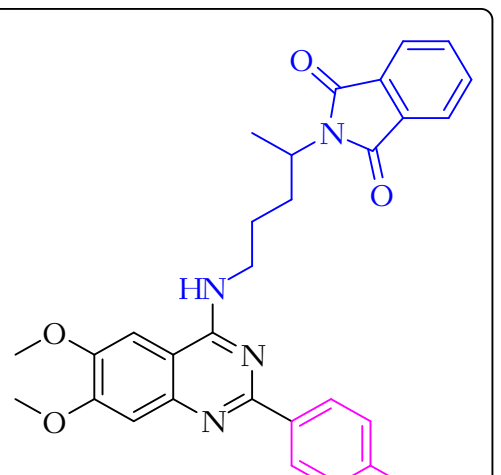

5m

Compound **5m** was prepared according to general procedure B using compound **3d** and 4-methoxy-phenylboronic acid as a white solid (0.194 g, 84%); Rf =0.41 (5% methanol in dichloromethane). ^{1}H NMR (400 MHz, DMSO-d_6): δ 8.35 (d, *J*= 12.0 Hz, 2H) 7.86 (s, 1H), 7.80 (s, 4H), 7.75 (s, 1H), 7.11 (s, 1H), 6.98 (d, *J*= 8.0 Hz, 2H), 4.40 - 4.34 (m, 1H), 3.91 (s, 3H), 3.87 (s, 3H), 3.83 (s, 3H), 3.65 - 3.60 (m, 2H), 2.51 (s, 2H), 2.18 - 2.12 (m, 1H), 1.87 - 1.79 (m, 1H), 1.69-1.66 (m, 2H), 1.41 (d, *J* = 4.0 Hz, 3H). ^{13}C NMR (100 MHz, DMSO-d_6): δ 168.51, 161.0, 158.9, 158.1, 154.2, 148.3, 147.2, 136.3, 134.8, 132.0, 131.8, 129.4, 123.4, 113.9, 113.4, 107.7, 107.5, 102.6, 56.4, 56.1, 55.6, 47.0, 31.2, 26.55, 19.1. (ESI-MS) m/z: calcd. for $C_{30}H_{30}N_4O_5$: 426.59; found: 426.90$[M^{+1}]$

2-(5-((6,7-dimethoxy-2-(4-nitrophenyl)quinazolin-4-yl)amino)pentan-2-yl)isoindoline-1,3-dione (5n)

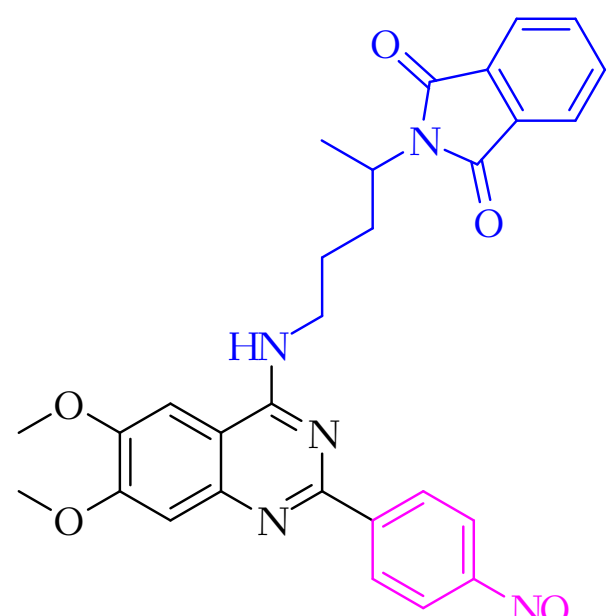

5n

Compound **5n** was prepared according to general procedure B using compound **3d** and 4-nitro-phenylboronic acid as a yellow solid (0.171 g, 72%); Rf = 0.40 (5% methanol in dichloromethane). ^{1}H NMR (400 MHz, DMSO-d$_6$): δ 8.66 (d, *J* = 8.0 Hz, 2H), 8.34 (d, *J* = 8.0 Hz, 2H), 8.13 (t, *J* = 4.0 Hz, 1H), 7.82 (s, 4H), 7.65 (s, 1H), 7.26 (s, 1H), 4.45 - 4.39 (m, 1H), 3.99 (s, 3H), 3.95 (s, 3H), 2.28 - 2.18 (m, 1H), 1.92 - 1.76 (m, 3H), 1.46 (d, *J* = 8.0 Hz, 3H). ^{13}C NMR (100 MHz, DMSO-d$_6$): δ 168.5, 159.0, 156.2, 154.4, 149.3, 148.4, 146.9, 145.6, 134.8, 131.7, 128.8, 123.9, 123.3, 108.1, 108.0, 102.5, 56.5, 56.2, 47.0, 31.1, 26.3, 19.1. (ESI-MS) m/z: calcd. for $C_{29}H_{27}N_5O_6$: 441.56; found: 442.0 [M^{+1}]

2-(5-((2-(3,5-bis(trifluoromethyl)phenyl)-6,7-dimethoxyquinazolin-4-yl)amino)pentan-2-yl)isoindoline-1,3-dione (5o)

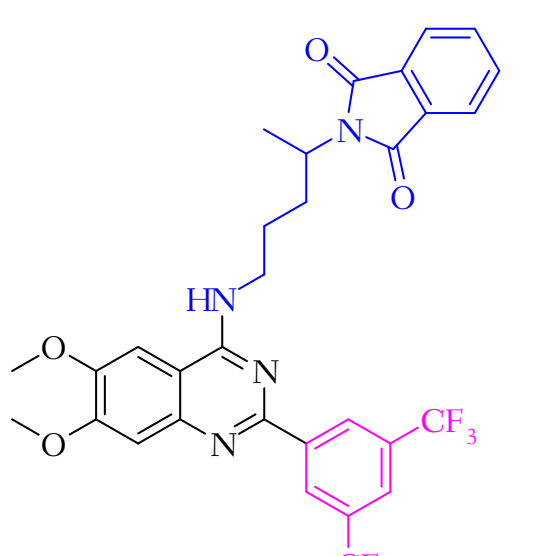

5o

Compound **5o** was prepared according to general procedure B using compound **3d** and 3,5-di(trifluoromethyl-phenylboronic acid as an off white solid (0.206 g, 74%); Rf = 0.43 (5% methanol in dichloromethane). ^{1}H NMR (400 MHz, DMSO-d$_6$): δ 8.90 (s, 2H), 8.14 (d, *J* = 8.0 Hz, 2H), 7.74-7.68 (m, 4H), 7.59 (s, 1H), 7.24 (s, 1H), 4.36 - 4.33 (m, 1H), 3.94 (s, 3H), 3.90 (s, 3H), 3.63 (d, *J* = 4.0 Hz, 2H), 2.22 - 2.14 (m, 1H), 1.81 - 1.67 (m, 3H), 1.38 (d, *J* = 4.0 Hz, 3H). ^{13}C NMR: (100 MHz, DMSO-d$_6$) δ 168.3, 159.1, 155.0, 154.5, 149.3, 146.6, 141.9, 134.7, 131.6, 131.0, 130.7, 127.7, 125.3, 123.2, 122.5, 108.3, 108.1, 102.6, 56.5, 56.3, 46.9, 31.0, 26.0, 19.0. (ESI-MS) m/z: calcd. for $C_{31}H_{26}F_6N_4O_4$: 632.56; found: 633.45[M^{+1}]

2-(5-((6,7-dimethoxy-2-(3-nitrophenyl)quinazolin-4-yl)amino)pentan-2-yl)isoindoline-1,3-dione (5p)

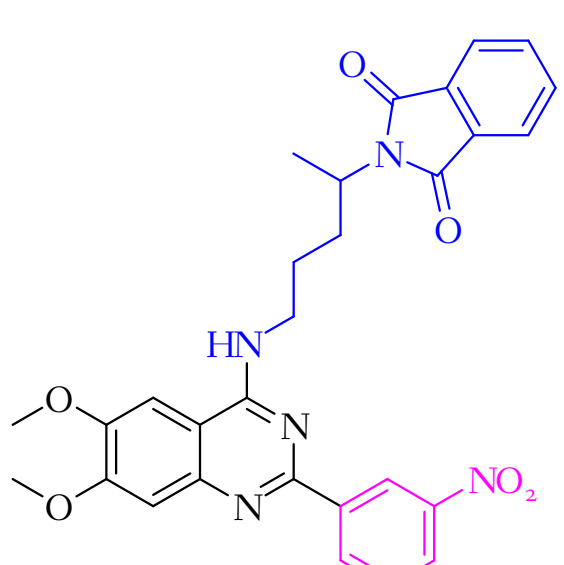

5p

Compound **5p** was prepared according to general procedure B using compound **3d** and 3-nitro-phenylboronic acid as a yellow solid (0.157 g, 66%); Rf =0.38 (5% methanol in dichloromethane). ^{1}H NMR (400 MHz, DMSO-d$_6$): δ 9.21 (s, 1H), 8.84 (d, *J* = 4.0 Hz, 1H), 8.34 (d, *J* = 8.0 Hz, 1H), 8.15 - 8.12 (m, 1H), 7.83 - 7.77 (m, 5H), 7.65 (s, 1H), 7.29 (s, 1H), 4.44 - 4.38 (m, 1H), 3.99 (s, 3H), 3.95 (s, 3H), 3.70 (d, *J* = 4.0 Hz, 2H), 2.25 - 2.20 (m, 1H), 1.96 - 1.75 (m, 3H), 1.46 (d, *J* = 4.0 Hz, 3H). ^{13}C NMR (100 MHz, DMSO-d$_6$): δ 168.5, 159.1, 156.0, 154.5, 149.1, 148.5, 146.8, 141.2, 134.8, 133.9,

131.7, 130.3, 124.5, 123.3, 122.1, 108.1, 108.0, 102.6, 56.5, 56.3, 47.0, 31.1, 26.2, 19.1. (ESI-MS) m/z: calcd. for $C_{29}H_{27}N_5O_6$: 541.56; found: 542.2[M^{+1}]

4-(4-((4-(1,3-dioxoisoindolin-2-yl)pentyl)amino)-6,7-dimethoxyquinazolin-2-yl)benzonitrile (5q)

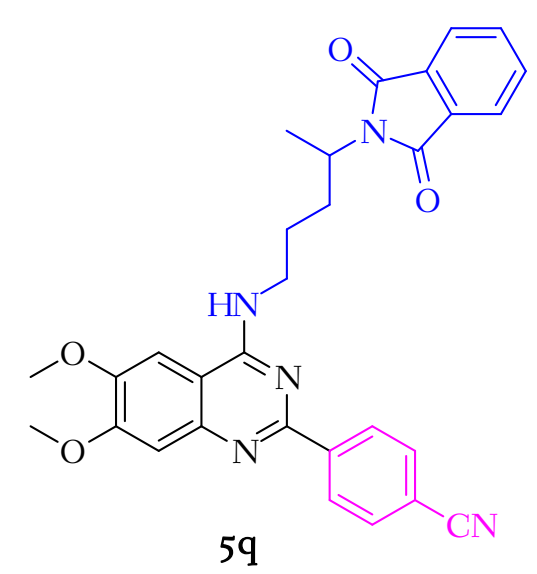

5q

Compound **5q** was prepared according to general procedure B using compound **3d** and 4-cyano-phenylboronic acid as a yellow solid (0.126 g, 64%); Rf =0.46 (5% dichloromethane to methanol). **5q** was isolated in impure form after multiple purifications. ^{1}H NMR (400 MHz, DMSO-d$_6$): δ 8.54 (d, *J* = 8.0 Hz, 1H), 8.44 (s, 1H), 8.05 (s, 1H), 7.89 (d, *J* = 8.0 Hz, 2H), 7.77 (s, 4H), 7.59 (s, 1H), 7.18 (s, 1H), 4.38 - 4.32 (m, 1H), 3.93 (s, 3H), 3.89 (s, 3H), 3.63 (d, *J* = 4.0 Hz, 2H), 2.17 - 2.13 (m, 1H), 1.82 - 1.78 (m, 1H), 1.69 - 1.65 (m, 2H), 1.40 (d, *J* = 8.0 Hz, 3H). ^{13}C NMR (100 MHz, DMSO-d$_6$) δ 168.5, 159.0, 156.5, 154.4, 149.2, 148.6, 146.9, 135.1, 134.8, 134.7, 132.7, 131.7, 131.5, 128.4, 123.3, 119.5, 116.9, 1222.1, 108.1, 108.0, 102.5, 56.5, 56.2, 47.0, 31.1, 26.3, 19.1. (ESI-MS) m/z: calcd. for $C_{30}H_{27}N_5O_4$: 521.58; found: 522.43 [$M^{+}1$]

5.5 Spectral data for the synthesized products 3a-3d and 5a-5q

Note# The ^{1}H and ^{13}C NMR spectra were recorded in DMSO-d$_6$ and $CDCl_3$. During the analysis of the ^{1}H NMR spectra of all synthesized derivatives, minor grease-related impurity signals were observed in the region of δ 0.86-1.35 ppm, appearing as multiplet resonances. In addition, signals observed at approximately δ 30 ppm in the ^{13}C NMR spectra were also attributed to trace grease contamination. These extraneous peaks likely originated from the NMR solvent and do not correspond to the synthesized compounds.

3a 1H NMR

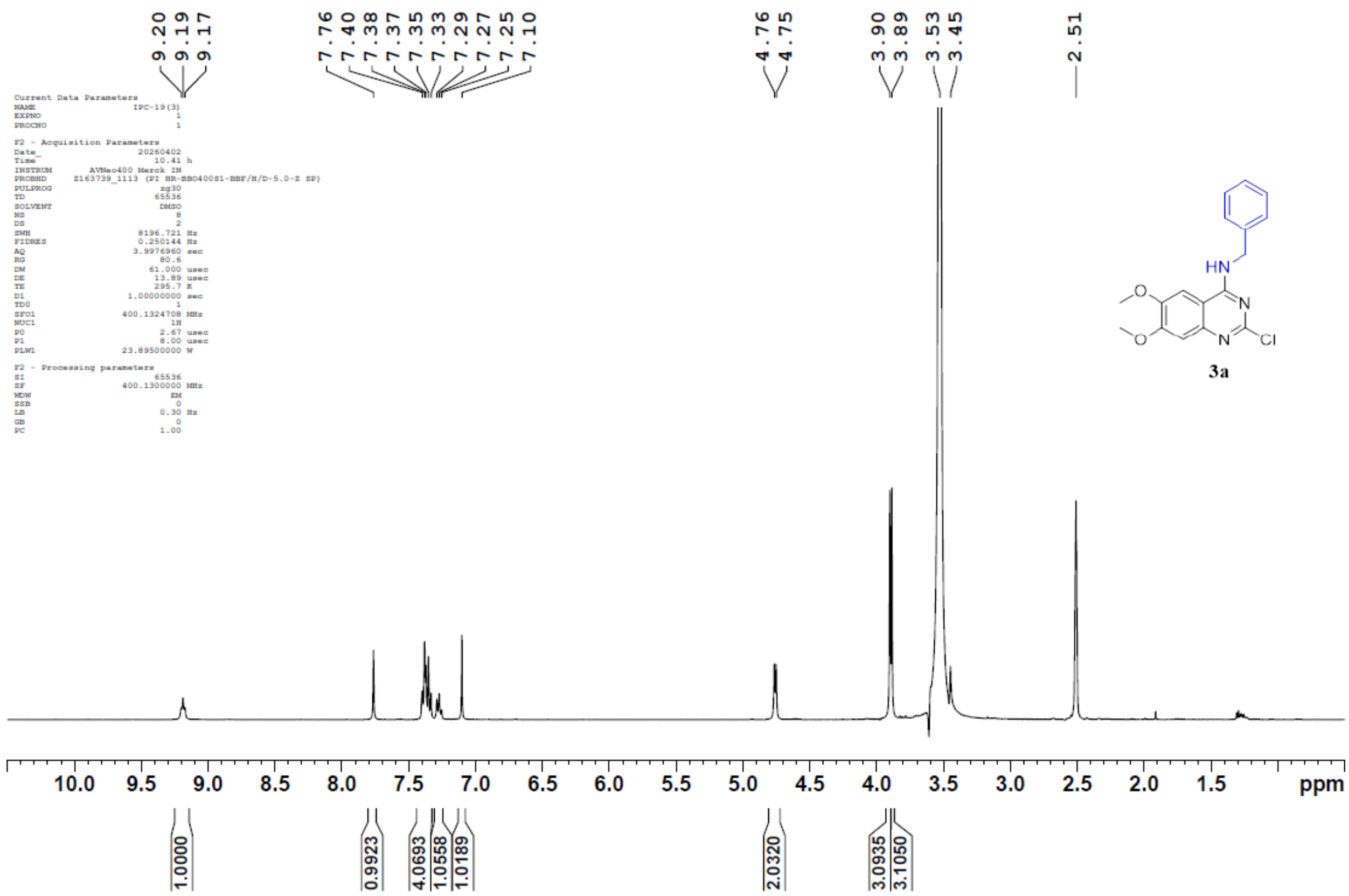

3b 1H NMR

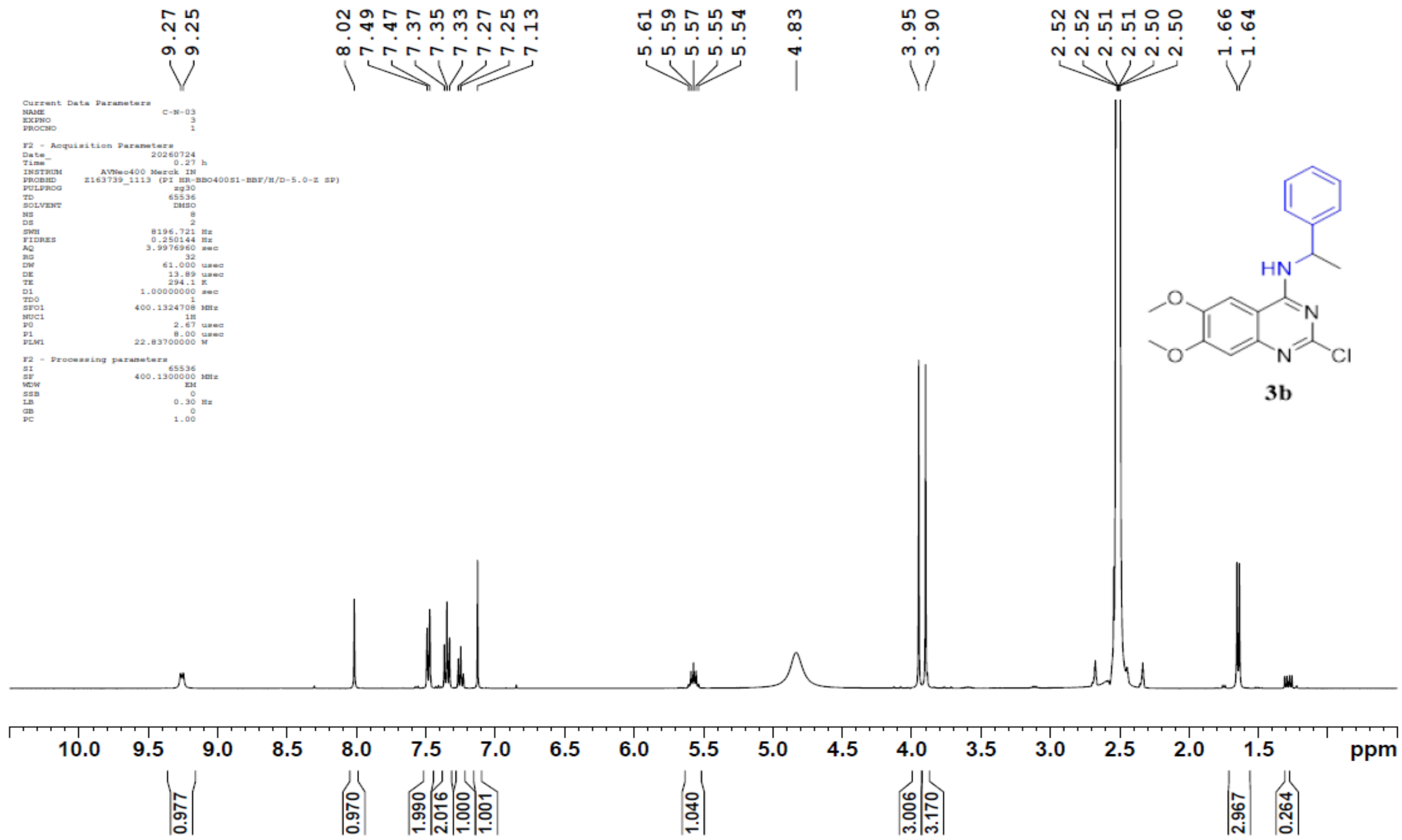

3b 13C NMR

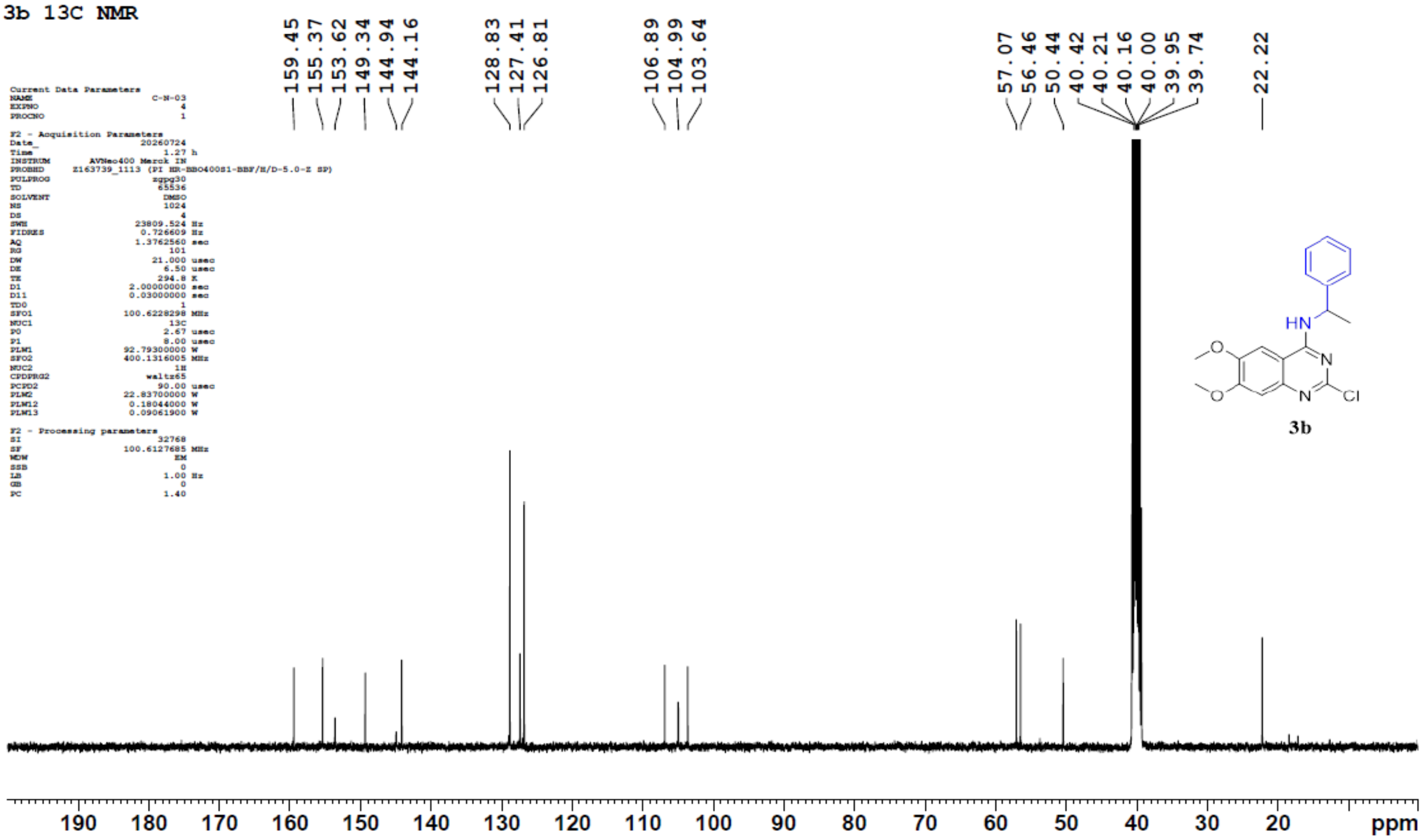

3c 1H NMR

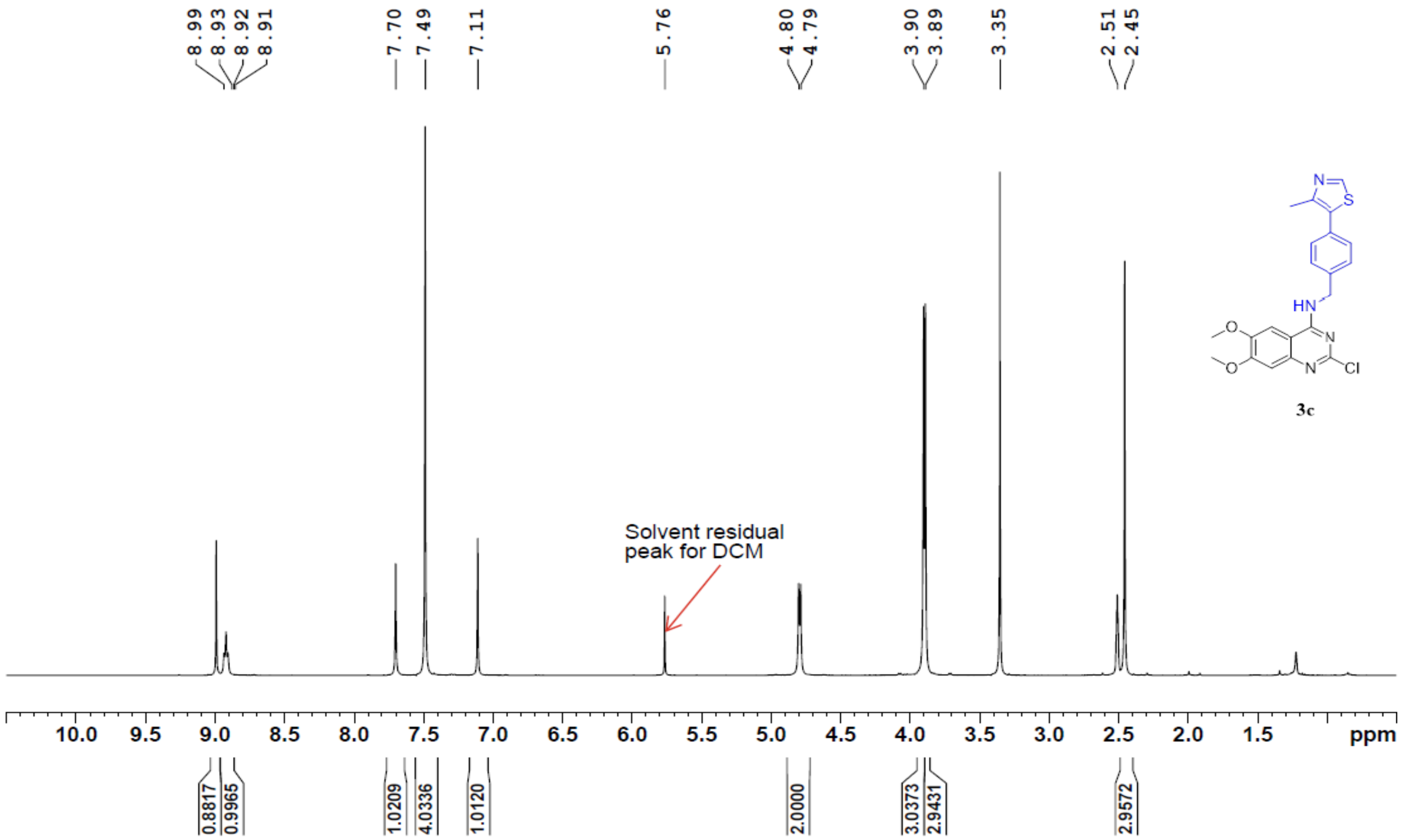

3c 13C NMR

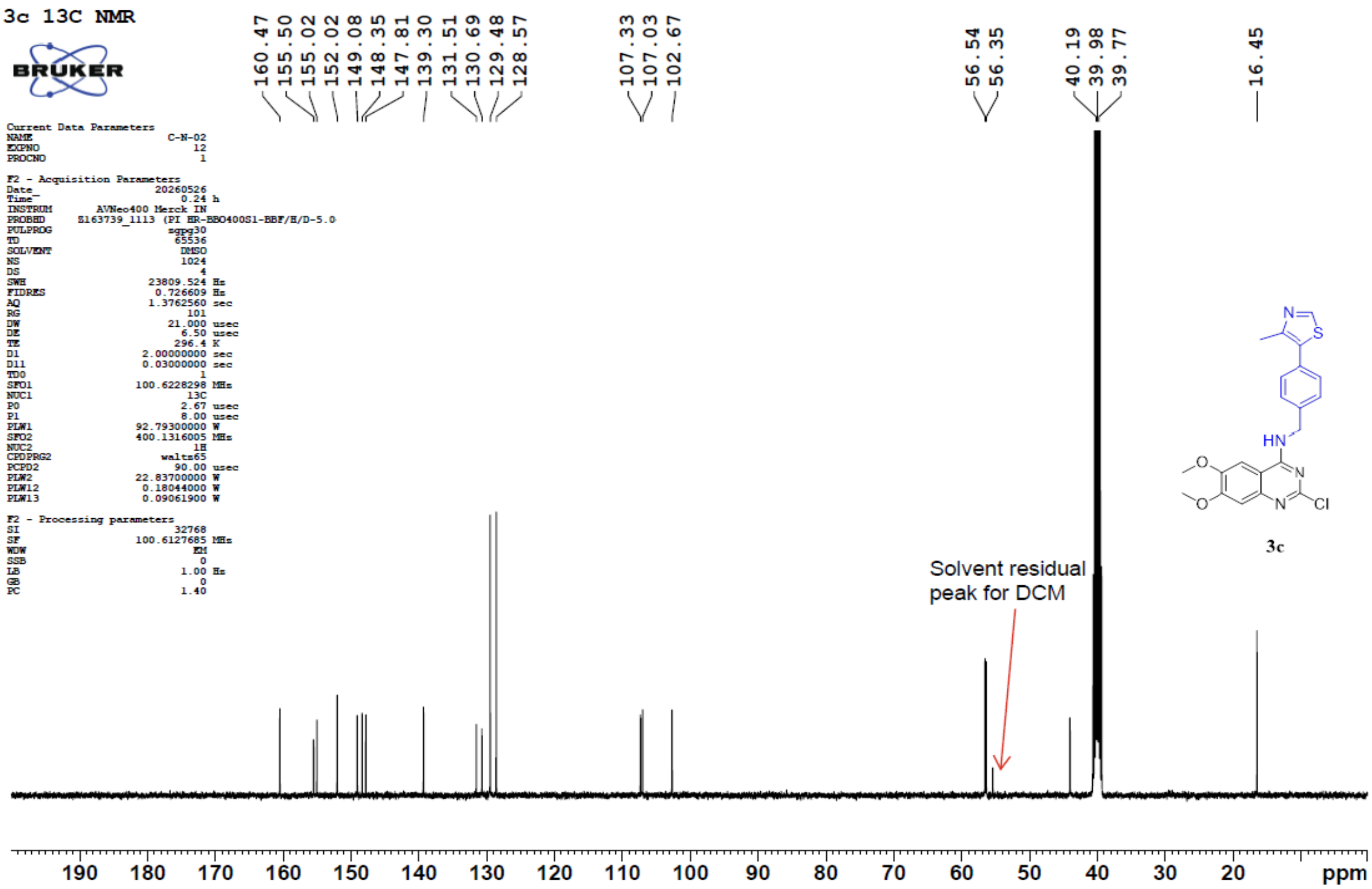

3d 1H NMR

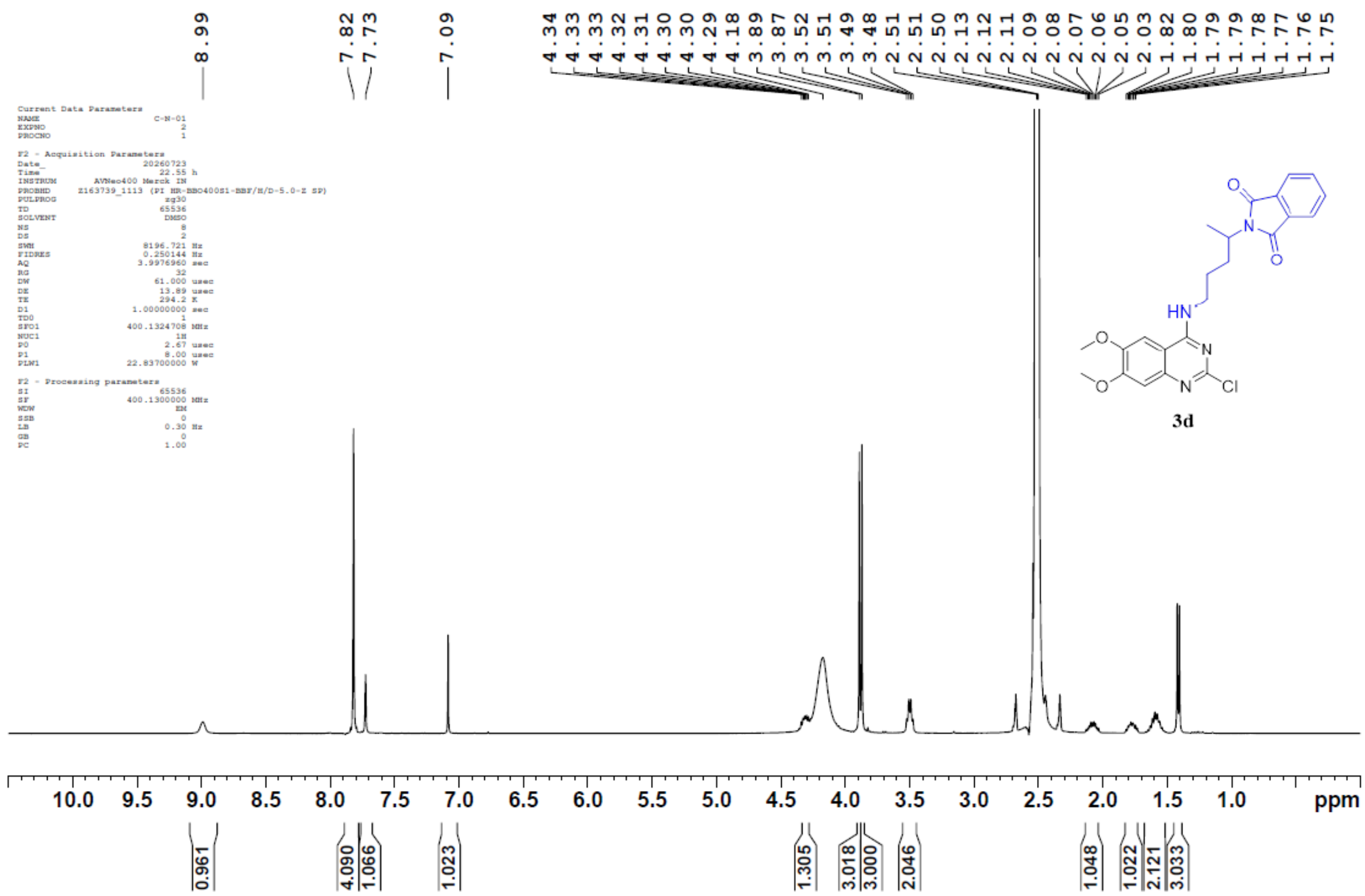

3d 13C NMR

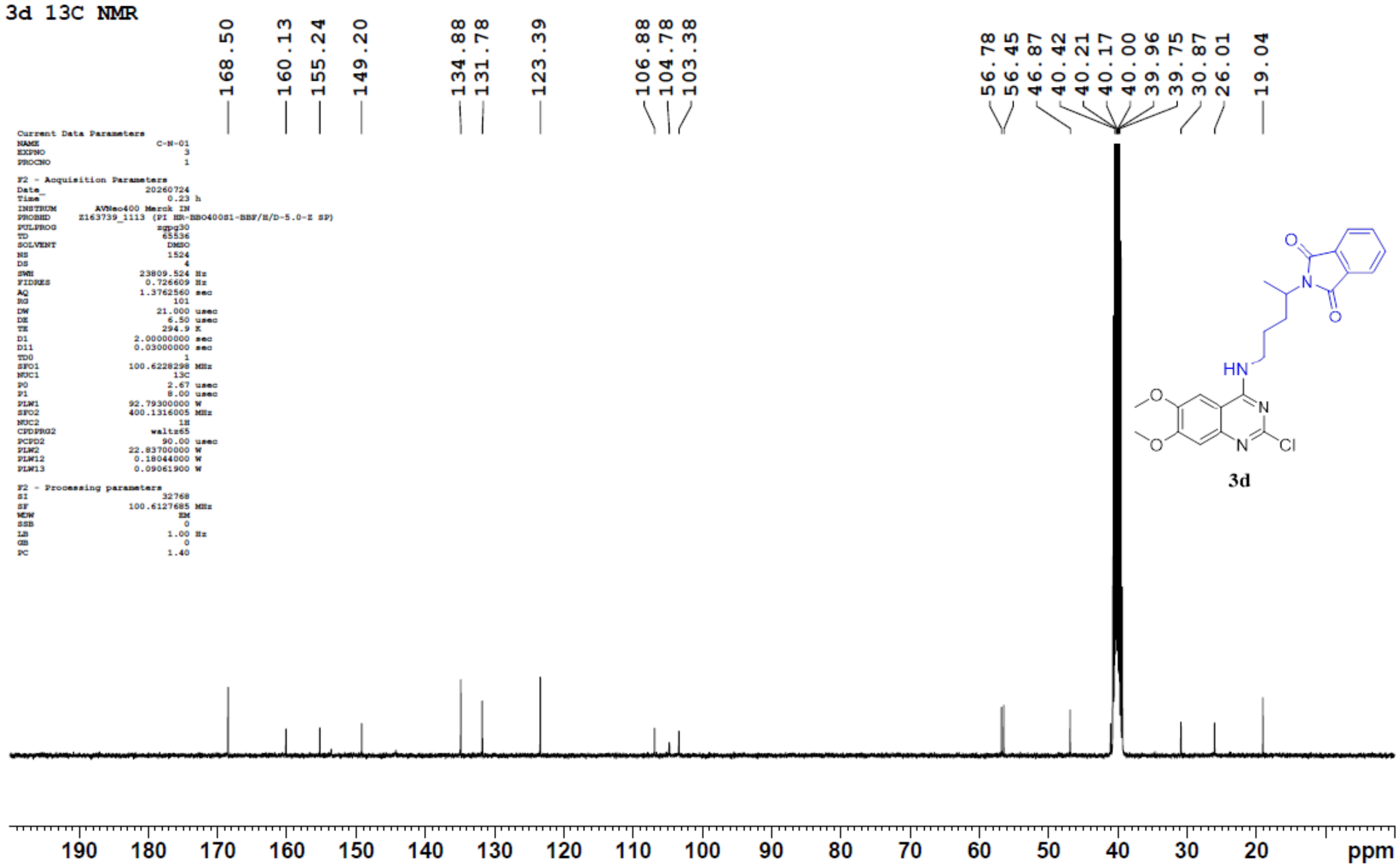

5a 1H NMR

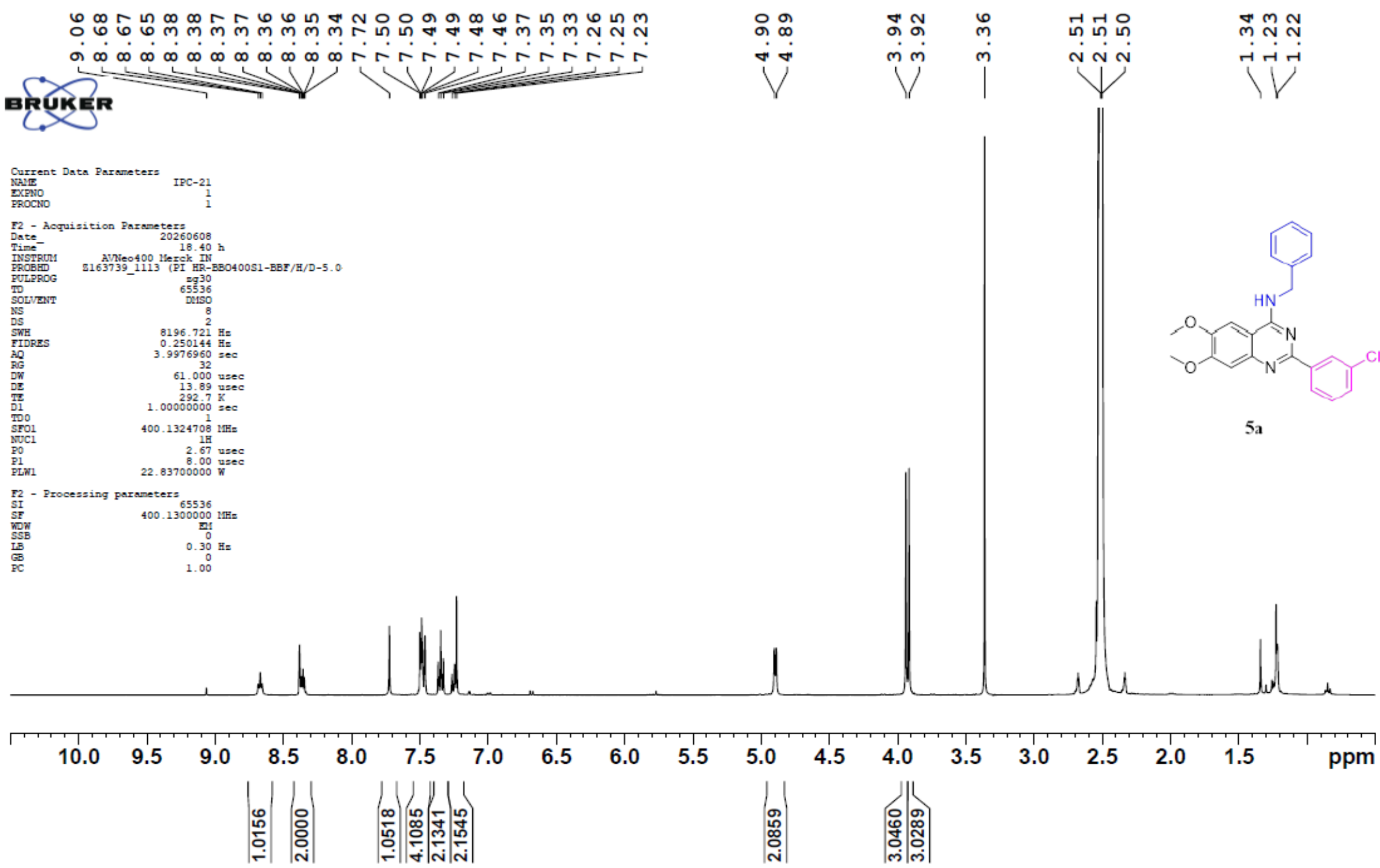

5a 13C NMR

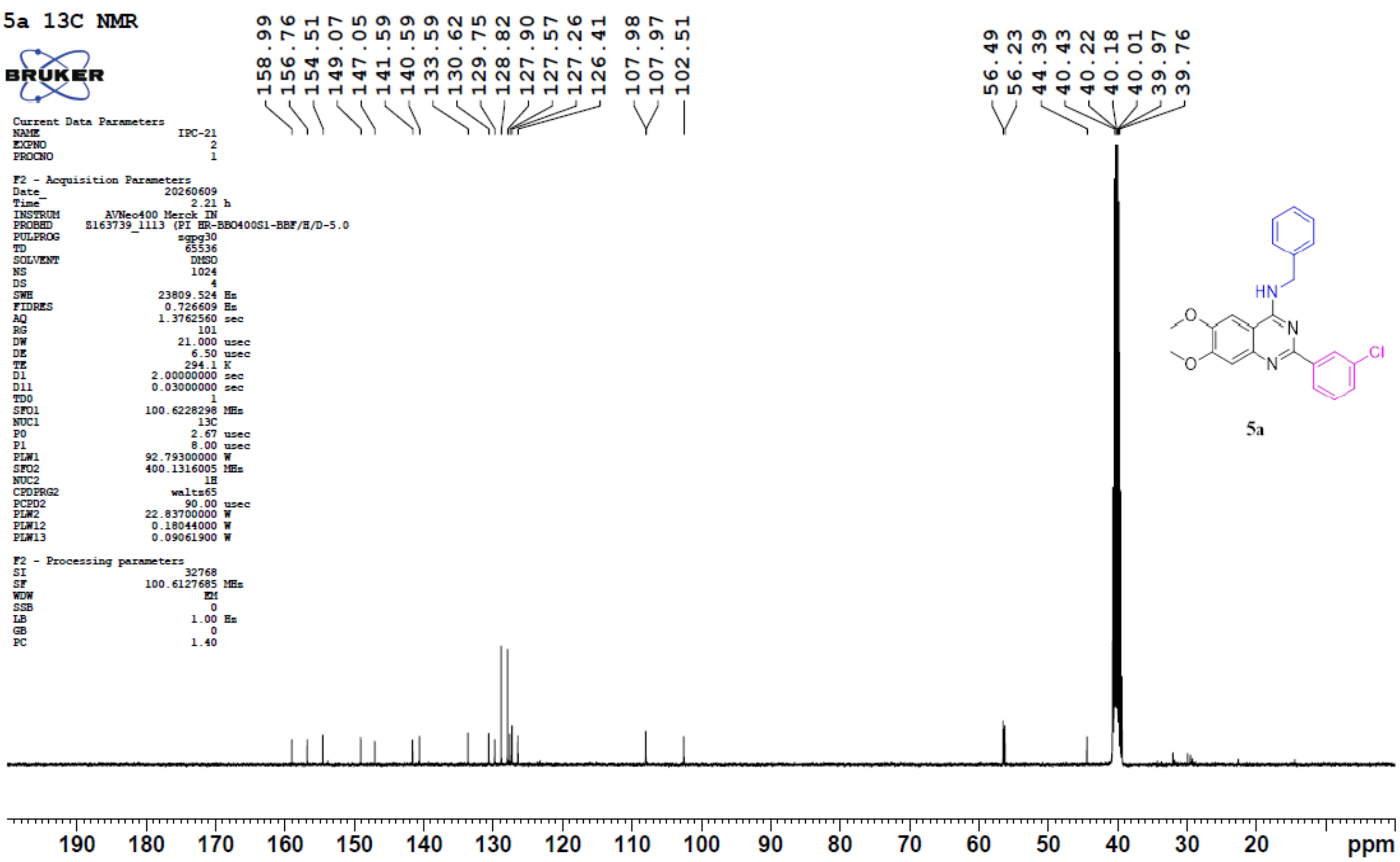

5b 1H NMR

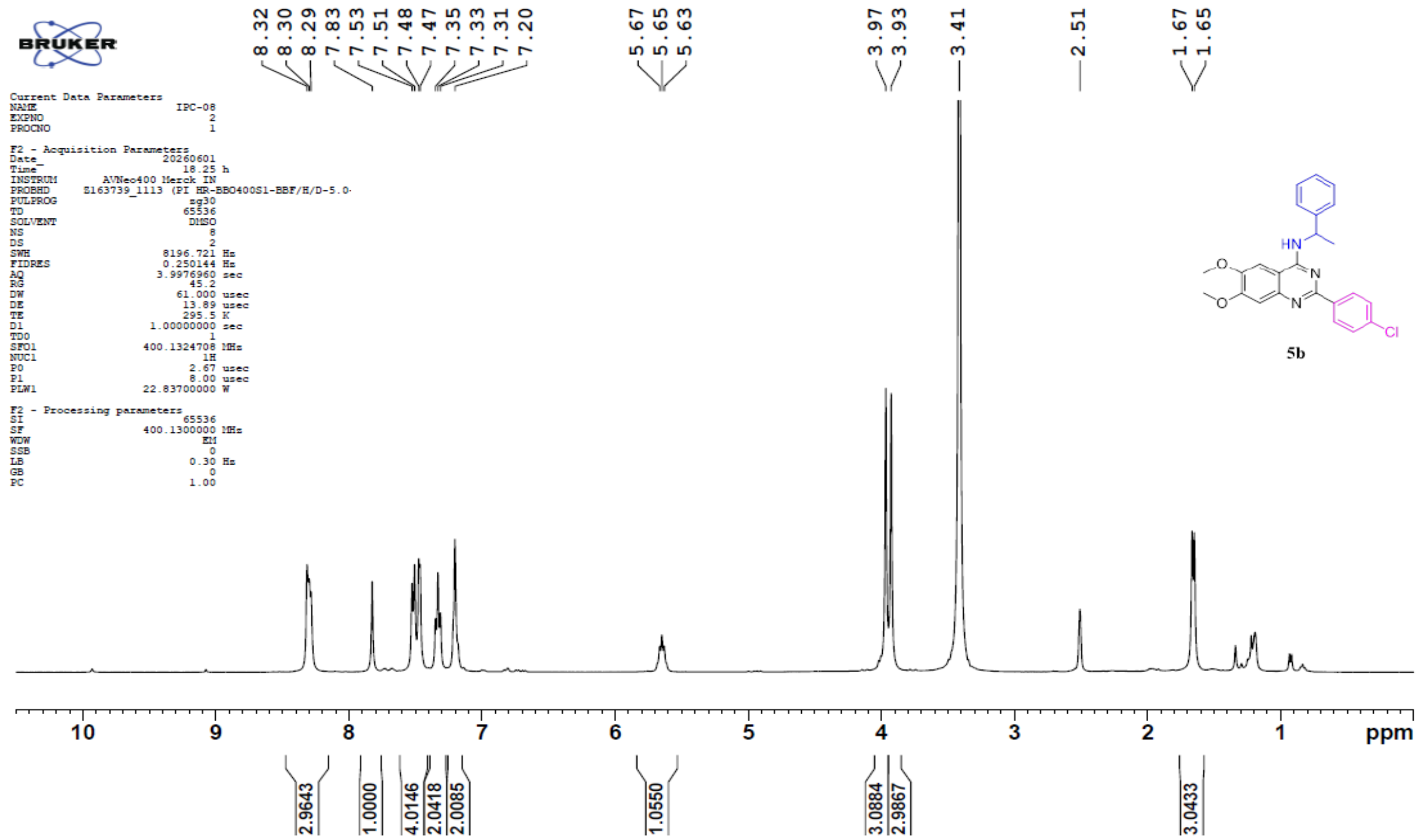

5b 13C NMR

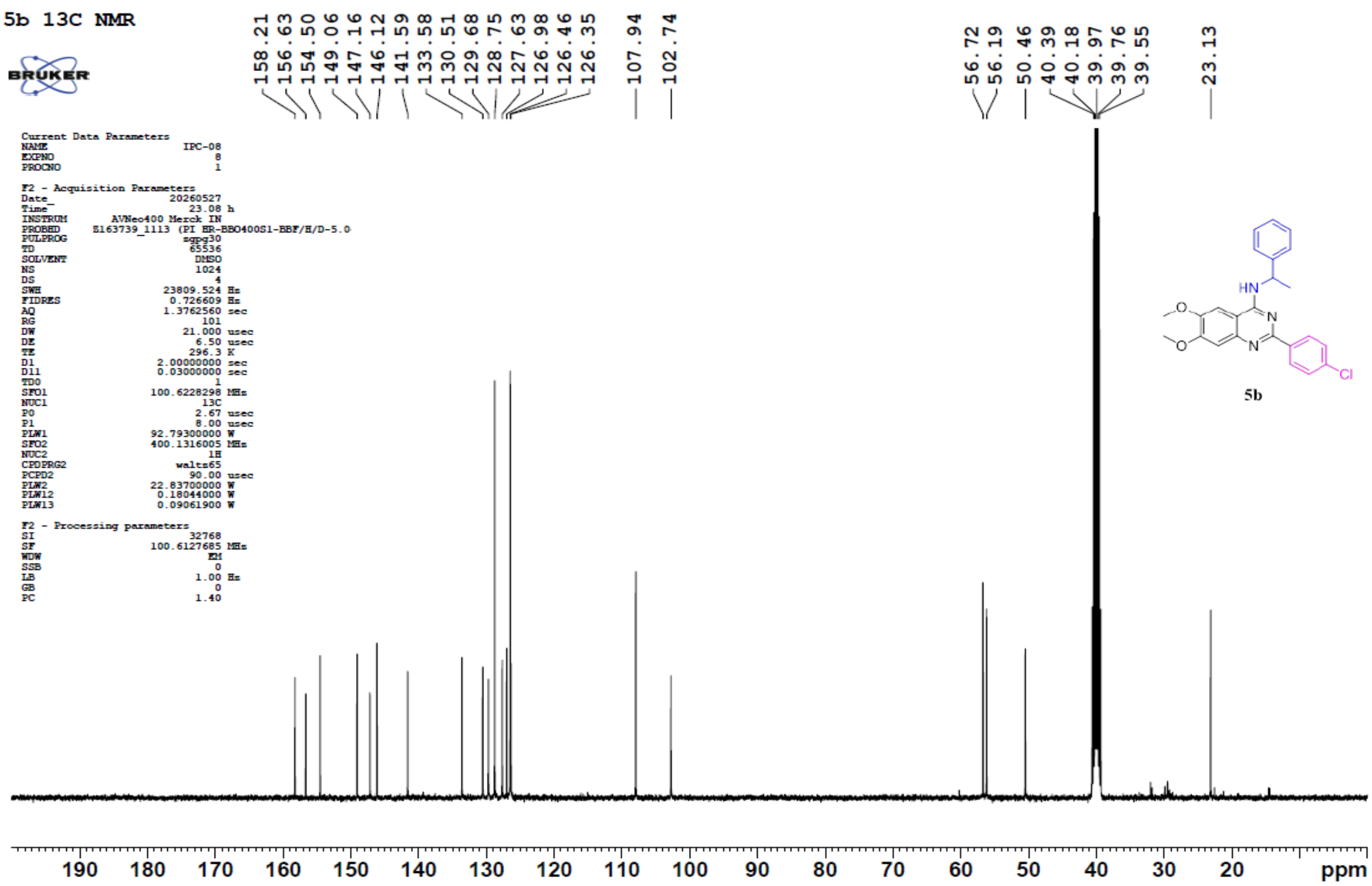

5c 1H NMR

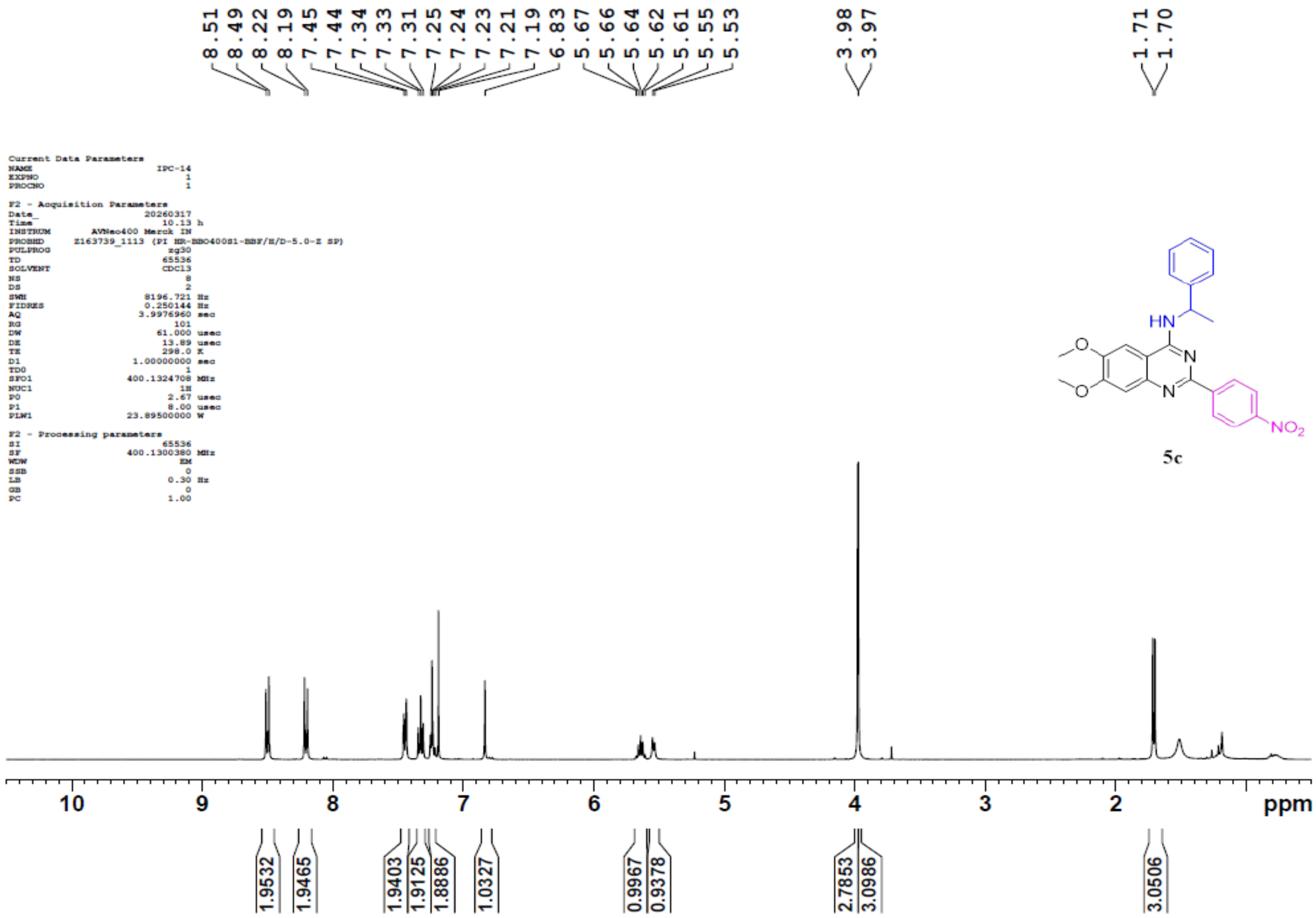

5c 13C NMR

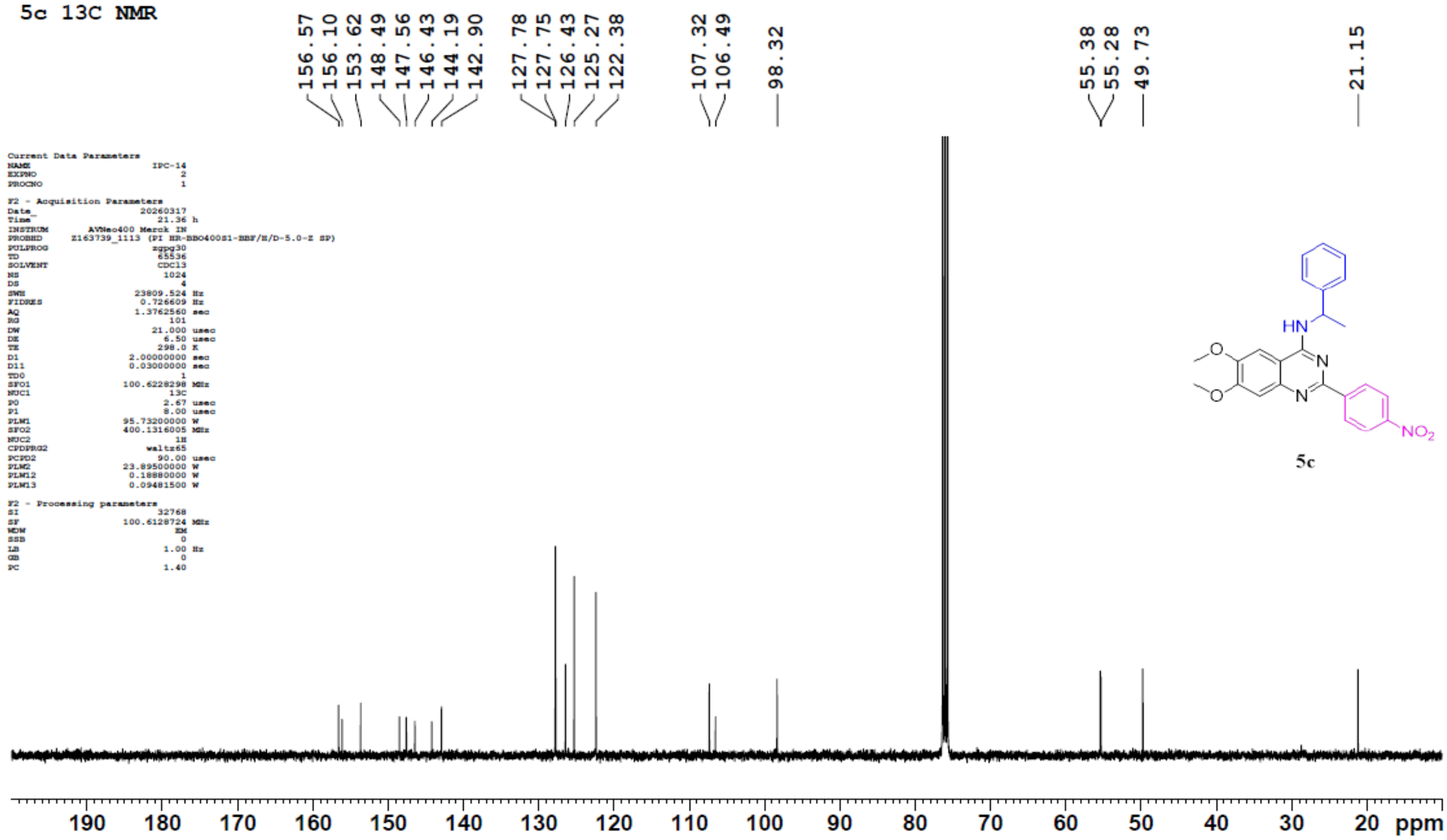

5d 1H NMR

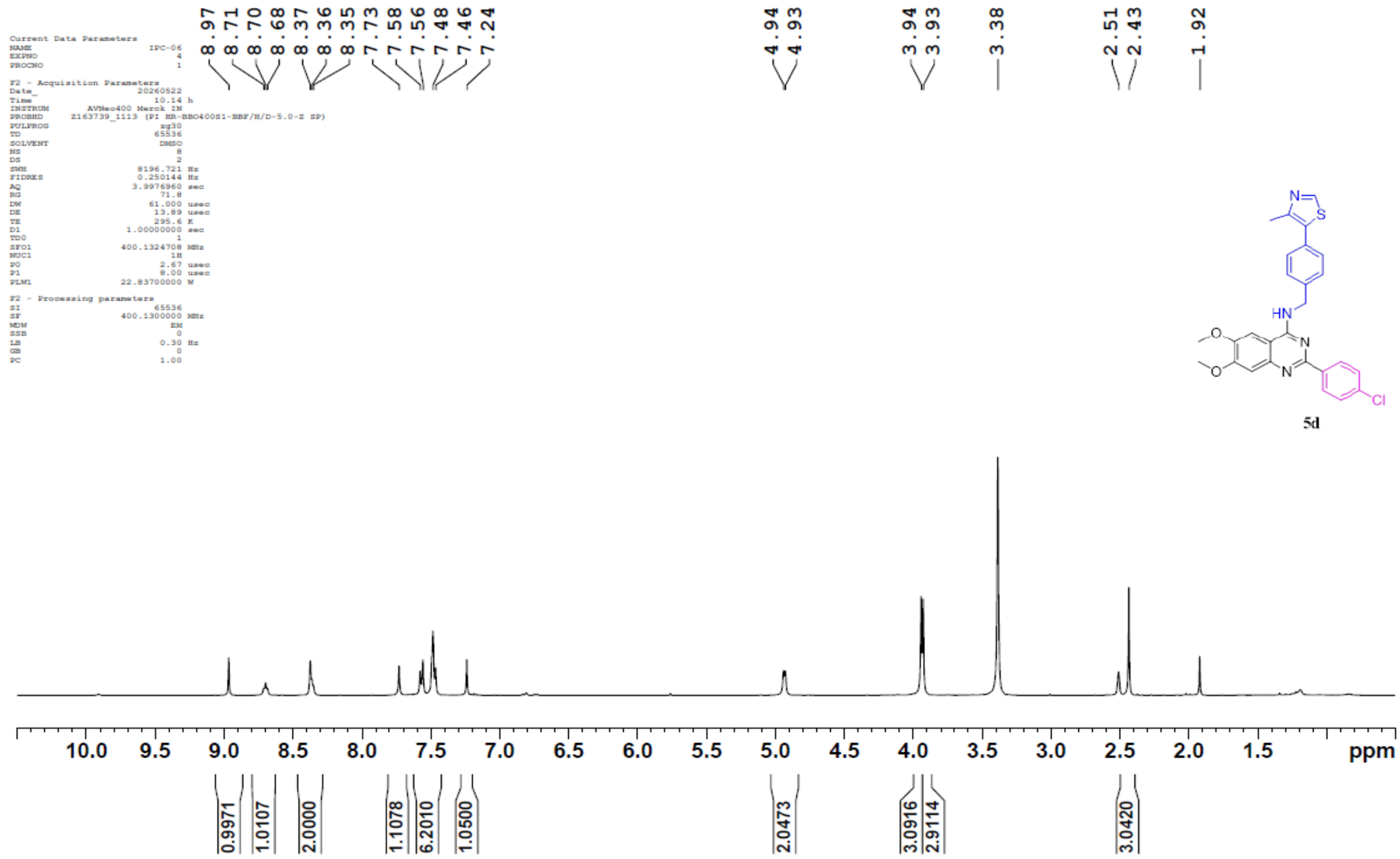

5d 13C NMR

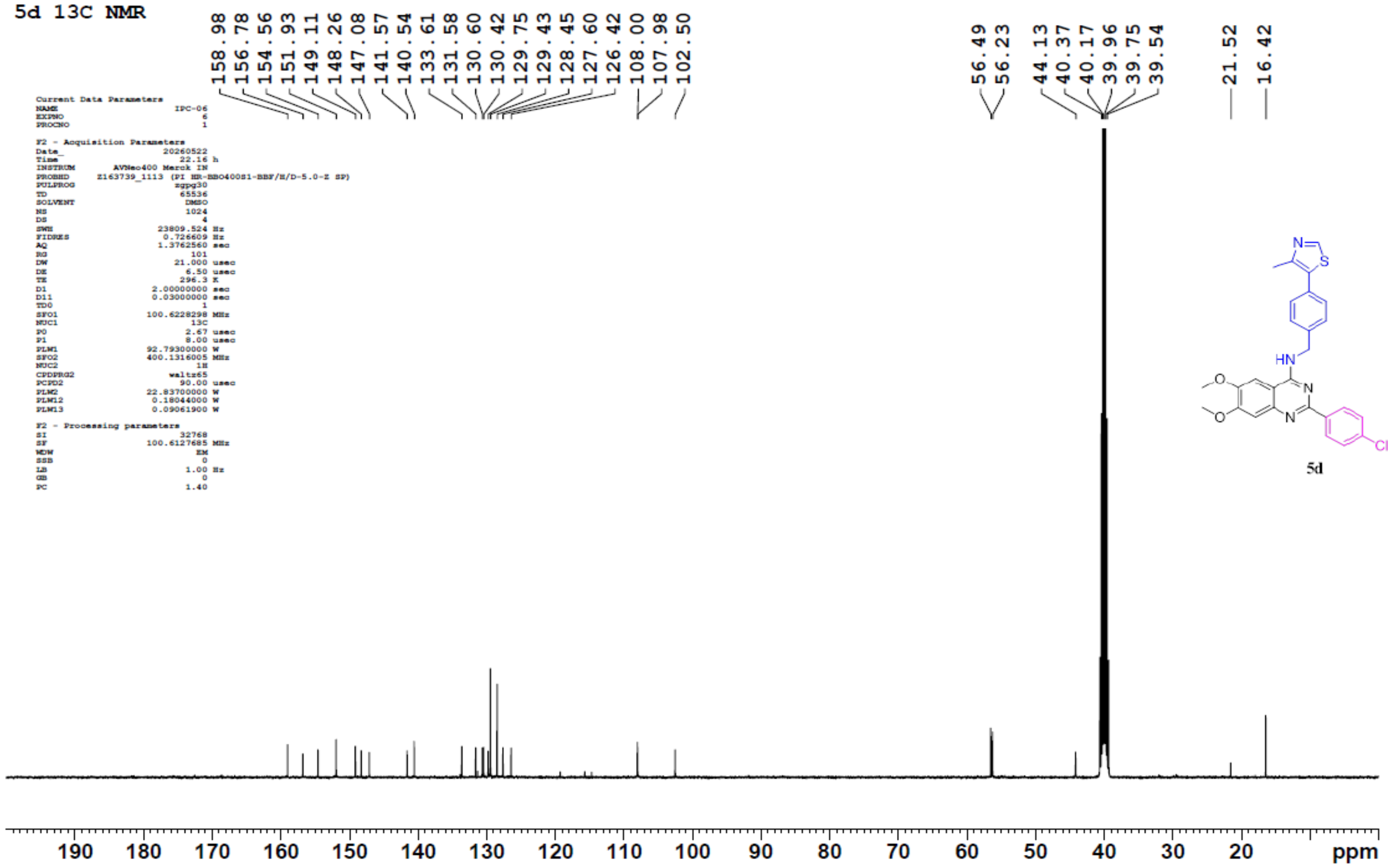

5e 1H NMR

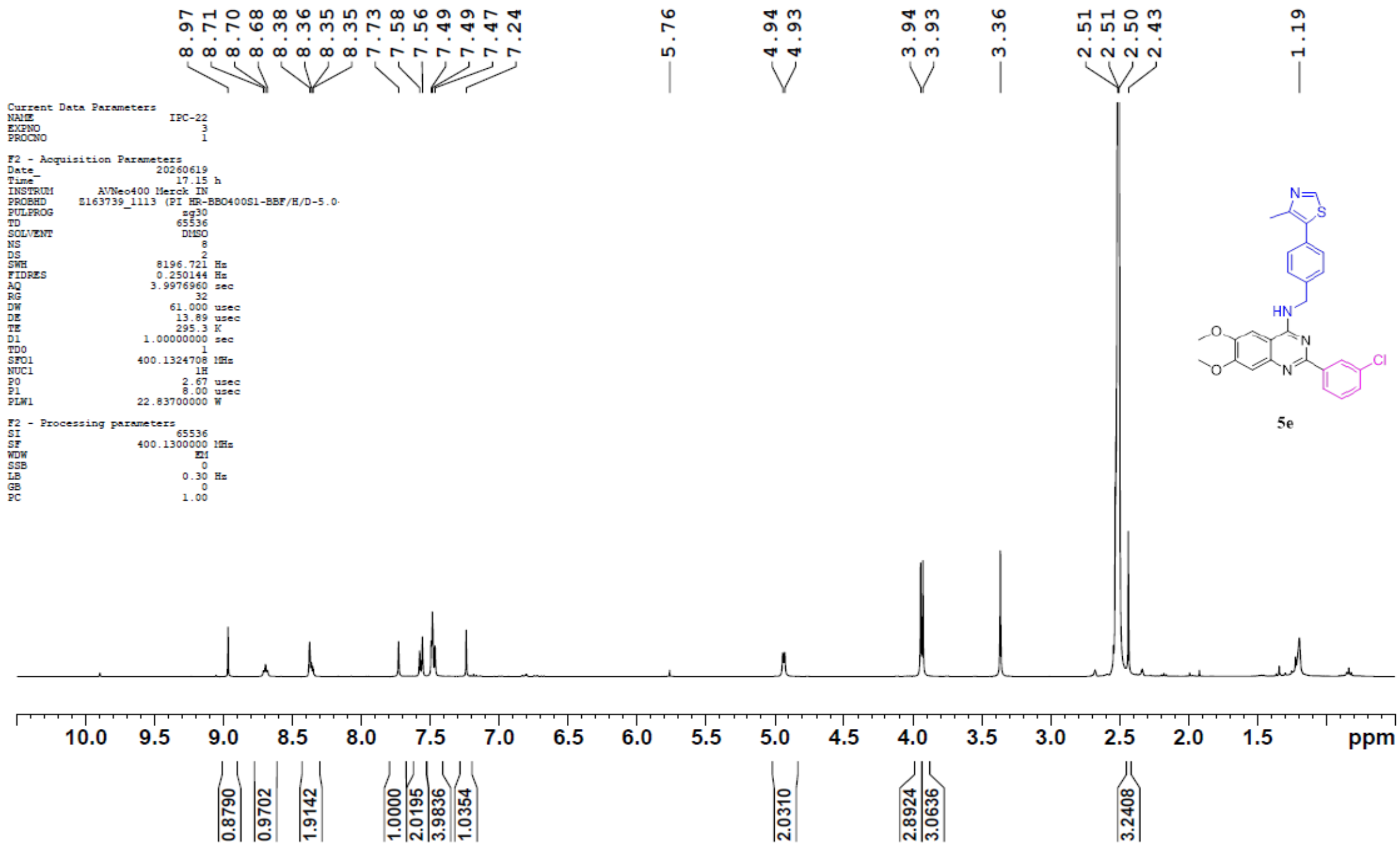

5e 13 NMR

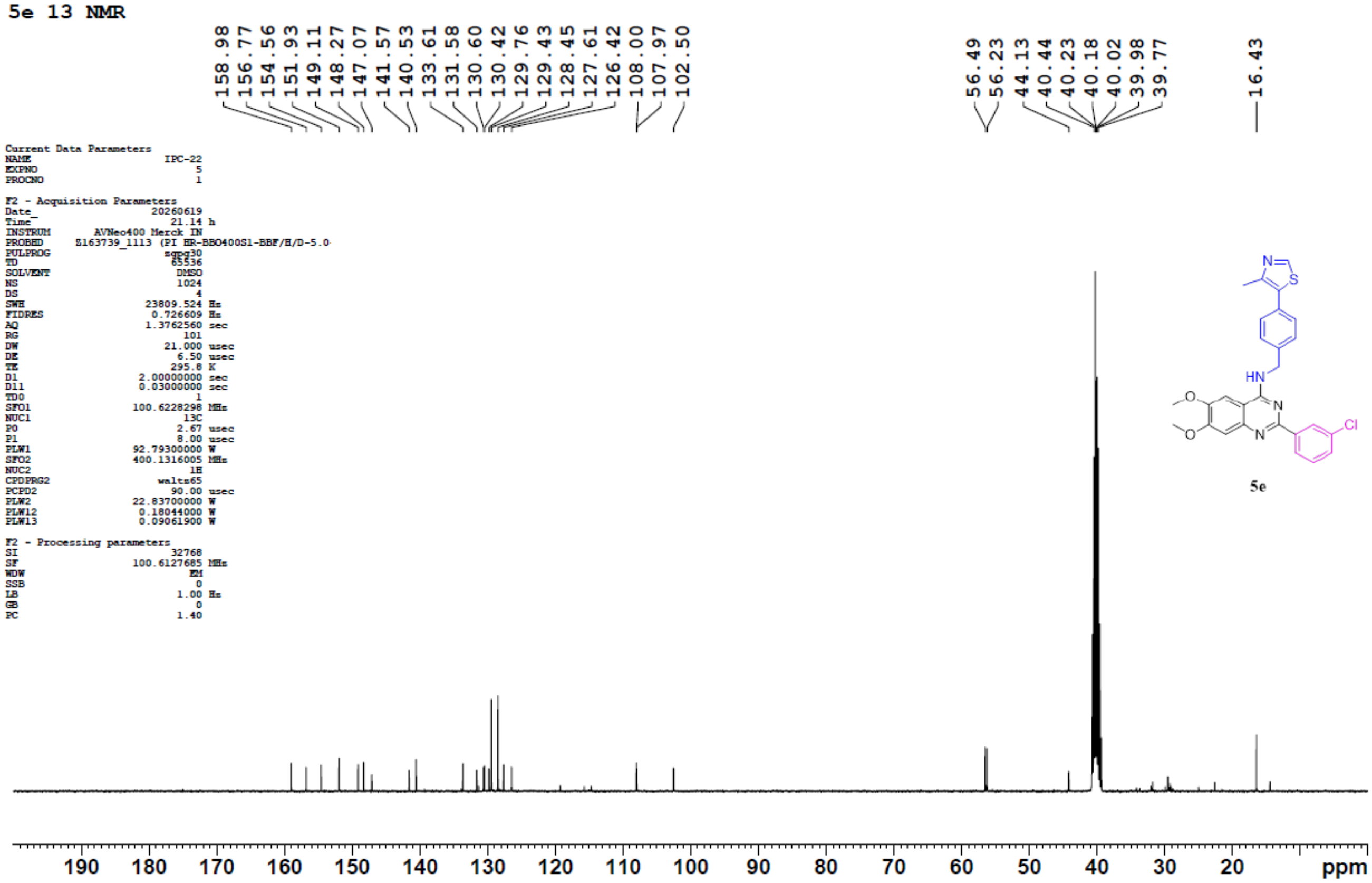

5f 1H NMR

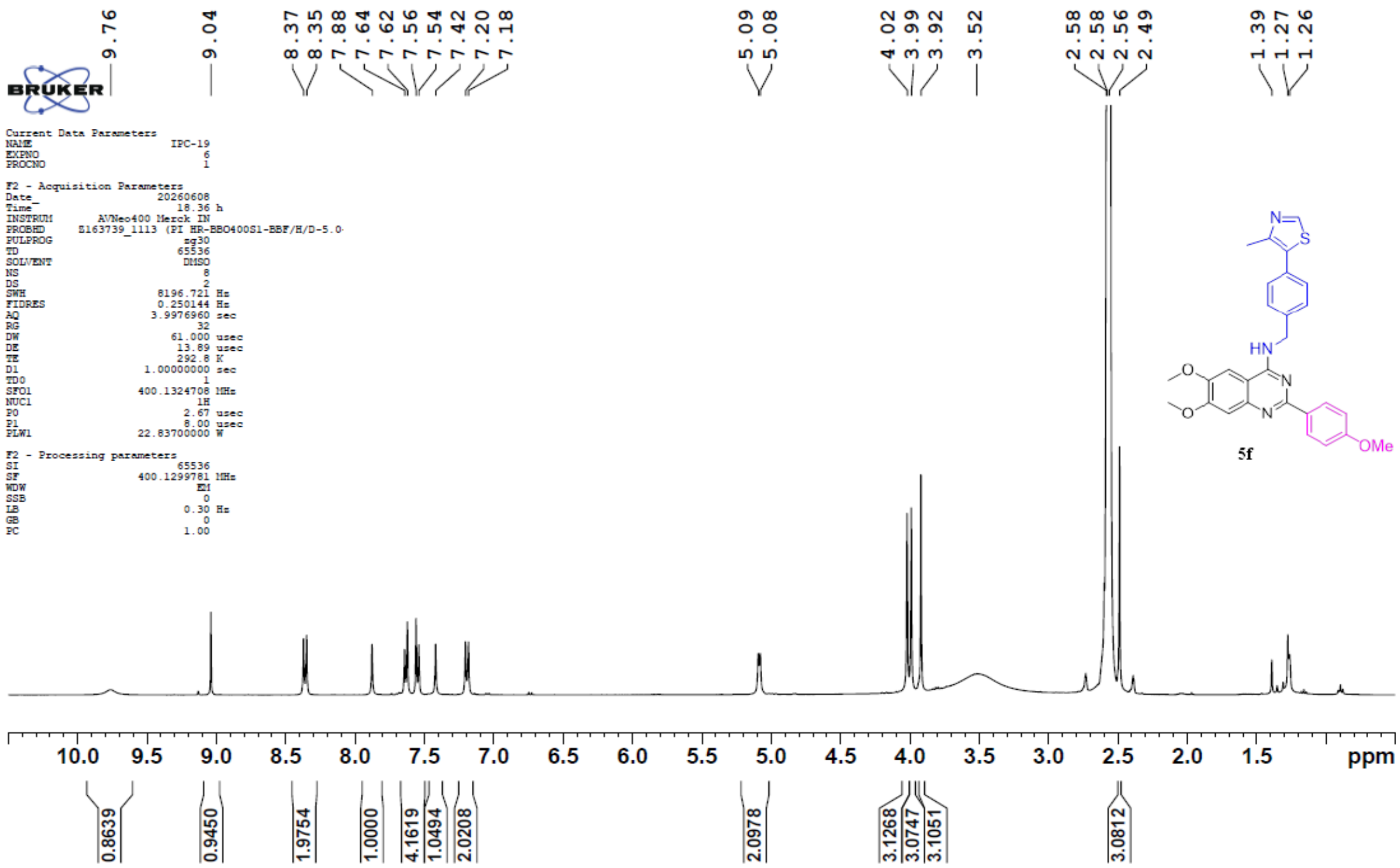

5f 13C NMR

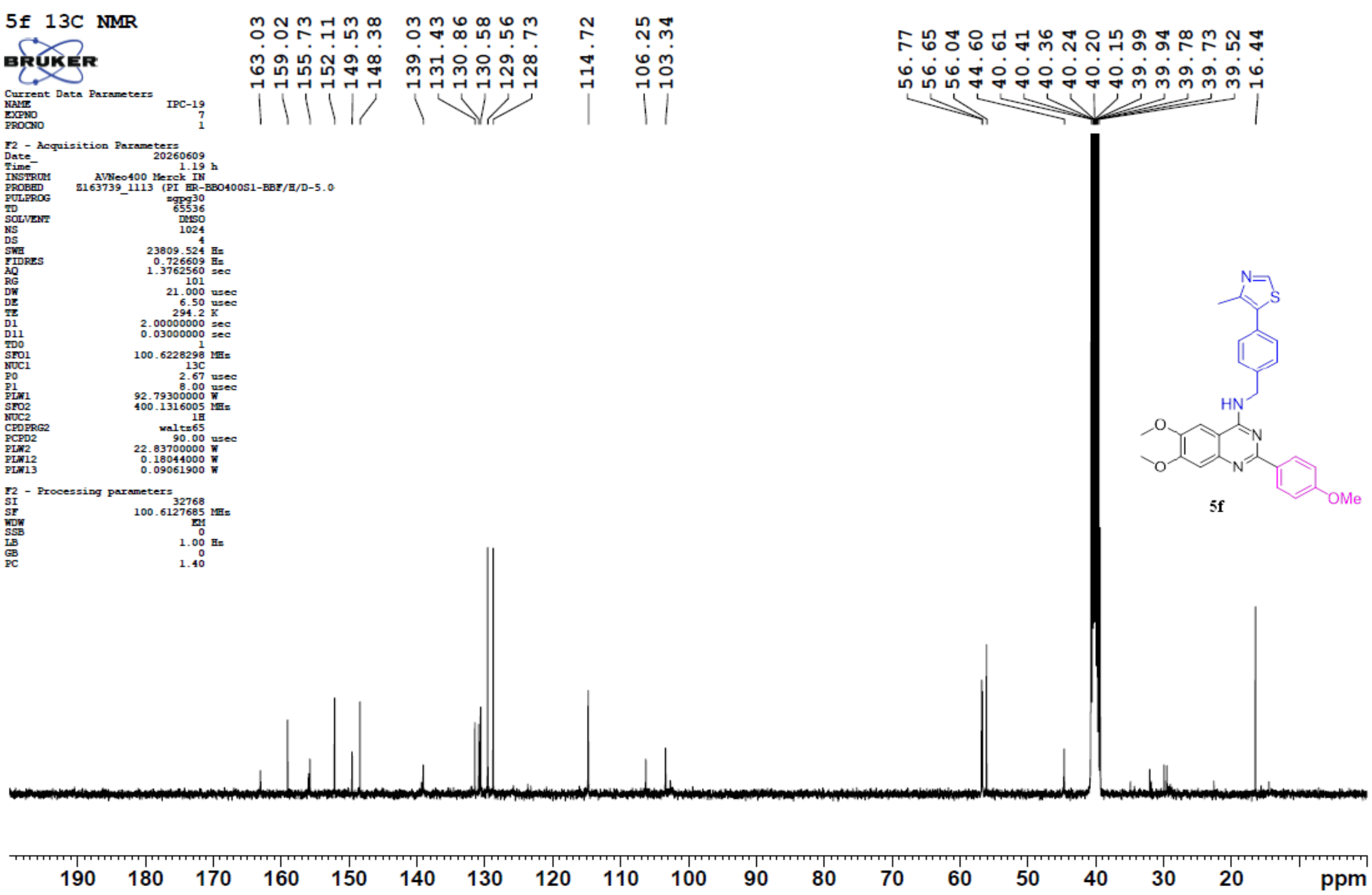

5g 1H NMR

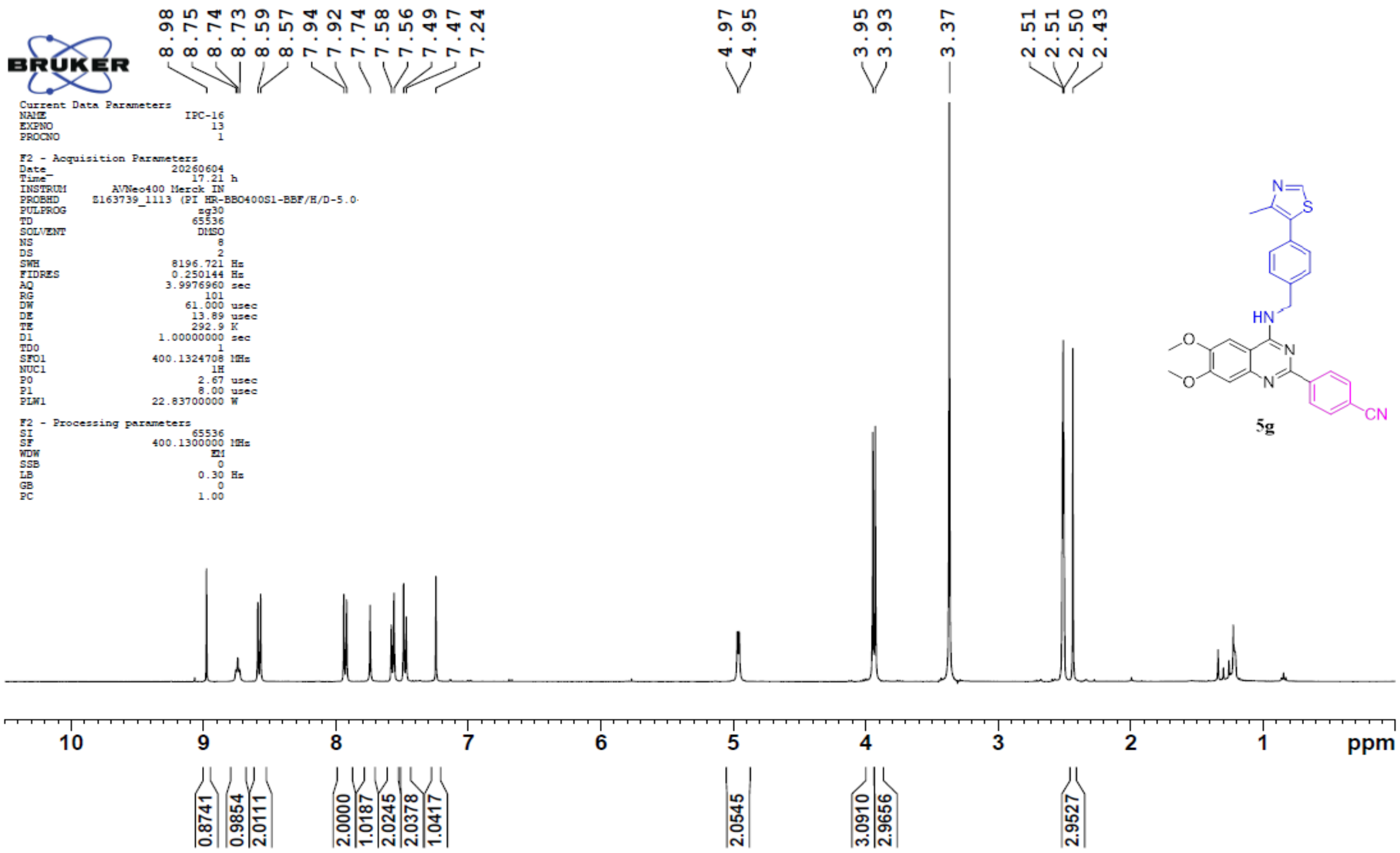

5g 13C NMR

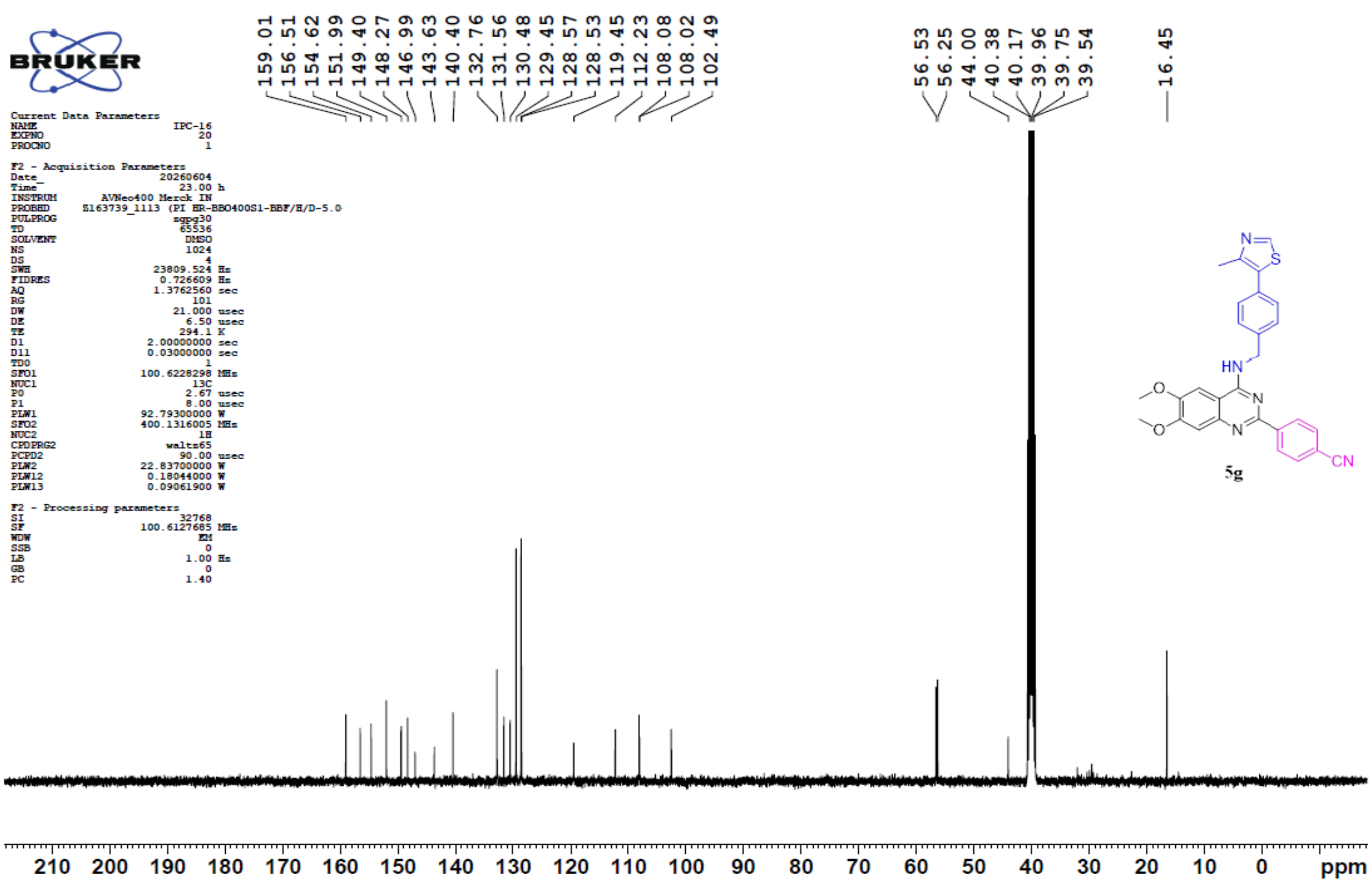

5h 1H NMR

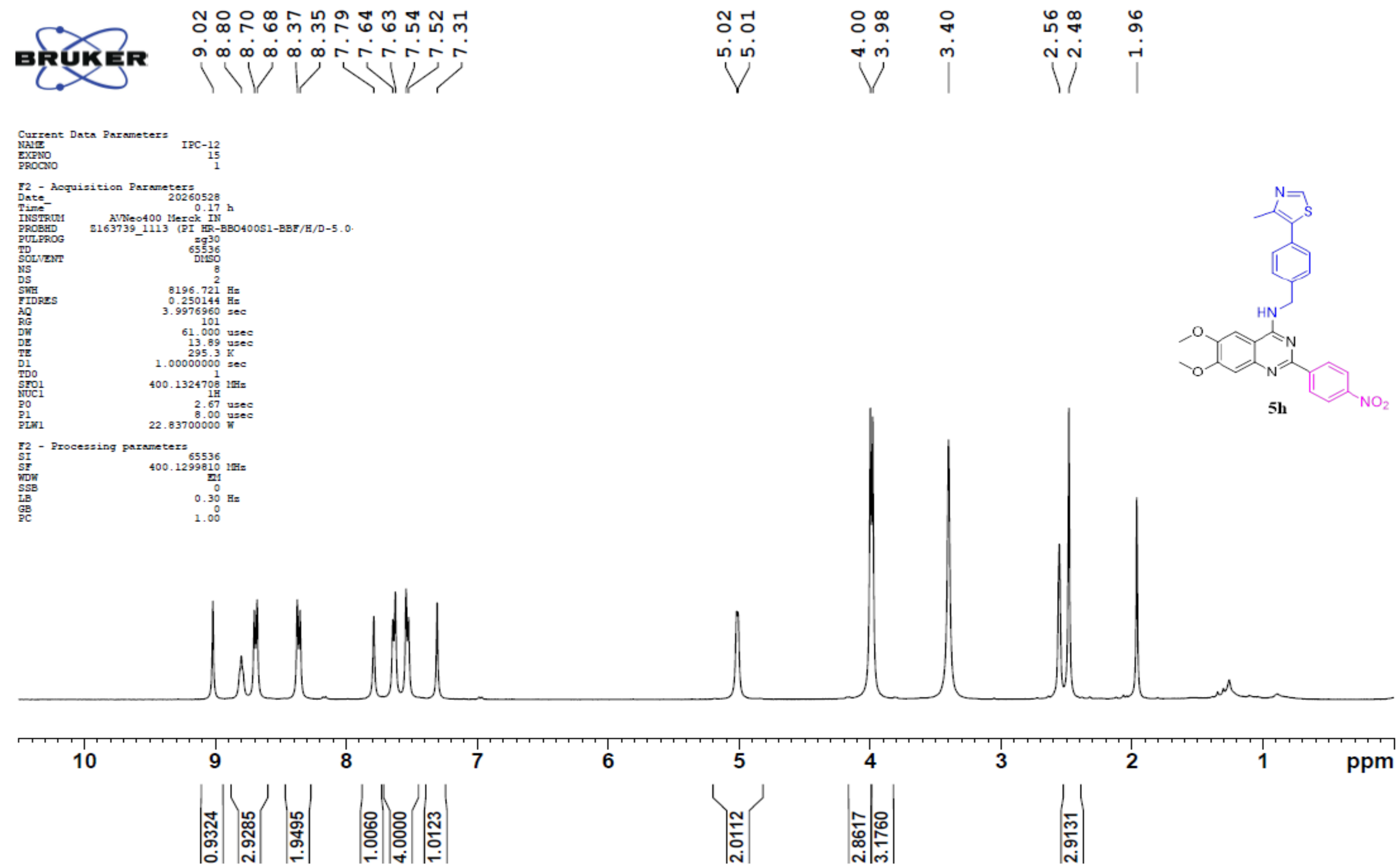

5h 13C NMR

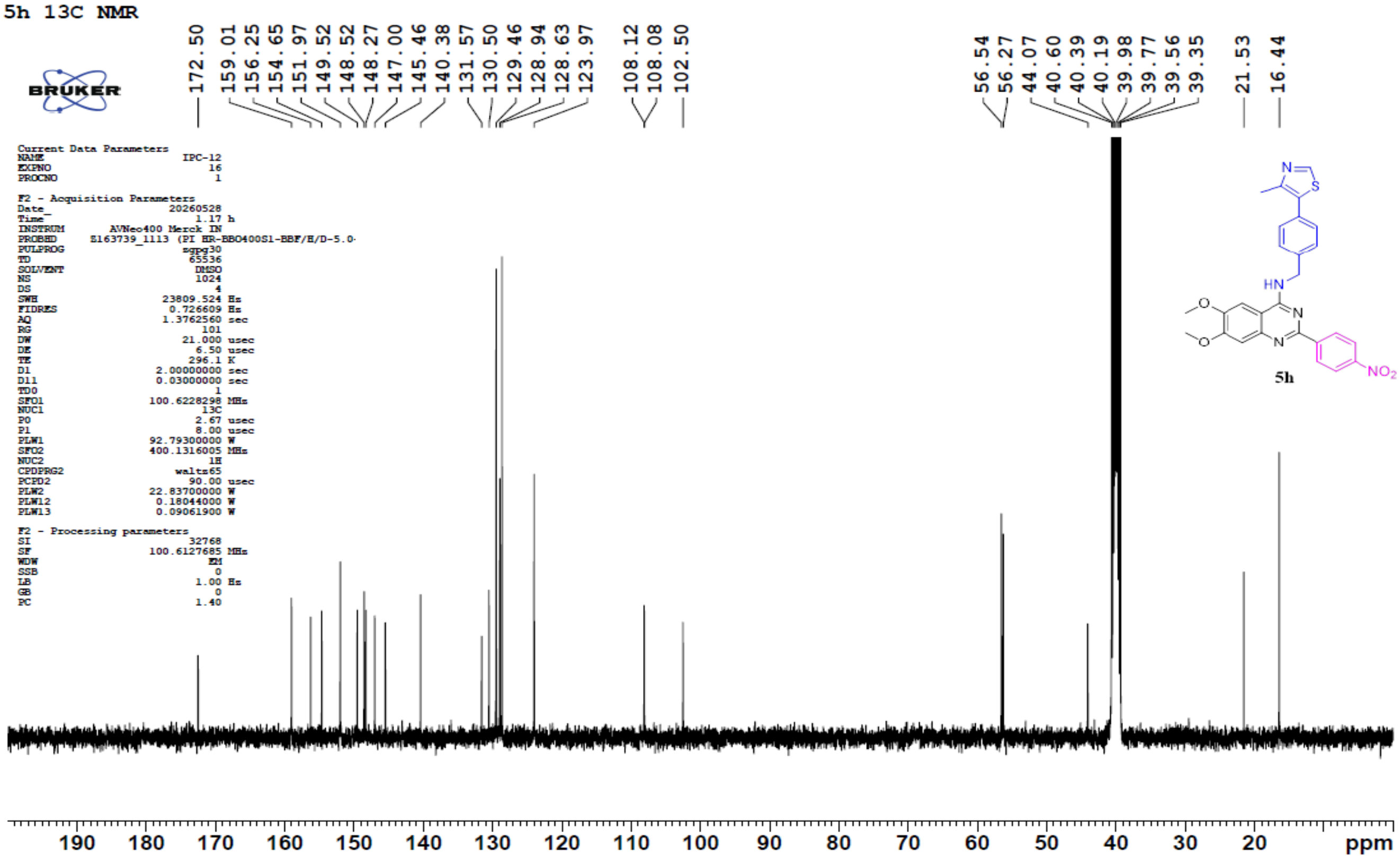

5i 1H NMR

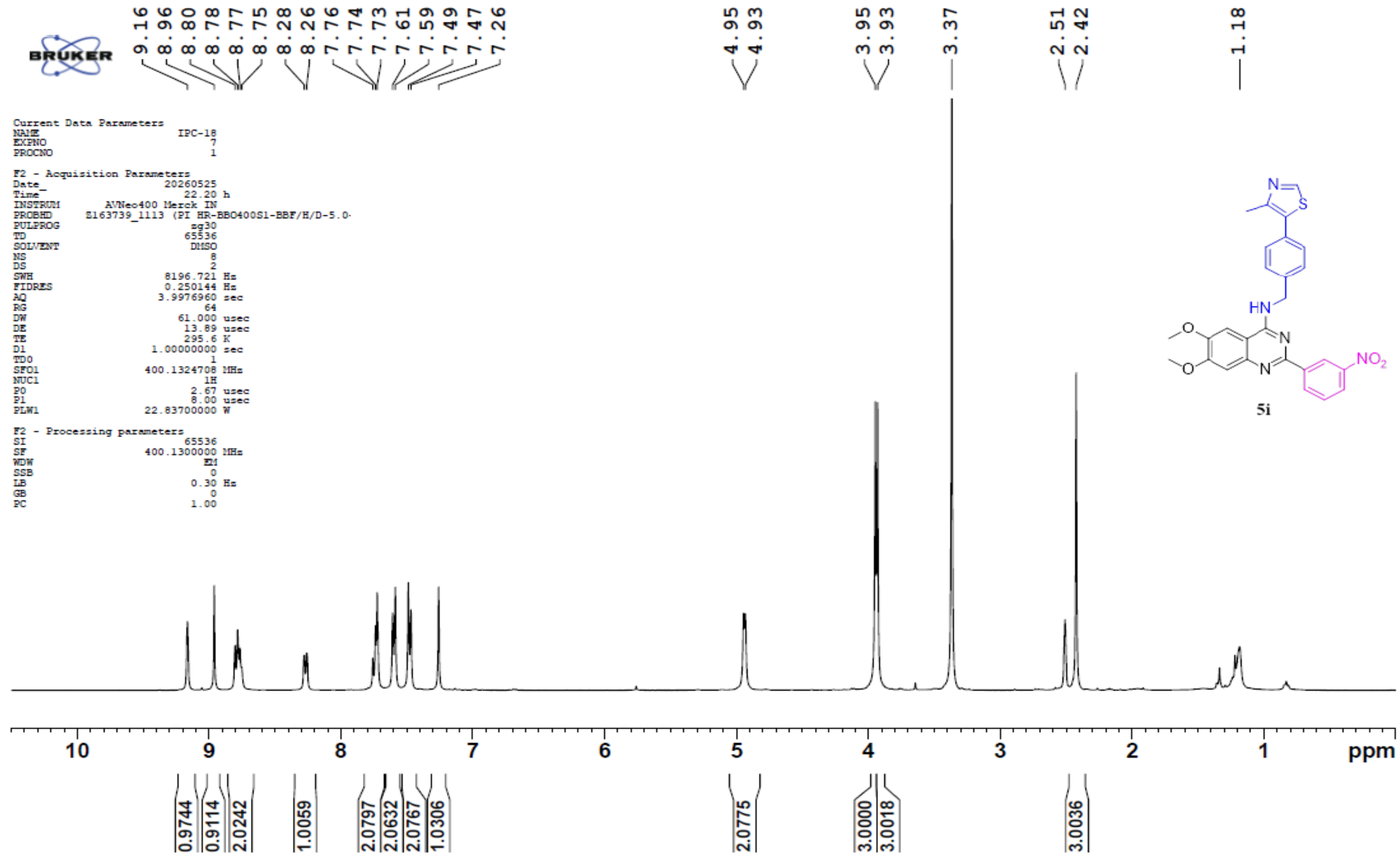

5i 13C NMR

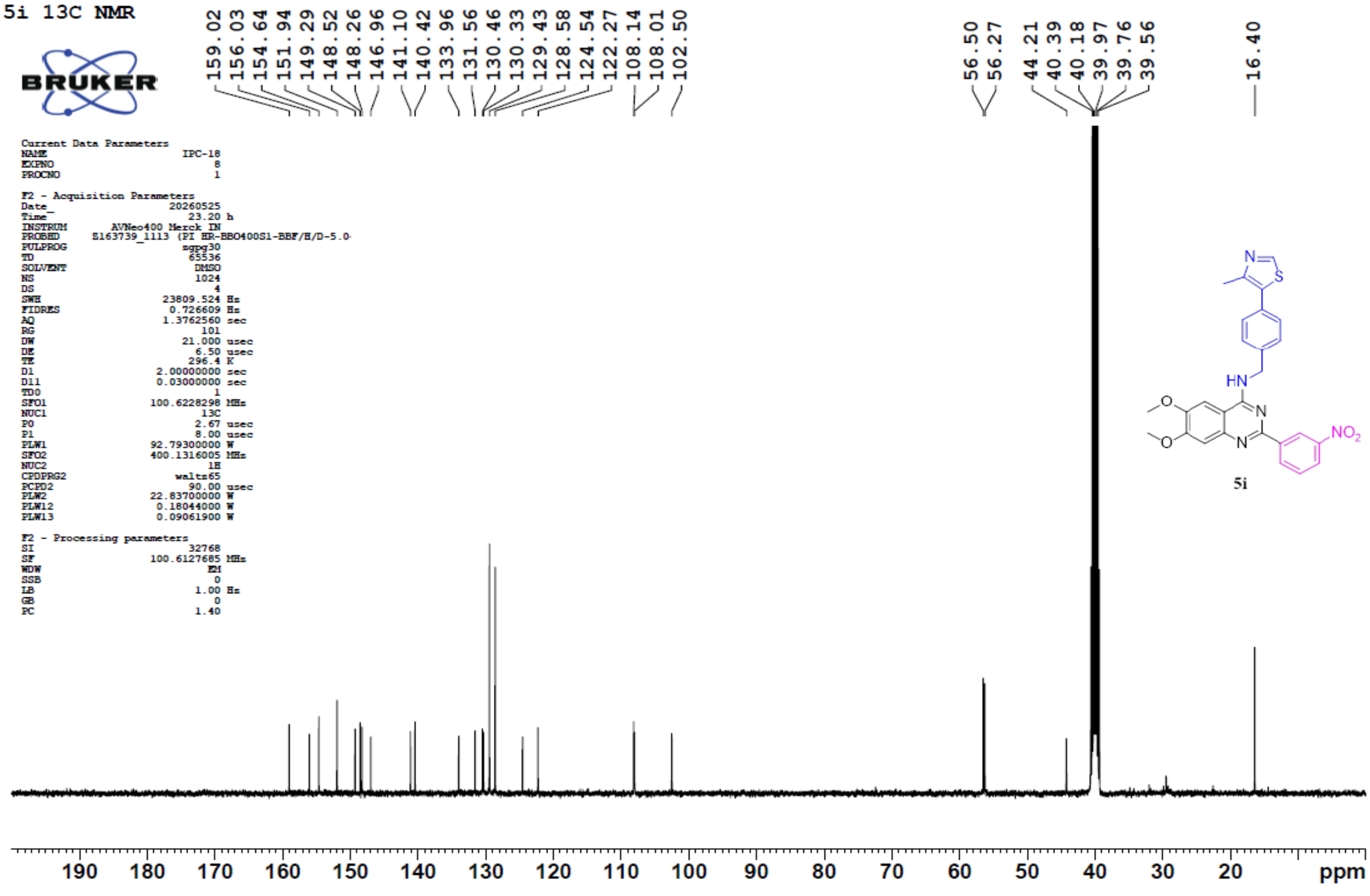

5j 1H NMR

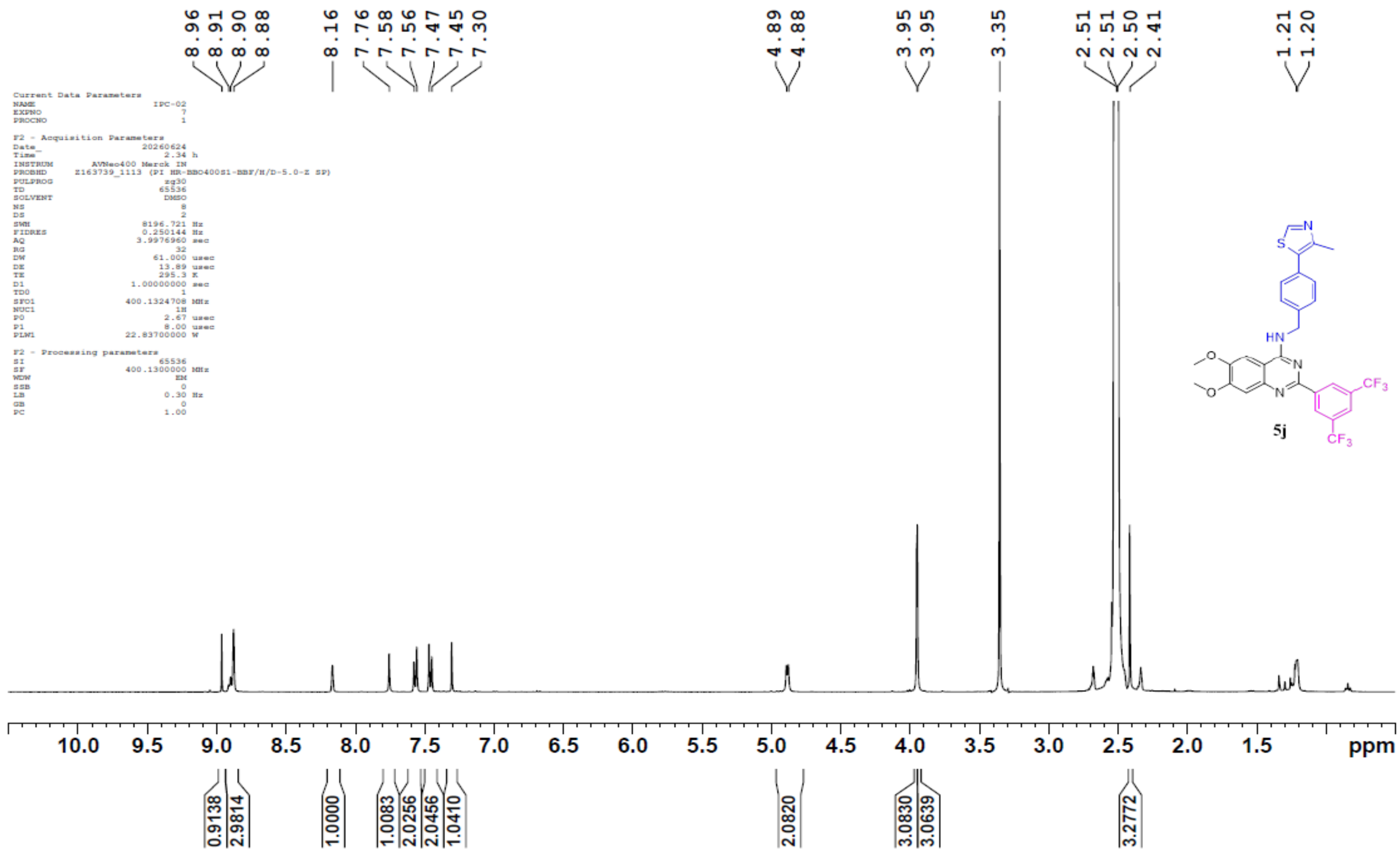

5j 13C NMR

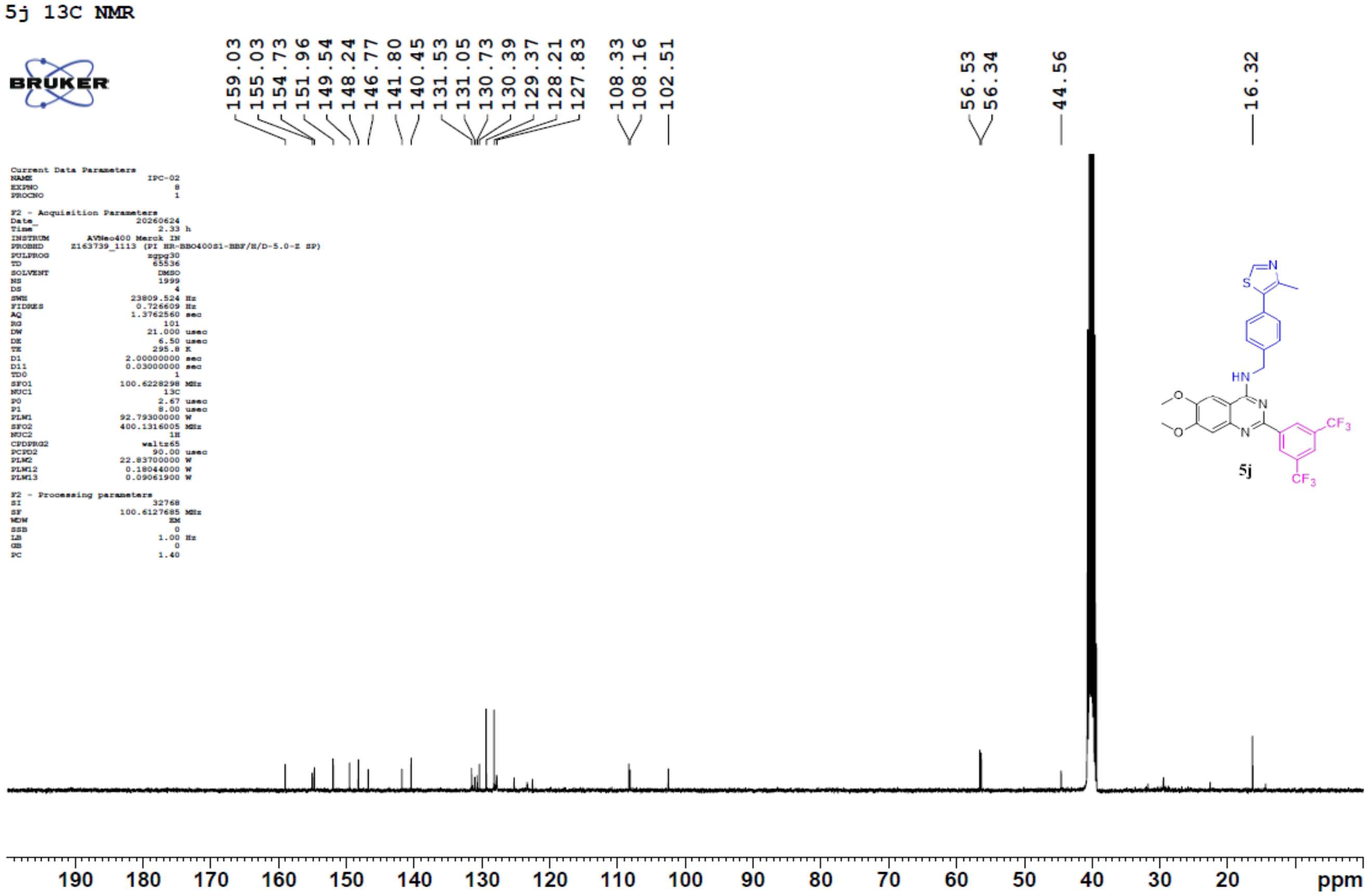

5k 1H NMR

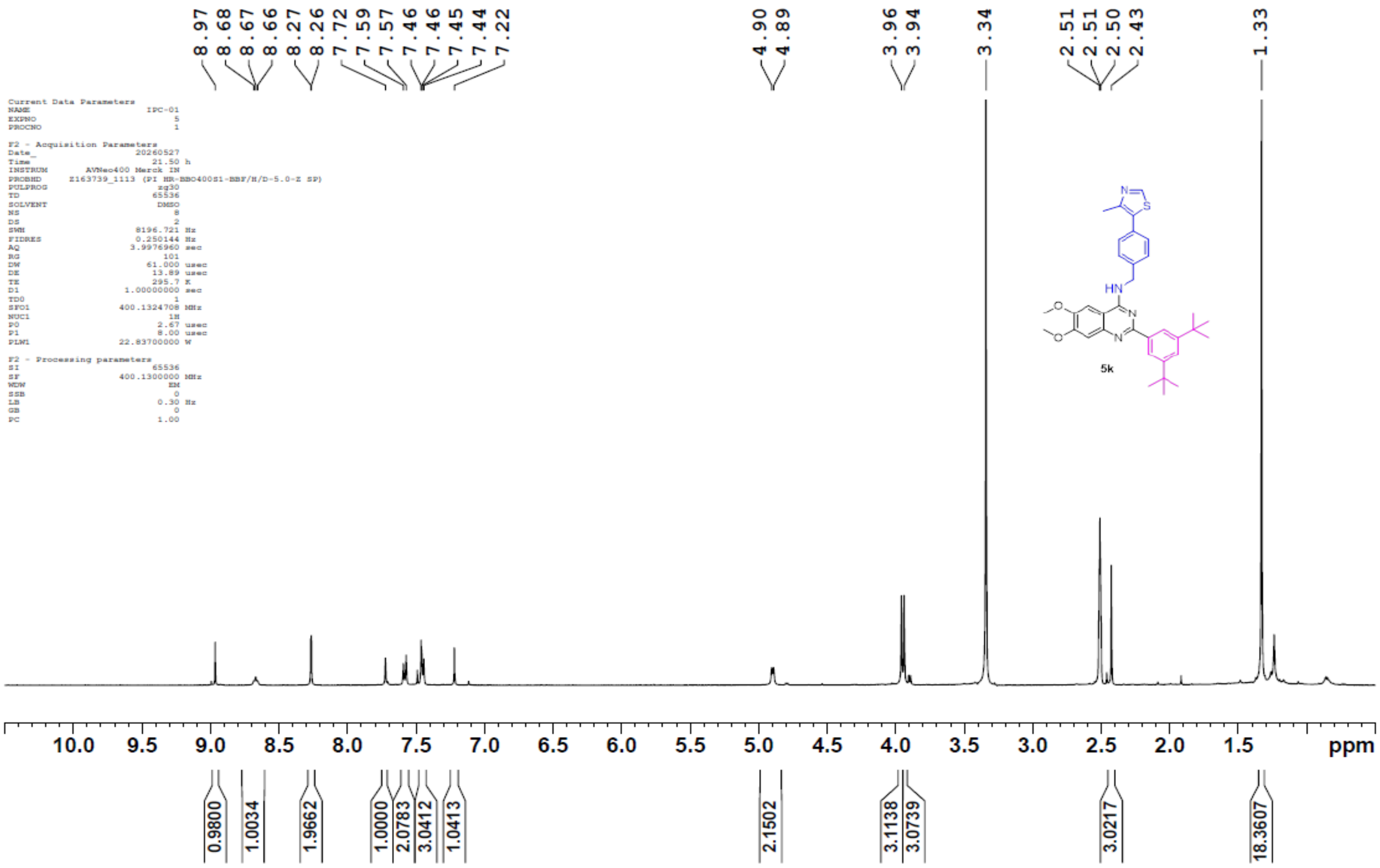

5k 13C NMR

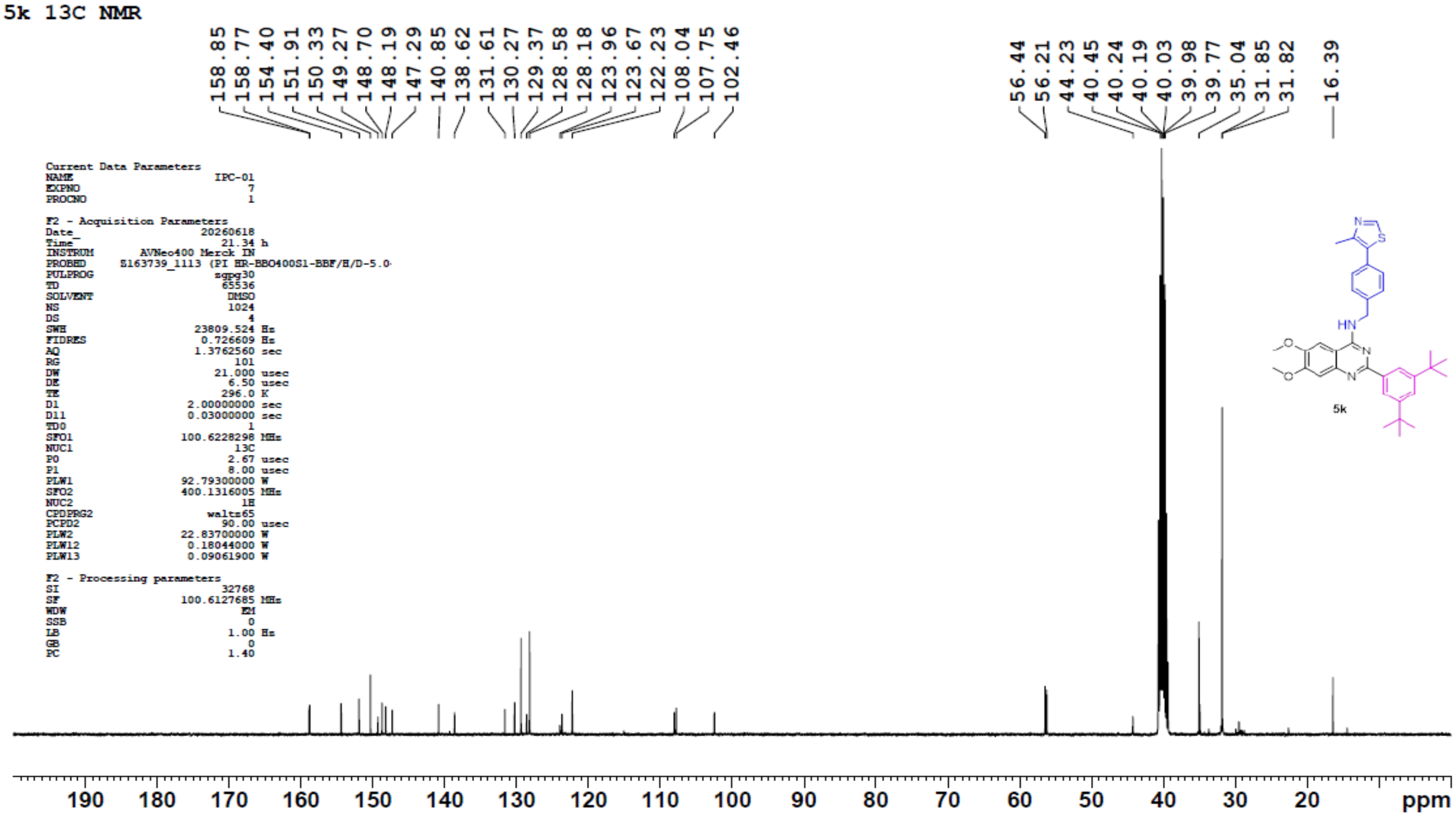

51 1H NMR

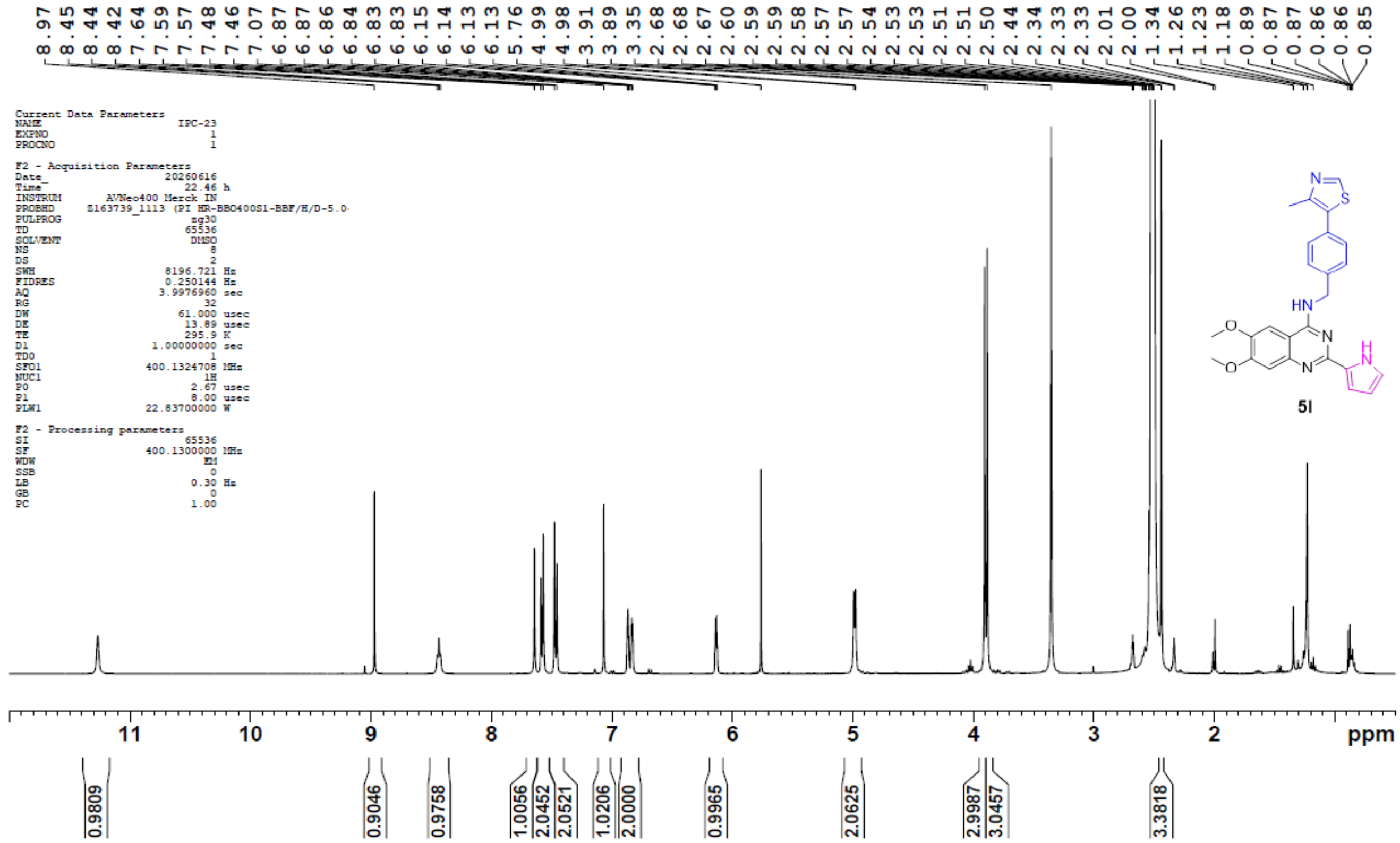

51 13C NMR

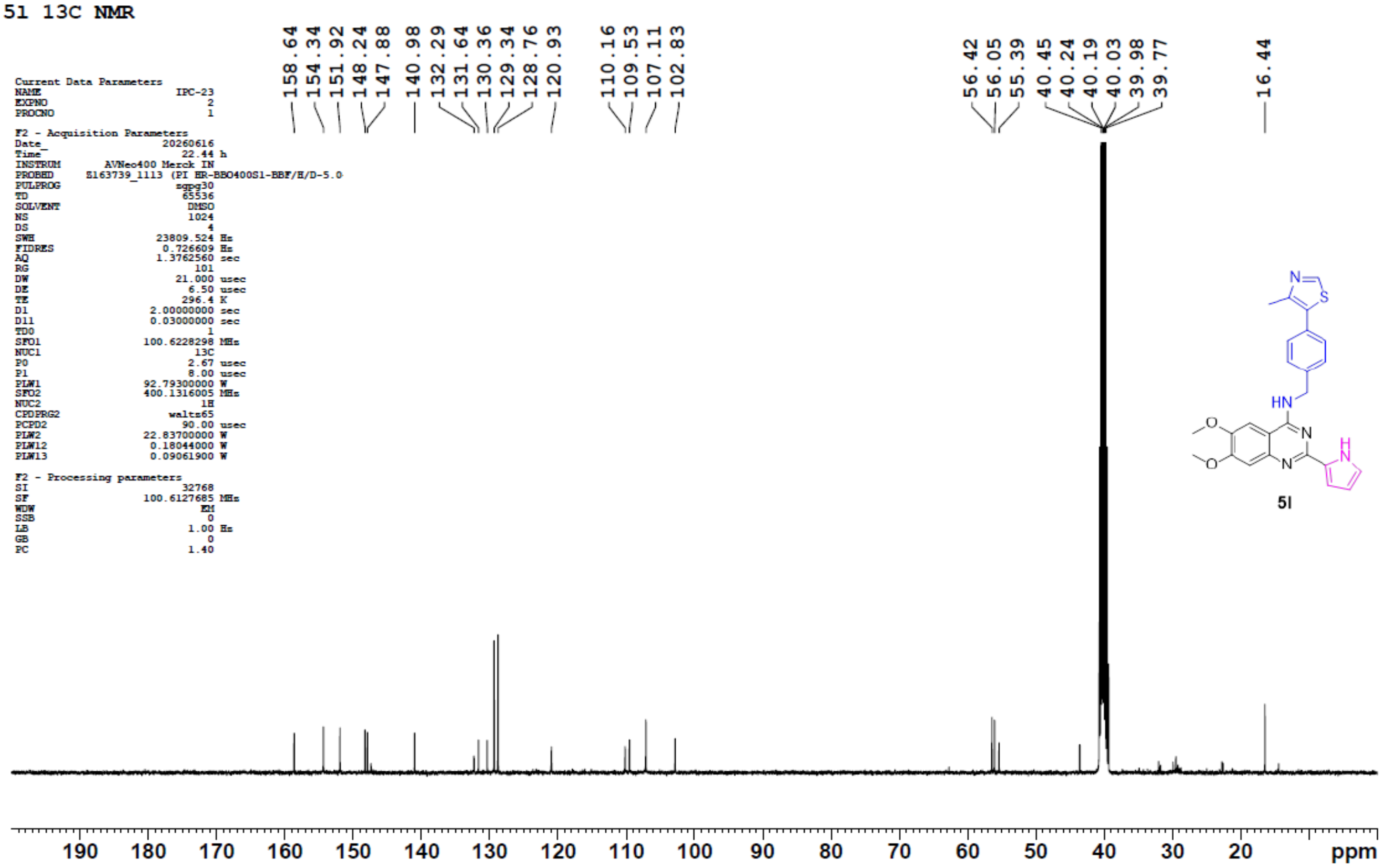

5m 1H NMR

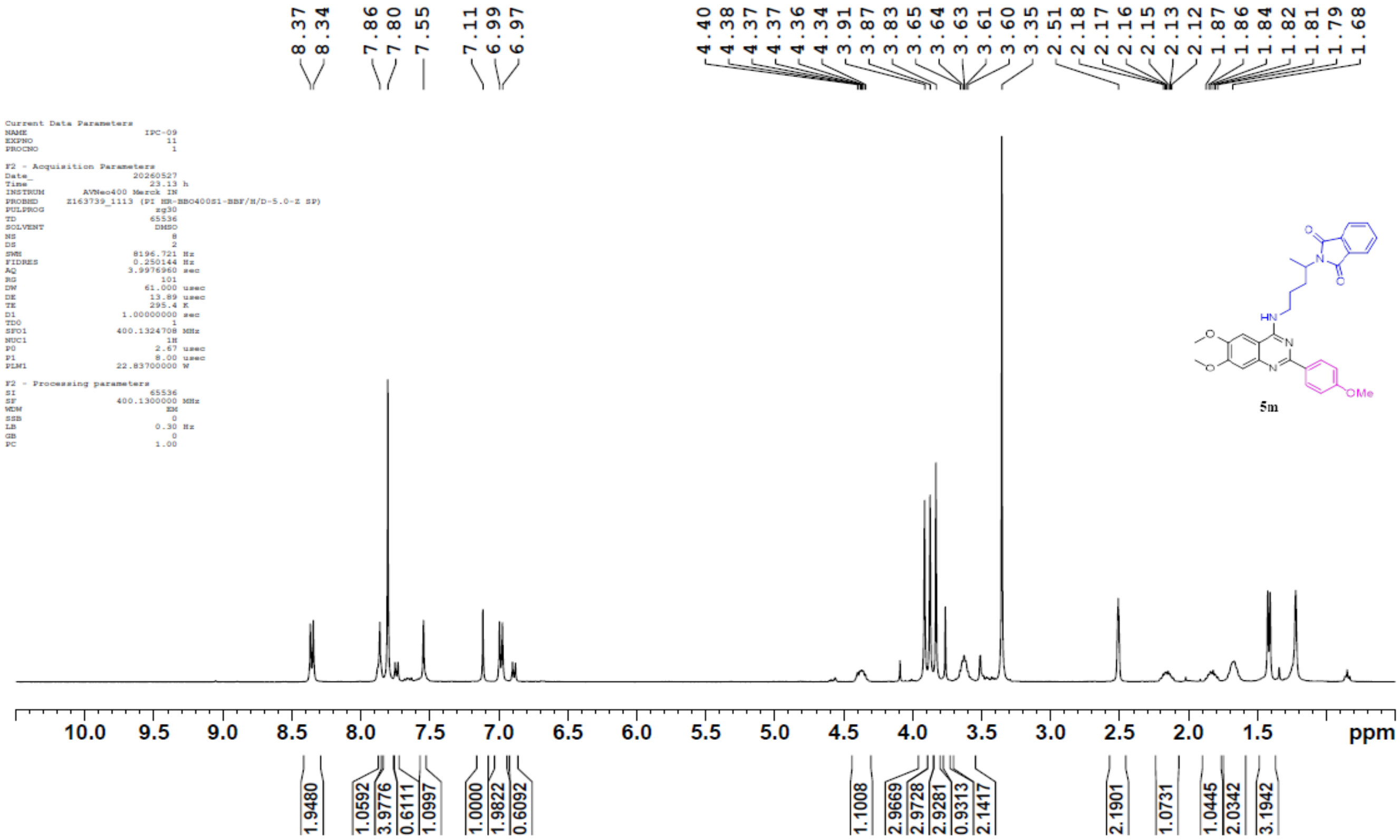

5m 13C NMR

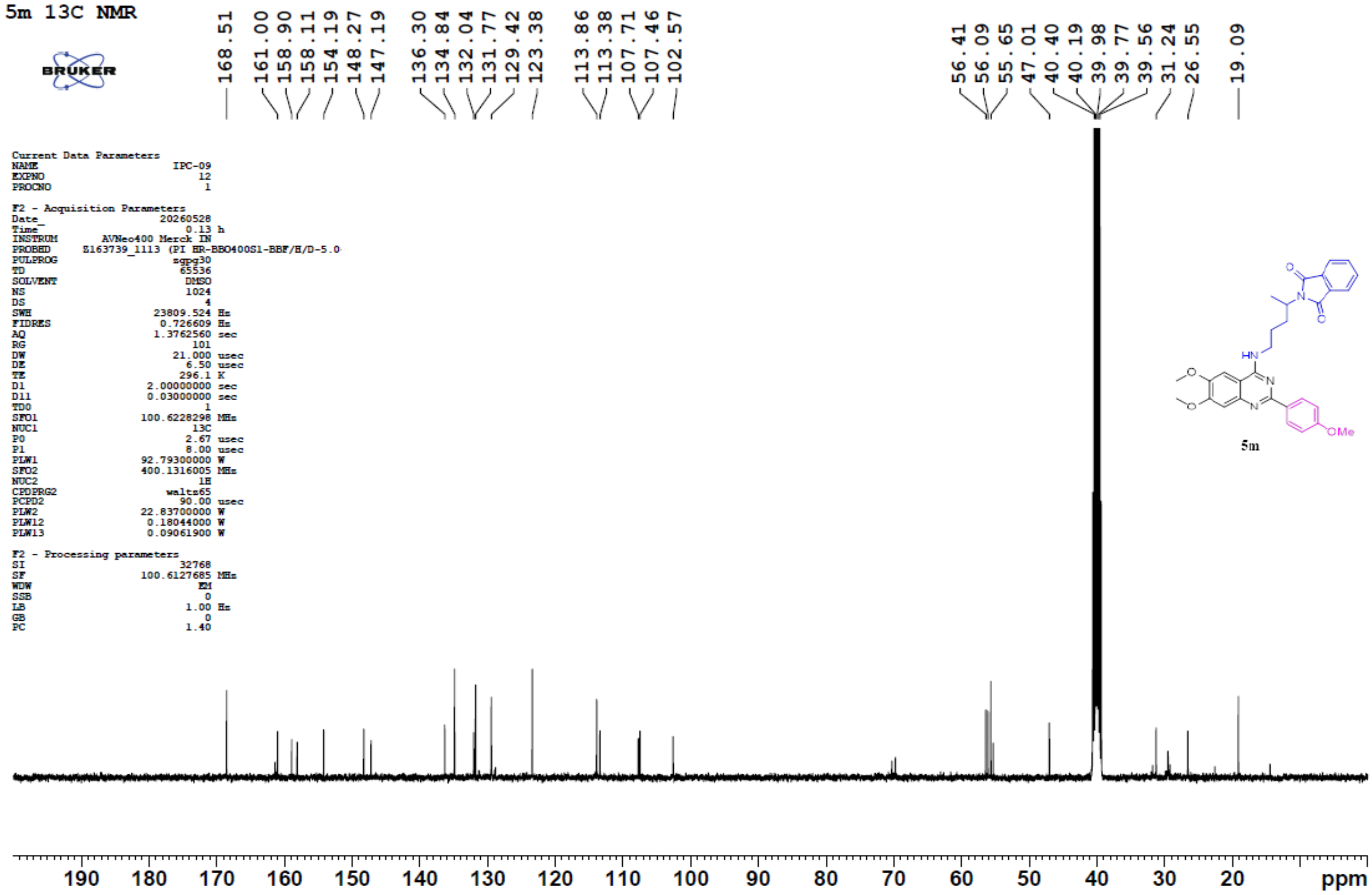

5n 1H NMR

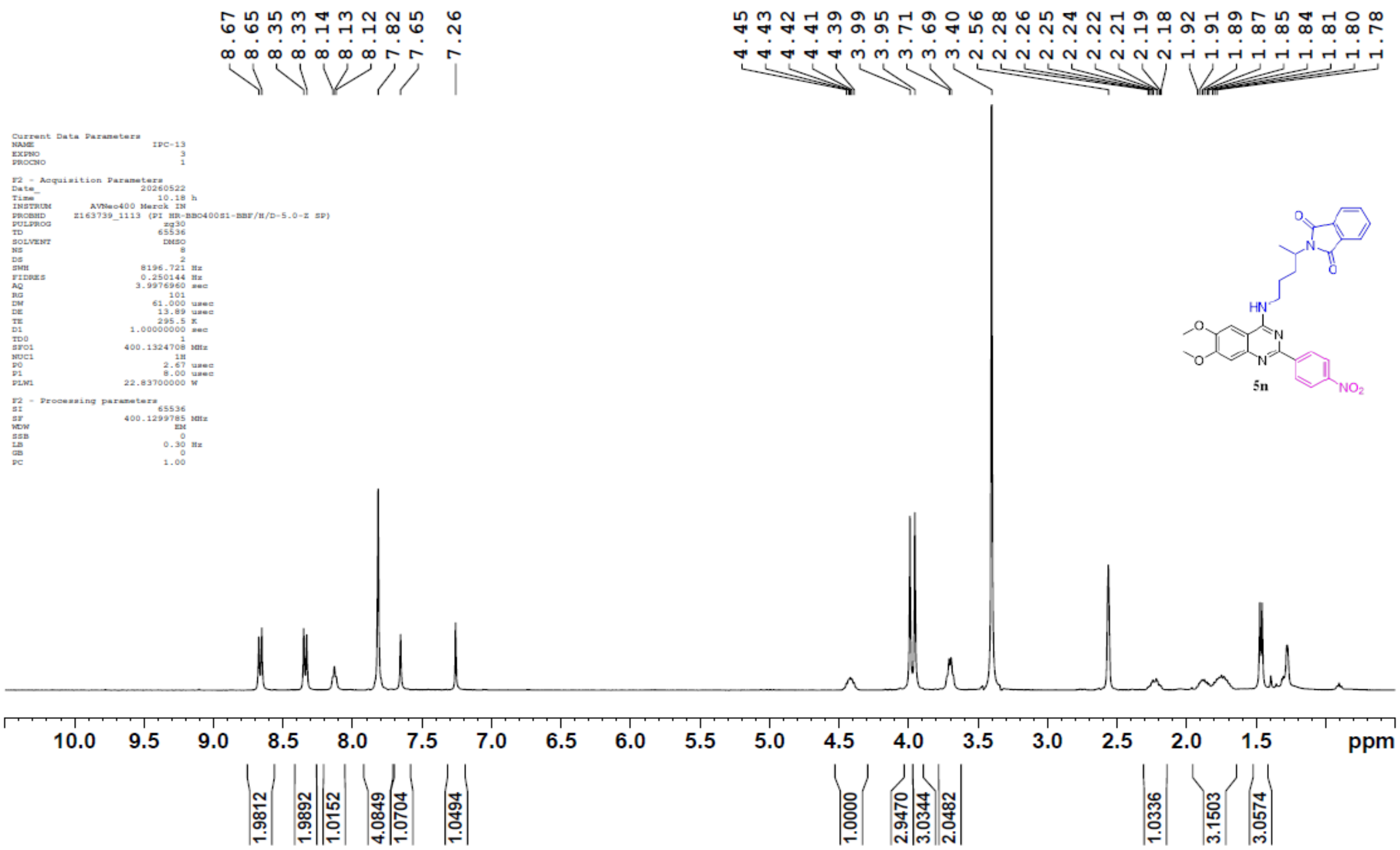

5n 13C NMR

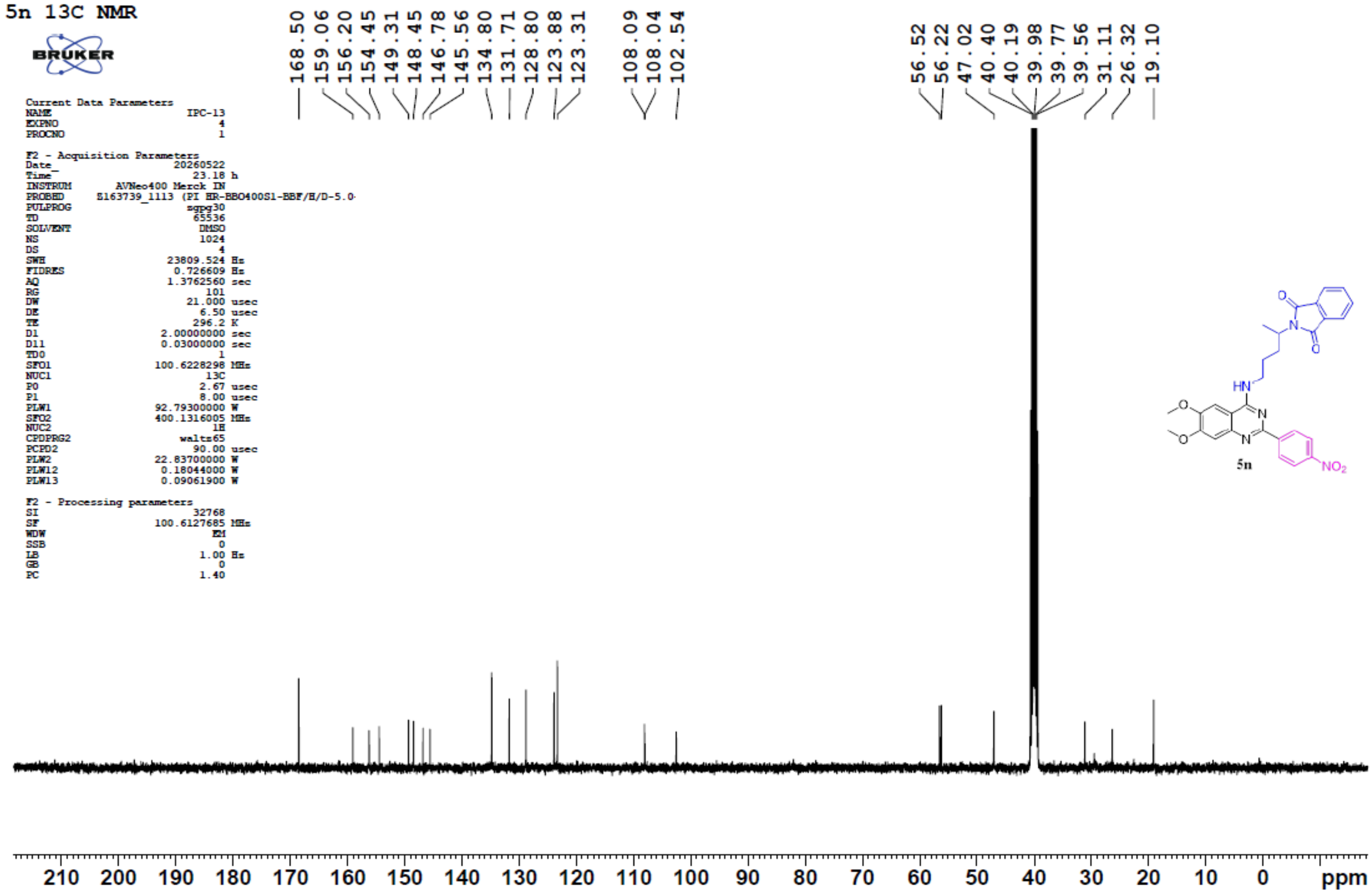

5o 1H NMR

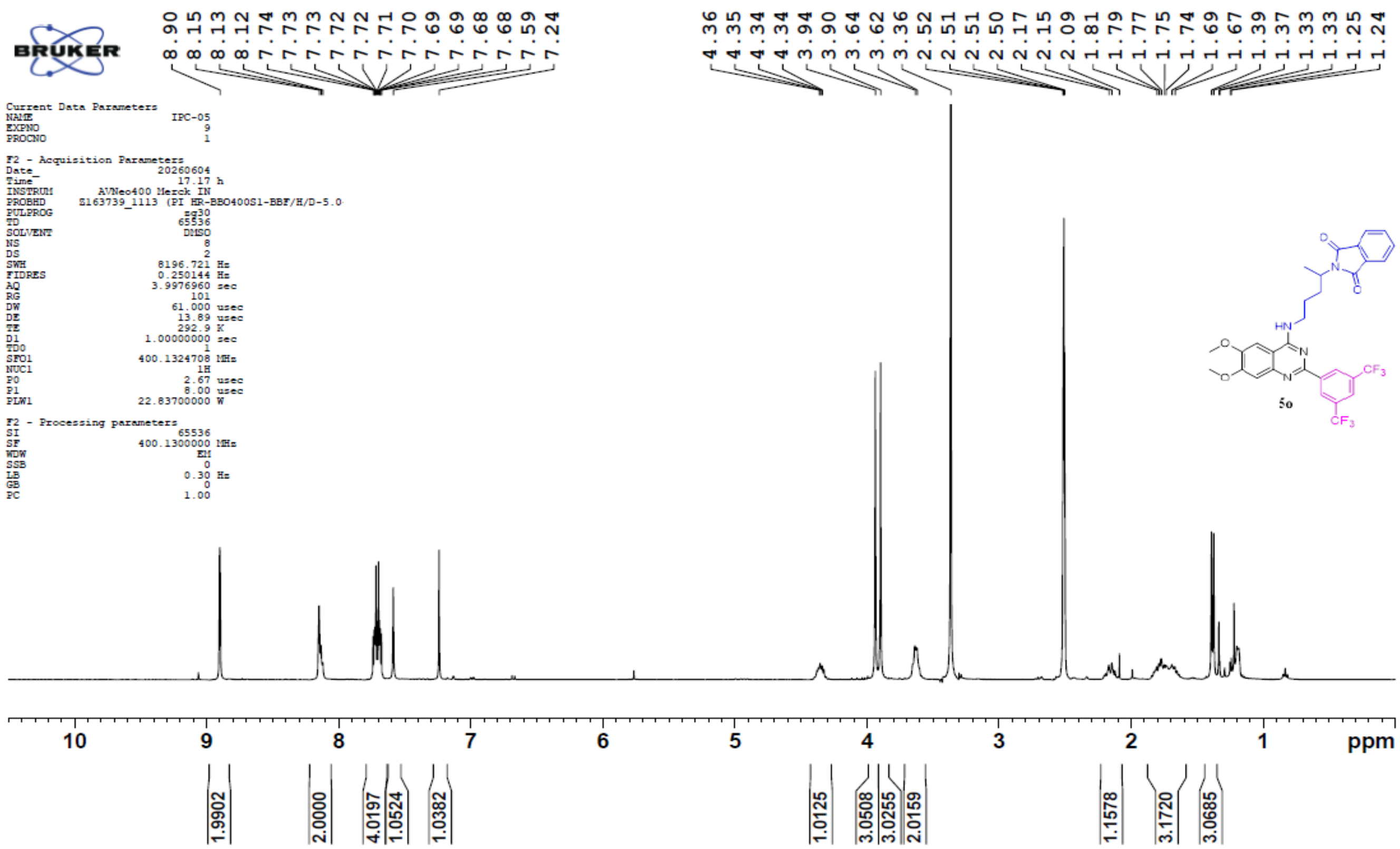

5o 13C NMR

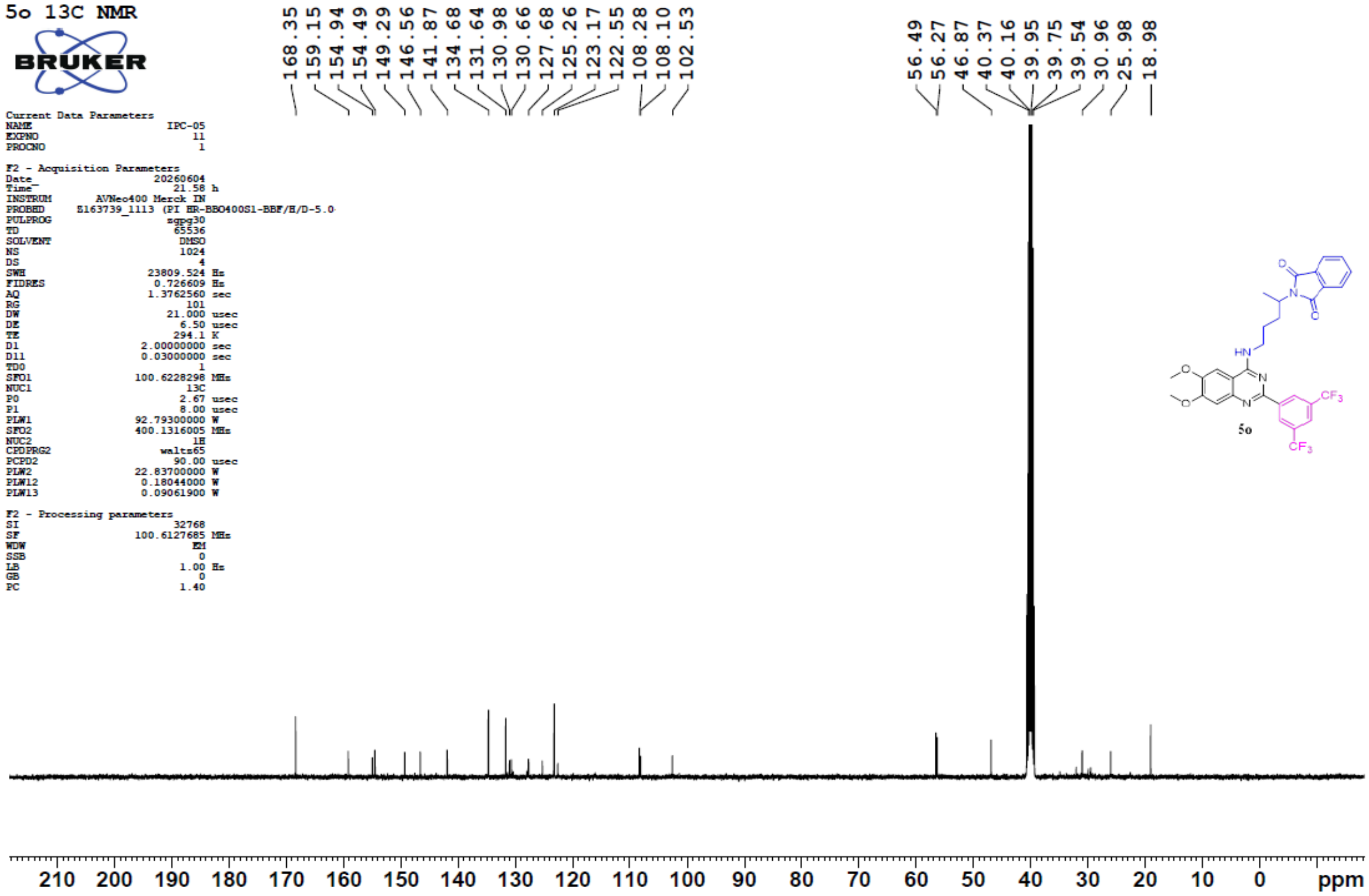

5p 1H NMR

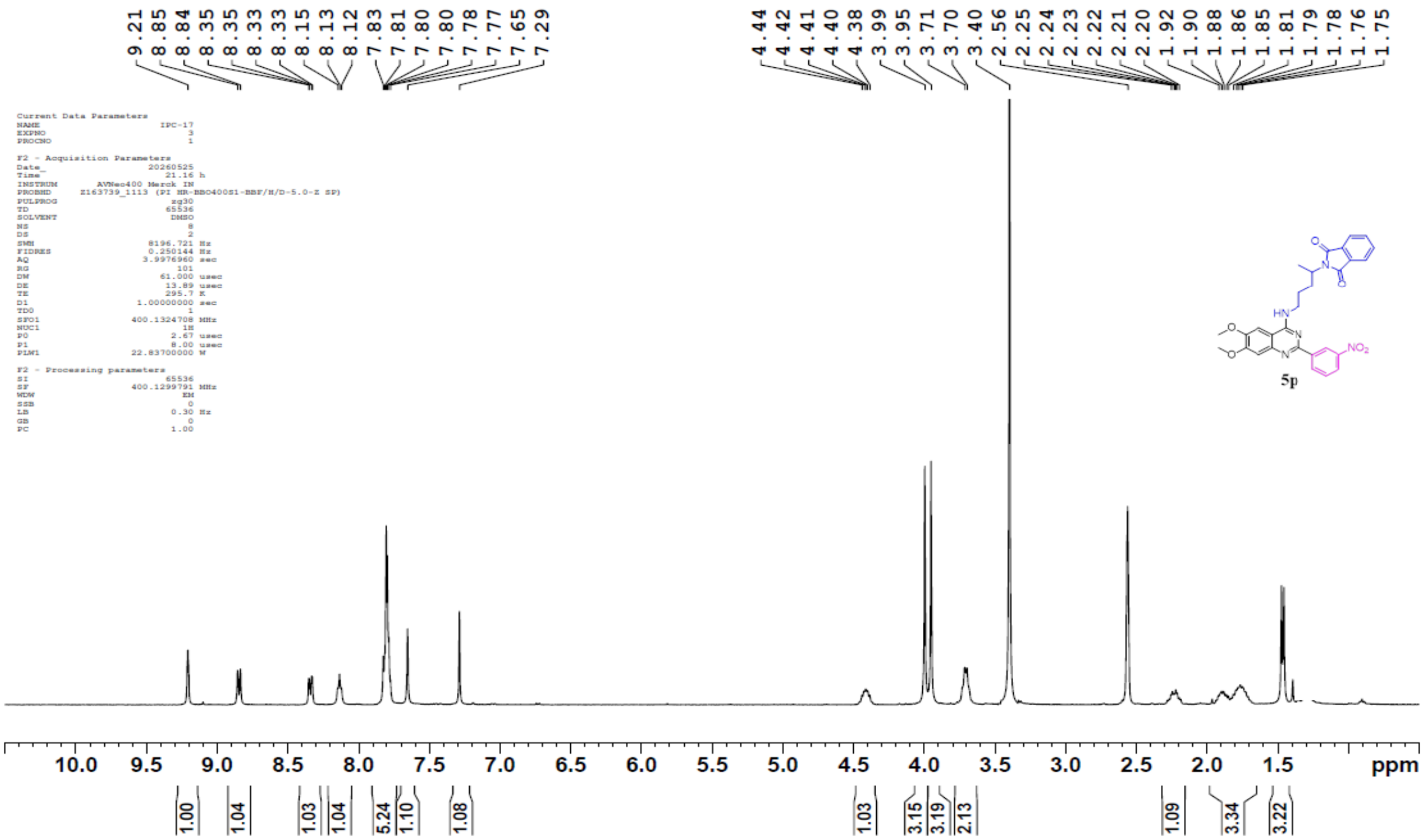

5p 13C NMR

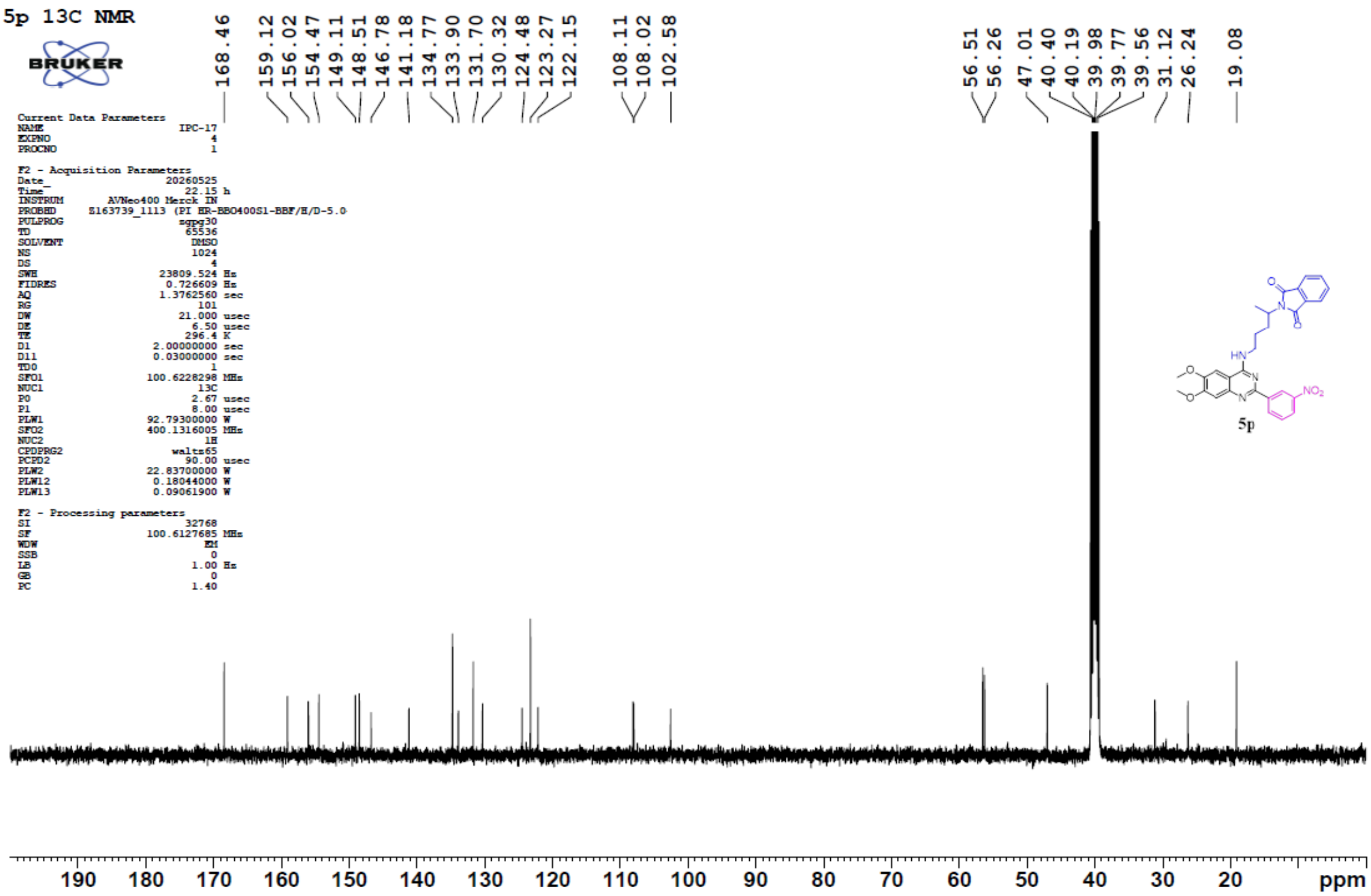

5q 1H NMR

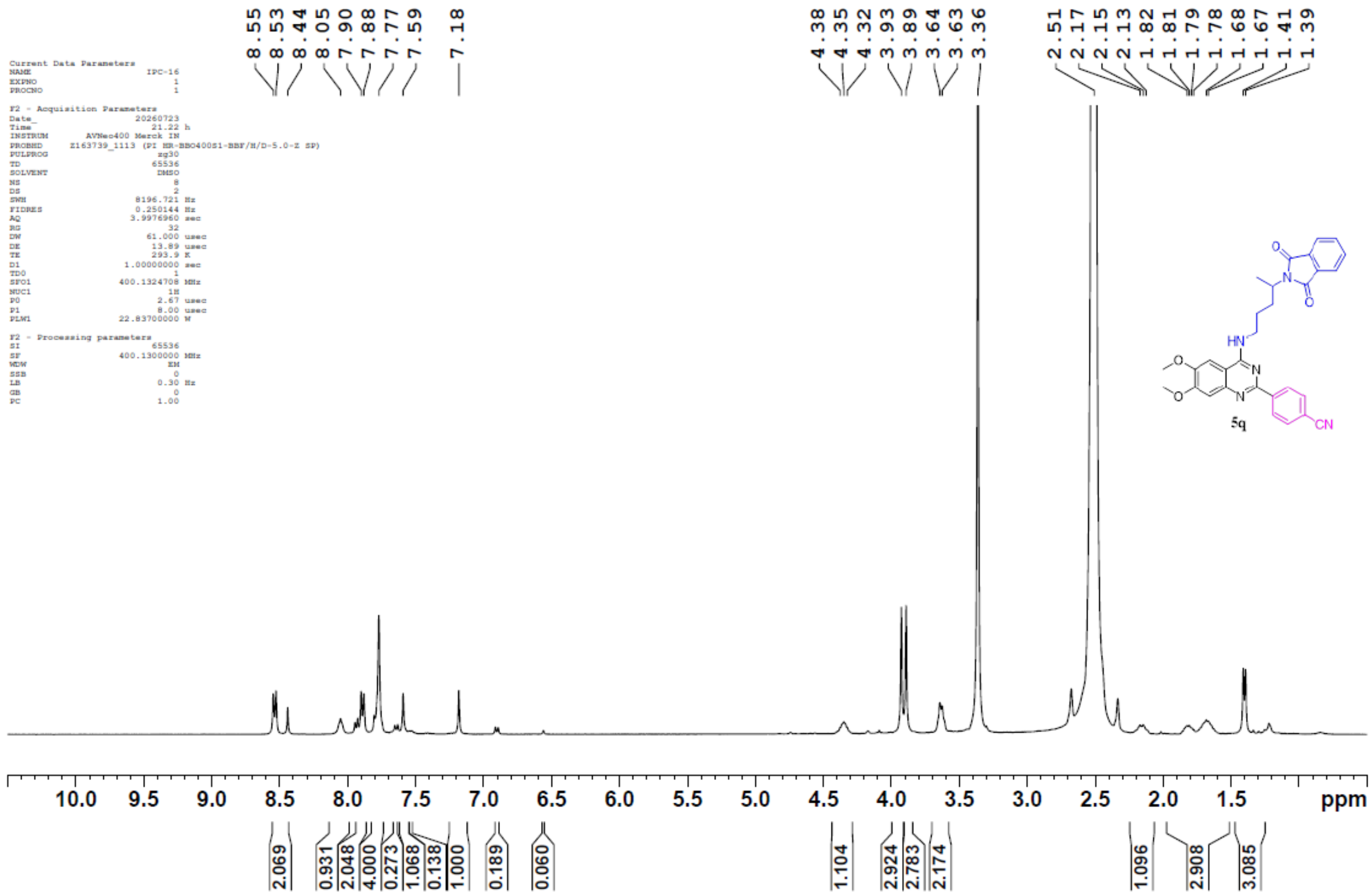

5q 13 NMR

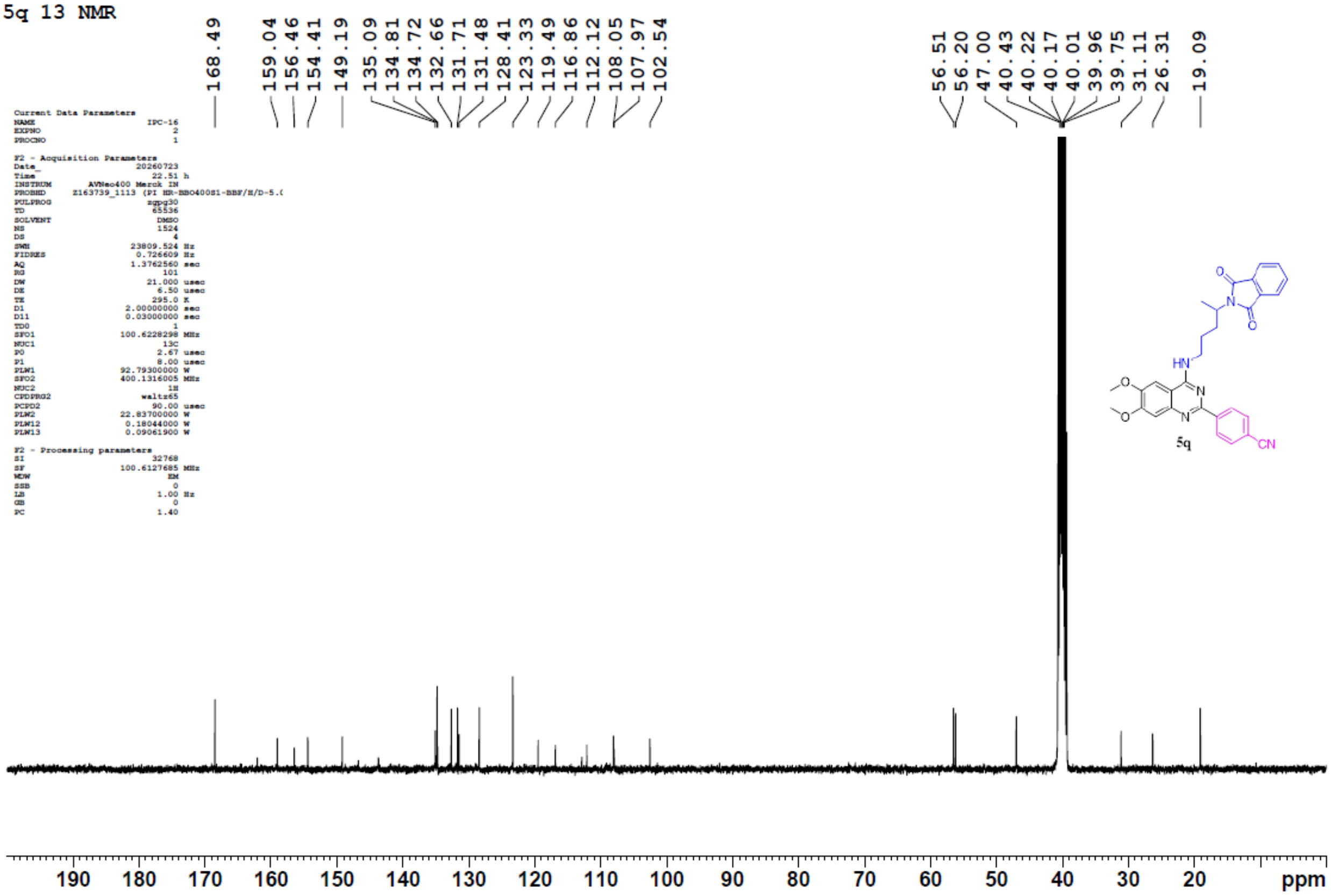

Supplement: Supplementary file 1 [file RA-OLF-D6RA08343A-s001.pdf]
